# Synthesis and Biological Evaluation of Fingolimod Derivatives as Antibacterial Agents

**DOI:** 10.1021/acsomega.1c02591

**Published:** 2021-07-09

**Authors:** Matej Zore, Shella Gilbert-Girard, Inés Reigada, Jayendra Z. Patel, Kirsi Savijoki, Adyary Fallarero, Jari Yli-Kauhaluoma

**Affiliations:** † Drug Research Program, Division of Pharmaceutical Chemistry and Technology, Faculty of Pharmacy, University of Helsinki, Viikinkaari 5 E, FI-00014 Helsinki, Finland; ‡ Drug Research Program, Division of Pharmaceutical Biosciences, Faculty of Pharmacy, University of Helsinki, Viikinkaari 5 E, FI-00014 Helsinki, Finland

## Abstract

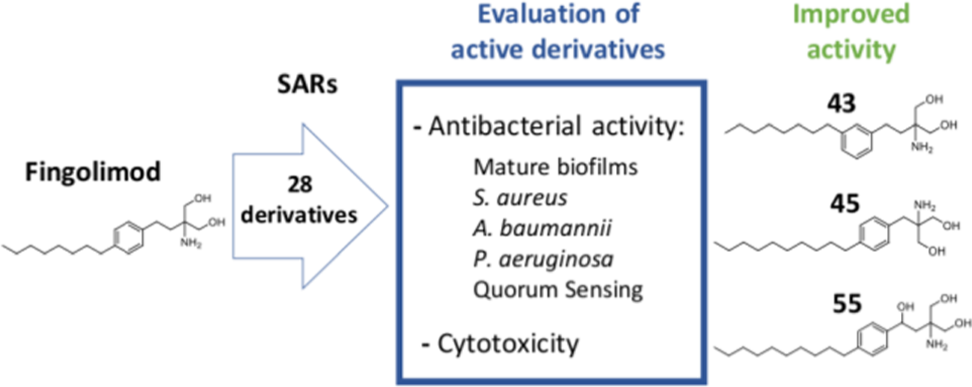

We recently identified fingolimod as a potent antibiofilm compound by screening FDA-approved drugs. To study if the antibacterial activity of fingolimod could be further improved and to explore in-depth structure–activity relationships, we synthesized 28 novel fingolimod derivatives and evaluated their efficacy against *Staphylococcus aureus* grown in planktonic/single cell and biofilms. The most effective derivatives were tested on preformed *S. aureus* biofilms and against Gram-negative bacteria *Acinetobacter baumannii* and *Pseudomonas aeruginosa*, using fingolimod as the reference compound. Seven derivatives were more effective against *S. aureus*, while five other derivatives showed improved activity against *P. aeruginosa* and/or *A. baumannii*, with no apparent change in cytotoxicity on human cells. The most interesting derivatives, compounds **43** and **55**, displayed a broader spectrum of antibacterial activity, possibly exerted by the change of the *para*-hydrocarbon chain to a meta position for **43** and by an additional hydroxyl group for **55**.

## Introduction

Bacterial infections are one of the most important challenges of modern medicine, as the emergence of multidrug-resistant strains increasingly reduces the options of antibiotic treatment.^1^ In particular, biofilm-forming organisms are responsible for an important number of recalcitrant infections.^2,3^ Biofilms, defined as multicellular communities protected by a self-produced matrix, are the most common phenotype of bacteria and are well known for their high tolerance to antibiotic treatments.^4^ They are particularly prevalent in device-related infections, which are increasing in numbers alongside the augmenting use of medical devices, and frequently cause the removal of the device.^5,6^ For these reasons, bacterial biofilms represent a large burden on the health care system and lead to significant morbidity and considerable costs.

**Figure 1:**
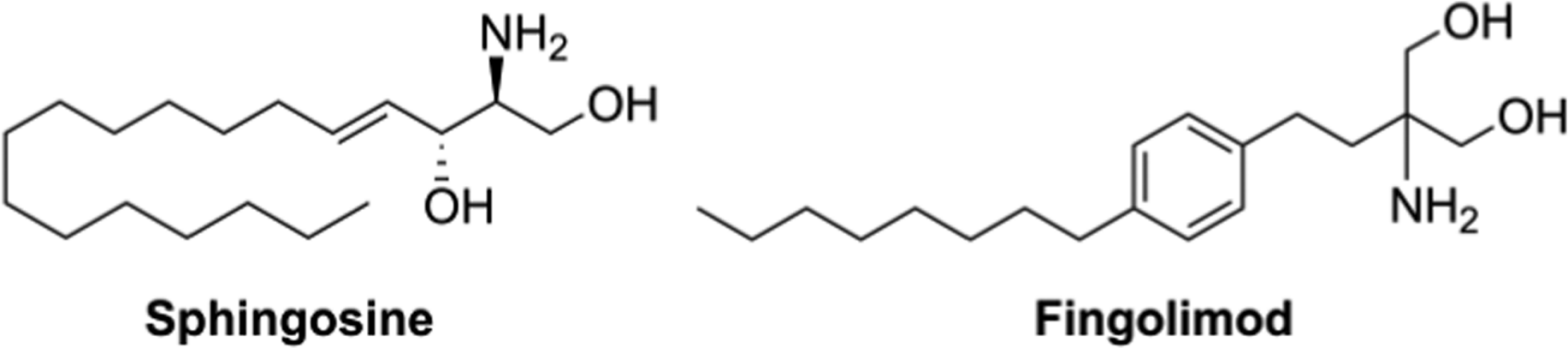
Structures of sphingosine and fingolimod.

Great efforts have been made in recent years to develop new treatment strategies and identify new antimicrobial compounds among natural and synthetic products as well as approved drugs. Sphingosine (Figure 1), a sphingoid base naturally found in mammalian cells, has a well-reported antimicrobial activity. This molecule plays a role in the immune protection of the skin and the airways, with its depletion contributing to infection.^7−9^ Sphingosine, along with other sphingolipids, has been tested against various bacterial species and its potential as an antibacterial treatment has been under evaluation in recent years.^10−13^ Treatment with sphingosine by inhalation or as a coating on endotracheal catheters has demonstrated a protective effect against *Pseudomonas aeruginosa* infection *in vivo*.^14,15^

Repurposing drugs as antimicrobials is an approach with many advantages, namely, that a large amount of information about the drug’s activity, pharmacokinetics, and safety is already available, facilitating its development for a new purpose.^16^ Recently, we identified fingolimod as an antibacterial compound in a screening of a library of FDA-approved drugs.^17^ Fingolimod is a sphingosine structural analogue that was initially synthesized from the natural fungal product myriocin.^18^ It was approved for the treatment of relapsing multiple sclerosis in view of its immunomodulatory activity that causes a redistribution of the lymphocyte population.^19^ Fingolimod’s antibacterial activity has been reported against a few bacterial species and, like sphingosine, it has shown a protective effect against *P. aeruginosain vivo*.^14,17,20^ As fingolimod and structurally similar compounds have strong activity against different bacterial species, we set out to synthesize fingolimod derivatives and evaluate their antibacterial effects, with a focus on their activity against biofilms. Fingolimod has been the focus of optimization efforts as a potential anticancer drug,^21,22^ but to our knowledge, this is the first report of the synthesis and evaluation of fingolimod derivatives as antibacterial compounds. The derivatives were first tested against *Staphylococcus aureus* grown in planktonic and biofilm states, and then the active compounds were further tested against preformed *S. aureus* biofilms and other pathogenic and clinically prevalent microorganisms such as *Acinetobacter baumannii* and *P. aeruginosa*. We studied the cytotoxicity effects of the most promising derivatives as well as their ability to interfere with the bacterial central cell-to-cell communication system (quorum sensing), a key mediator of biofilm formation and antibacterial resistance. Finally, we evaluated the effect of fingolimod on *S. aureus* biofilms formed in the presence of human neutrophils to emulate the *in vivo* conditions of an infection following the implantation of a medical device.

## Results and Discussion

### Chemistry

We designed and synthesized a library of 28 fingolimod derivatives by chemical modification of the aromatic ring, the eight-carbon chain, and the 2-amino-1,3-diol polar head. For the first set of derivatives (**10**–**16**, **25**–**29**, **33**), we focused mainly on the modification of the hydrocarbon chain and left the rest of the structure intact. We replaced the chain with additional aromatic structures bearing various substituents. The exceptions in this set are compounds **10** and **11**, which compared to fingolimod have shorter and longer carbon chains, respectively. Based on the screening of the first set of 13 derivatives, we designed a second set (**42**–**45**, **48**–**49**, **54**–**55**, **61**–**62**, **79**–**83**), and in this step, we focused on the modifications of the aromatic ring and the ethylene linker connecting the aromatic ring and polar head. Again, we left the amino diol moiety intact. All compounds in this set have a hydrocarbon chain, consisting of either eight or ten carbons.

**Scheme 1:**
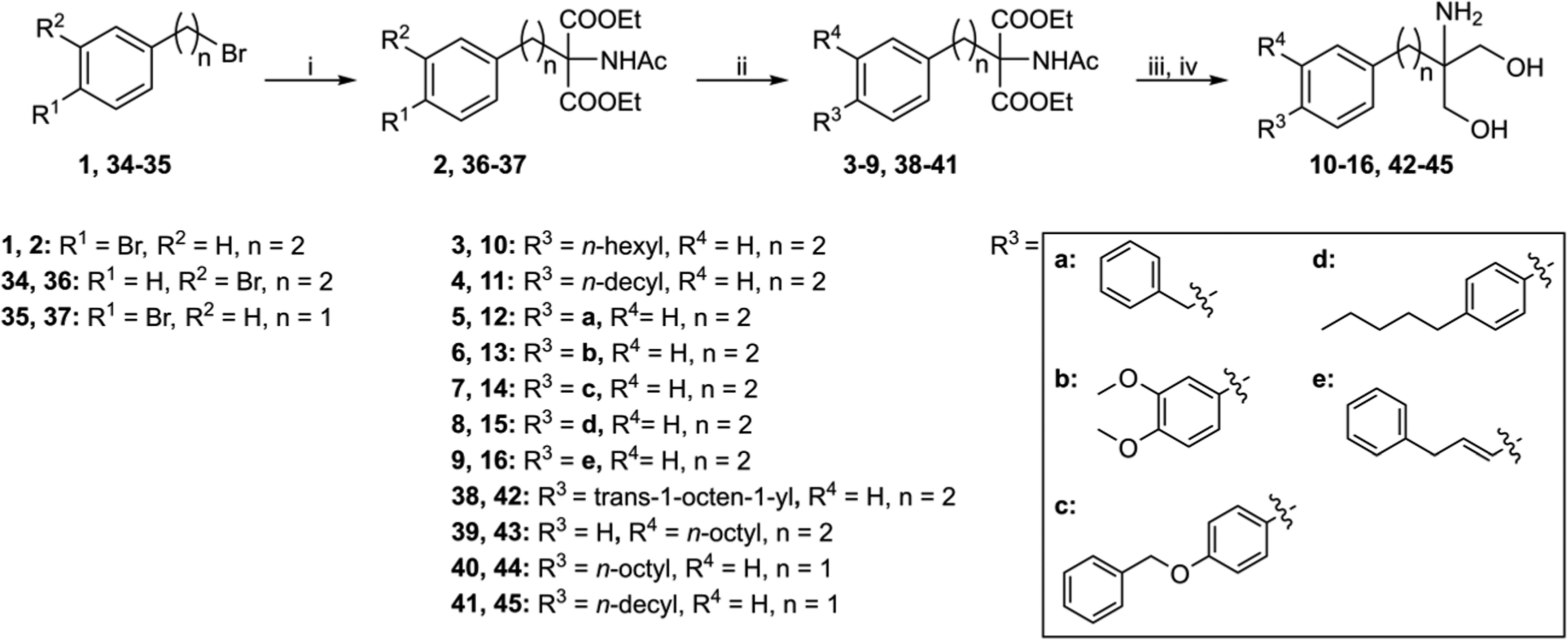
Synthesis of Fingolimod Derivatives 10–16 and 42–45 Reagents and conditions: (i) diethyl acetamidomalonate, Cs_2_CO_3_, MeCN, MW, 130 °C, 1 h; (ii) R^3^-B(OH)_2_ or R^4^-B(OH)_2_, Pd_2_(dba)_3_, SPhos, K_2_CO_3_, toluene/water (4:1), MW, 110 °C, 1.5–2 h; (iii) NaBH_4_, CaCl_2_, EtOH/water (4:1), rt, overnight; and (iv) 2 M LiOH (aq.), MeOH/tetrahydrofuran (THF) (1:1), reflux, 5 h.

**Scheme 2:**
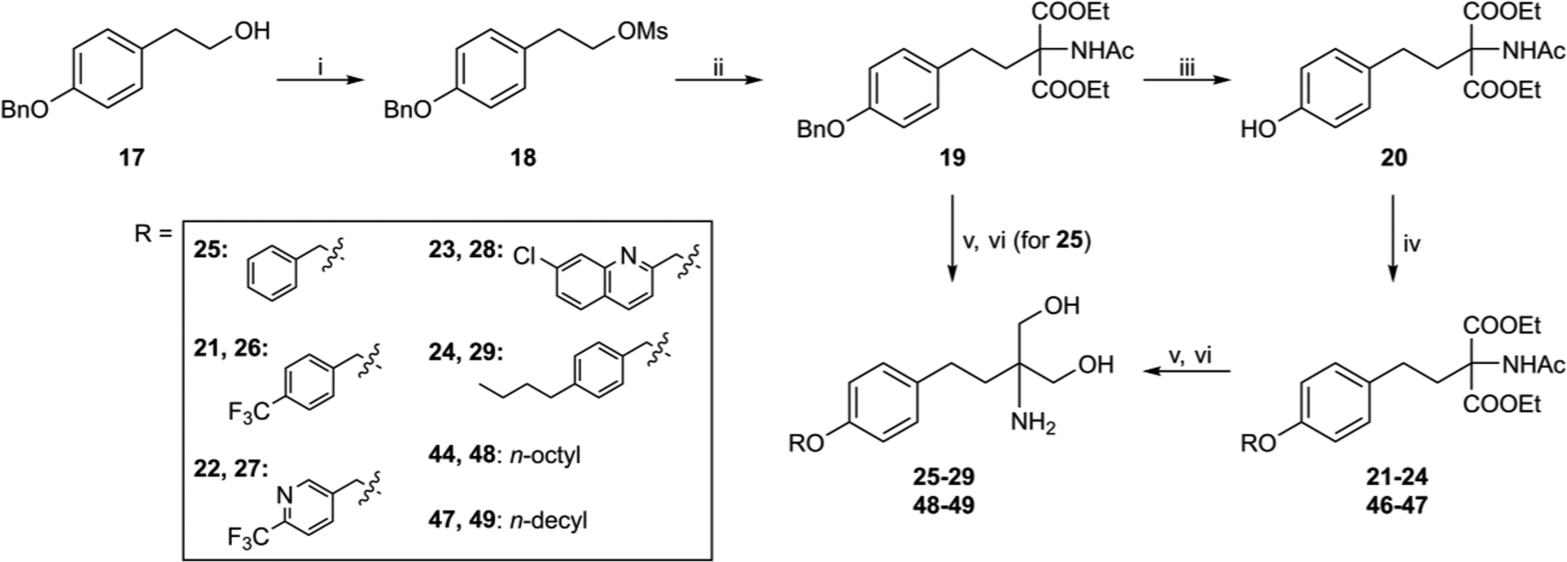
Synthesis of Phenyl Ether Derivatives **25**–**29**, **48**, and **49** Reagents and conditions: (i) MsCl, Et_3_N, dichloromethane (DCM), 0 °C, 2 h; (ii) diethyl acetamidomalonate, Cs_2_CO_3_, MeCN, MW, 130 °C, 1 h; (iii) H_2_, 10% Pd/C, EtOH/THF (1:1), rt, 5 h; (iv) R-Br, K_2_CO_3_, *N*,*N*–dimethylformamide (DMF), 0 °C to rt, overnight; (v) NaBH_4_, CaCl_2_, EtOH/water (4:1), rt, overnight; (vi) 2 M LiBH_4_, THF, rt, overnight (for **25** and **27**); and (vii) 2 M LiOH (aq.), MeOH/THF (1:1), reflux, 5 h.

**Scheme 3:**
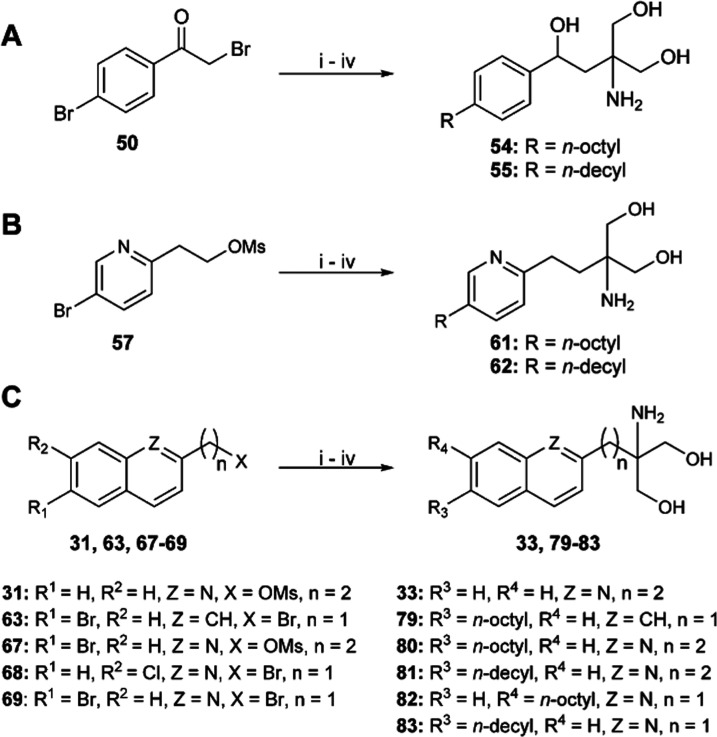
Synthesis of Derivatives **54**–**55** (A), Pyridine Derivatives **61**–**62** (B), and Quinoline and Naphthalene Derivatives **33** and **79**–**83** (C) Reagents and conditions: (i) diethyl acetamidomalonate, Cs_2_CO_3_, MeCN, or DMF, MW, 130 °C, 1 h; (ii) *n*-octylboronic acid or *n*-decylboronic acid, Pd_2_(dba)_3_, SPhos, K_2_CO_3_, toluene/water (4:1), MW, 110 °C, 1.5–8 h; (iii) NaBH_4_, CaCl_2_, EtOH/water (4:1), rt, overnight or 1 M LiAlH_4_, THF, 0 °C to rt, 2 h (for **80** and **81**); and (iv) 2 M LiOH (aq.), MeOH/THF (1:1), reflux, 5 h.

The four to six-step synthesis of all derivatives is presented in Schemes 1–3. A crucial step in the synthesis was the nucleophilic substitution of halide or mesylate with diethyl acetamidomalonate in the presence of cesium carbonate to obtain *para*-bromo N-acetylated diester intermediates **2**, **36**–**37**, **51**, **58**, and **70**–**73**, or N-acetylated diester phenol intermediate **20**.^23^ Bromo intermediates were later used for microwave-assisted Suzuki coupling with an appropriate boronic acid and palladium catalyst (Schemes 1 and 3), while a phenol intermediate was used for O-alkylation with appropriate alkyl bromide in the presence of potassium carbonate (Scheme 2). The last step in the synthesis of all derivatives was a reduction of the corresponding diester with sodium borohydride and calcium chloride, or lithium aluminum hydride to obtain N-acetylated diol intermediates, which were further hydrolyzed with lithium hydroxide (aq.) to give the desired compounds.^24^

### Screening of the Derivatives for Their Antibacterial Activity against *S. aureus*

The antibacterial activity of the synthesized compounds was first evaluated against *S. aureus* at a concentration of 50 μM (Supporting Information, Figure S1). Compounds that inhibited both the planktonic cells (turbidity and viability) and the biofilm formation (viability and biomass) by at least 60% were considered active. From the screening of the first set of 13 compounds (Figure S1A), we identified only two active compounds (**11** and **15**), which were then selected for further characterization. Compound **11** had the strongest inhibitory activity and was also the most structurally similar to fingolimod, with only two carbons added to its hydrocarbon chain. As opposed to the other derivatives with two aromatic rings, compounds **15** and **29** both possessed a short hydrocarbon chain. Yet, only **15** displayed high enough activity to be considered active. These results demonstrated the importance of the hydrocarbon chain for the antibacterial and antibiofilm activity, and its replacement led to decreased or lost inhibitory activity even at the concentration of 50 μM. In addition, the length of the alkyl chain proved important. Compound **10** had a shorter, six-carbon chain and possessed only limited activity against *S. aureus* biofilms. As the only derivatives with a measurable antibacterial activity were the ones with a hydrocarbon chain, and since fingolimod and **11**, the two compounds with the longest chains, displayed the highest inhibitory activity, the second step of optimization focused on derivative structures with this feature.

The antibacterial activity of the second set of derivatives was also first studied against *S. aureus* at 50 μM (Figure S2B). Among these, only pyridine derivative **61** did not show an inhibitory activity comparable to fingolimod and was therefore discarded. This finding was intriguing, as **62** had an identical structure, with a longer hydrocarbon chain, and yet displayed a higher activity. Including the two derivatives selected from the first screening (**11** and **15**), a total of 16 derivatives active against *S. aureus* were selected for further characterization.

### Concentration–Response of the Active Derivatives

**Figure 2:**
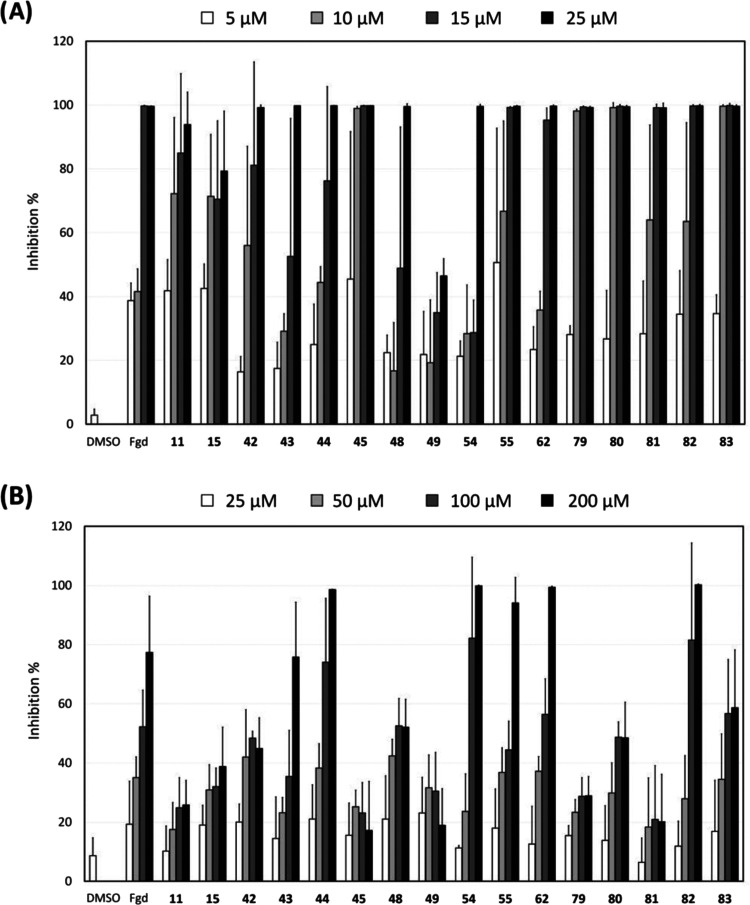
Inhibition of the viability of *S. aureus* ATCC 25923 biofilms in pre-exposure (A) and post-exposure (B) after exposure to fingolimod (Fgd) derivatives. The results are expressed as the inhibition percentage ± SD in comparison to the untreated control. The experiment was repeated three times, each with two replicates per concentration.

To determine the minimal inhibitory concentration (MIC), the active derivatives were tested at different concentrations against *S. aureus* ATCC 25923. Here, the MIC is defined as the lowest concentration where no growth was visible (resulting in over 90% inhibition of turbidity and viability of the planktonic cells) after 18 h of incubation. In addition, the compounds were tested in post-exposure for their capacity to affect a preformed biofilm (compounds added to 18-h-old biofilms and incubated for 24 h). Fingolimod was previously found to have an MIC of 15 μM in pre-exposure with some activity also against preformed biofilms.^17^ The same combination of four measurements used for the initial screening rounds (planktonic turbidity, planktonic viability, biofilm viability, and biofilm total biomass) was used in both pre- and post-exposure studies. Figure 2 shows the inhibition of the viability of *S. aureus* biofilms by the derivatives in either pre-exposure at 5–25 μM or post-exposure at 25–200 μM. The viability of the biofilms is shown as a representative parameter, and the other three measurements lead to similar conclusions regarding the activity of the derivatives. The inhibition percentages and standard deviation (SD) obtained for all measurements (viability and turbidity of the planktonic cells, viability, and biomass of the biofilms) are available as the Supporting Information (Table S1). In pre-exposure (Figure 2A), seven derivatives displayed higher inhibitory activity than fingolimod. Four derivatives, **45**, **79**, **80**, and **83**, had an MIC of 10 μM, and for some replicates, the MIC of **45** and **55** was as low as 5 μM. The derivatives **11** and **81** displayed an MIC varying from 10 to 15 μM (depending on the replicate), and **62** and **82** had the same MIC as fingolimod. Interestingly, wide variations in the antibacterial activity were observed between very similar structures, and in some cases, small structural changes led to a very drastic change of activity.

Generally, a longer hydrocarbon chain seemed beneficial as all derivatives with a 10-carbon chain (**11**, **45**, **49**, **55**, **62**, **81**, and **83**) had an MIC either equal or lower than fingolimod, except for **49**. As an example, derivatives **44** and **45**, which structurally differ only in the length of the chain, had an MIC of 15–25 and 5–10 μM, respectively. However, this did not apply to all comparable structures, as **80** and **81** had very similar activity and the compound with the shorter chain was slightly more potent. Compound **81**, like fingolimod, was not fully soluble in dimethyl sulfoxide (DMSO), which is likely to decrease its activity. Replacing the phenyl ring in fingolimod with quinoline or naphthalene seemed to either improve the activity or leave it unchanged. All derivatives with bicyclic aromatic rings performed the same as fingolimod (**81** and **82**) or better (**79**, **80**, and **83**). On the other hand, replacing the phenyl ring with pyridine negatively affected the inhibitory activity of derivative **61**. However, the longer chain of compound **62** compensated for this modification since the MIC was similar to fingolimod. A shorter carbon linker between the aromatic ring and the polar head did not seem to positively affect the activity in itself (**44** has lower activity than fingolimod), while not necessarily being correlated with a lower activity as some of the most potent derivatives harbored this modification (**45**, **79**, and **83**). Addition of another hydroxyl group next to the polar head decreased the inhibitory activity (**54**), although a longer hydrocarbon chain seems to compensate for this modification (**55**). Phenyl ether derivatives **48** and **49** had lower activity, indicating that the oxygen on the para position negatively affects the antibacterial activity. The *meta*-isomer (**43**) had slightly lower activity (MIC of 15–25 μM), suggesting that the position of the hydrocarbon chain plays a minor role against *S. aureus*. A combination of different structural changes in some derivatives might also affect the antibacterial activity in a more complex way that would be difficult to directly correlate to one or another change. In the case of **82** and **83**, several modifications were made compared to fingolimod; yet, the activity remained unchanged or even improved (**83**).

Very different results were obtained in post-exposure (Figure 2B), as the derivatives **44**, **54**, and **82** were the strongest inhibitors, affecting both the viability (Figure 2B) and total biomass (Table S1) of preformed biofilms by at least 70% at 100 μM. Considering that preformed biofilms display a greater tolerance to chemical agents, such a reduction is not negligible. Other derivatives showed activity comparable to fingolimod (**43**, **48**, **55**, **62**, **80**, and **83**) and caused an important inhibition of the preformed biofilms. As opposed to pre-exposure, a longer hydrocarbon chain did not improve the post-exposure activity. On the contrary, many derivatives with this feature that showed potent activity in pre-exposure had an important loss of activity in post-exposure. In addition, when comparing similar compounds with shorter and longer chains, the compounds with the shorter chain always performed better (e.g., fingolimod vs **11**, **44** vs **45**, etc.). A shorter linker to the polar head also appeared a positive change (fingolimod vs **44**), while a bicyclic aromatic ring did not seem to change the post-exposure activity. The addition of a hydroxyl group (**54**, **55**) also appeared to positively influence the activity. Interestingly, the derivatives that performed best in pre-exposure were quite different from those that did best in post-exposure, underlining how each setting’s conditions are greatly important in what type of structures will prove efficient.

### Activity of the Active Derivatives on Other Bacterial Strains

To verify if the derivatives that performed best against *S. aureus* ATCC 25923 could also show activity against other clinical strains, we tested six of the most effective derivatives (**45**, **55**, **79**, **80**, **82**, and **83**) against *S. aureus* ATCC 12598 (pathogenic septic arthritis isolate) and P2 (penicillin-resistant hip prosthetic implant isolate) at 5–10–15–25 μM as described above for planktonic and biofilm cells. The MIC values obtained for these derivatives against each *S. aureus* strain are shown in Table 1. All of the inhibition results against the clinical strains of *S. aureus* are available as the Supporting Information in Table S2. All six derivatives and fingolimod showed a similar and even increased activity in some cases against the clinical strains compared to *S. aureus* ATCC 25923. The derivatives **45** and **79** were the most potent, with an MIC between 5 and 10 μM, which exceeds MIC detected with the parent compound.

**Table 1:**
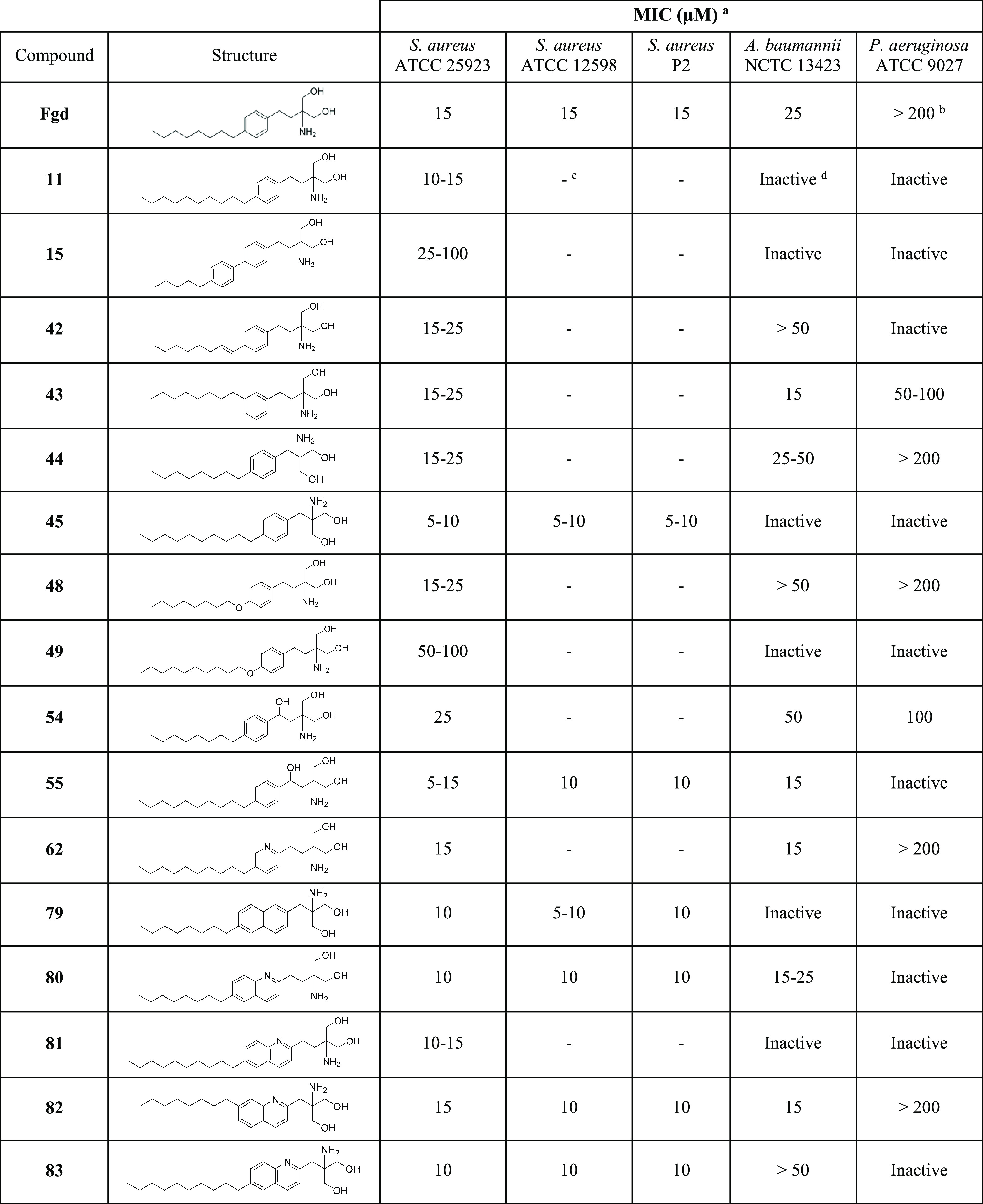
Minimum Inhibitory Concentration (MIC) of the 16 Selected Active Fingolimod Derivatives against all Strains Tested^d^

a The MIC is defined as the lowest concentration where no growth was visible (>90% inhibition of turbidity and viability of the planktonic cells). The experiment was repeated three times with two replicates per concentration.

b When some inhibitory activity was observed, but no MIC was reached, the MIC is indicated to be over the highest concentration tested.

c A dash indicates that the compound was not tested on the strain.

d “Inactive” indicates that no activity was observed up to the highest concentration tested (50 μM with *A. baumannii* and 200 μM with *P. aeruginosa*).

In addition, we previously reported that fingolimod shows strong activity against *A. baumannii* (MIC 25 μM) and modest activity against *P. aeruginosa*.^17^ These bacteria belong to the ESKAPE pathogens (*Enterococcus faecium*, *S. aureus*, *Klebsiella pneumoniae*, *A. baumannii*, *P. aeruginosa*, and *Enterobacter* species), causing a majority of nosocomial infections and having a great ability to evade treatment.^25^ Here, we tested the 16 derivatives selected from the initial screening against *A. baumannii* at 10–15–25–50 μM and against *P. aeruginosa* at 50–100–150–200 μM using the same protocol as above with necessary modifications, taking into account the Gram-negative nature of the tested species. *A. baumannii* NCTC 13423 is a multidrug-resistant strain^26^ and *P. aeruginosa* ATCC 9027 is a nonvirulent antibiotic-sensitive clinical strain.^27^ Fingolimod had previously shown the strongest activity against this strain out of a few *P. aeruginosa* strains.^17^ Derivatives were tested in pre-exposure, and Table 1 shows the MIC measured for each derivative. Where no MIC could be obtained, the results for the highest concentration tested are shown. All inhibition results are available as the Supporting Information in Table S3. Four derivatives (**43**, **55**, **62**, and **82**) displayed a clearly increased activity with an MIC of 15 μM against *A. baumannii*. The derivatives that were found the most effective against *A. baumannii* were not correlated to those detected as most effective against *S. aureus* in pre-exposure. The derivatives **55**, **80**, and **82** performed well on both strains, whereas **43** and **62** displayed improved activity only against *A. baumannii*. On the other hand, **11**, **45**, and **79**, compounds with high efficacy against *S. aureus*, were inactive at concentrations up to 50 μM against *A. baumannii*. Interestingly, **43** and **82**, which have the hydrocarbon chain at a different position compared to fingolimod, showed an improved activity. While a longer chain seemed beneficial against *S. aureus*, this was not necessarily the case against *A. baumannii*, and only two compounds with a 10-carbon chain (**55** and **62**) showed an improved activity. Other structural changes either did not affect the activity or did so negatively.

The activity of the derivatives against *P. aeruginosa* was also different from what was observed against *S. aureus* and *A. baumannii*. Two derivatives, **43** and **54**, displayed a clearly improved activity with an MIC of 50–100 μM, while fingolimod was not able to completely inhibit the bacteria at a concentration up to 200 μM. The reported activity of fingolimod and sphingosine against *P. aeruginosa* has been strongly variable depending on the strains tested and the experimental conditions,^10,14,17^ but as inhalation of fingolimod itself was shown to rescue susceptible mice from a *P. aeruginosa* lung infection, the derivatives **43** and **54** might be interesting compounds for similar use.^14^ For this species, a longer hydrocarbon chain has systemically led to a lower activity, as exemplified by **11**, **45**, and **55**, in comparison with fingolimod, **44** and **54**, respectively. A bicyclic aromatic ring has similarly reduced the activity. As observed with *A. baumannii*, *meta*-isomer (**43**) also performed better against *P. aeruginosa*, suggesting that meta-substitution is preferable against Gram-negative species. The addition of a hydroxyl group next to the polar head (**54**) also improved the inhibitory activity against *P. aeruginosa*, a modification that also improved the activity against *S. aureus* in post-exposure. Other modifications did not seem to visibly affect the activity of the compounds.

When comparing the activity of the derivatives against different species, very different conclusions can be made between the Gram-positive and Gram-negative species. Compounds with a longer hydrocarbon chain, bicyclic aromatic ring, and/or shorter carbon linker on the right side seem to increase the activity against *S. aureus*. Changing the position of the hydrocarbon chain or an additional hydroxyl group next to the polar head seems to broaden the spectrum of activity, as some of the derivatives bearing these modifications have a good activity also against Gram-negative species. The derivative **82** displayed similar activity compared to fingolimod against *S. aureus* in pre-exposure and better activity against *S. aureus* in post-exposure and against *A. baumannii*. Also, **43**, while having good activity against *S. aureus* in pre-exposure, was among the most active against the Gram-negative strains.

In a recent work, the mode of action of sphingosine as an antibacterial compound against *S. aureus* and *P. aeruginosa* was suggested to involve an interaction between sphingosine’s protonated amino group and a negatively charged protein, cardiolipin, in the bacterial membrane, causing clustering of the protein and thus compromising membrane’s permeability.^13^ The presence of the amino group in the structure was proven necessary for the antibacterial activity of sphingosine. Considering the structural similarity between fingolimod and sphingosine, it is likely that fingolimod uses the same mechanism, although our results also suggest that other parts of the structure, particularly the long hydrocarbon chain, are important for these molecules’ antibacterial activity.

### Activity of Selected Derivatives on Quorum Sensing

Quorum sensing (QS) is a community-based communication system that is activated when bacteria reach a high enough density, at which point QS induces community behaviors, such as the formation of a biofilm.^28^ Inhibiting QS is therefore a potential antivirulence approach to prevent biofilm formation and keep bacteria in their more susceptible planktonic state.^29^ The QS system molecules vary from species to species, and in Gram-negative bacteria, they are generally acyl-homoserine lactones (AHLs).^28^*Chromobacterium violaceum* can be used as a reporter for QS activation as its QS induces the production of a violet pigment, violacein.^30^ We previously reported that fingolimod could inhibit QS in *C. violaceum*.^17^ To test whether some of the derivatives here would show an improved QS inhibitory activity, we tested selected derivatives that performed well on various strains using *C. violaceum* as previously described.^17,31^

**Figure 3:**
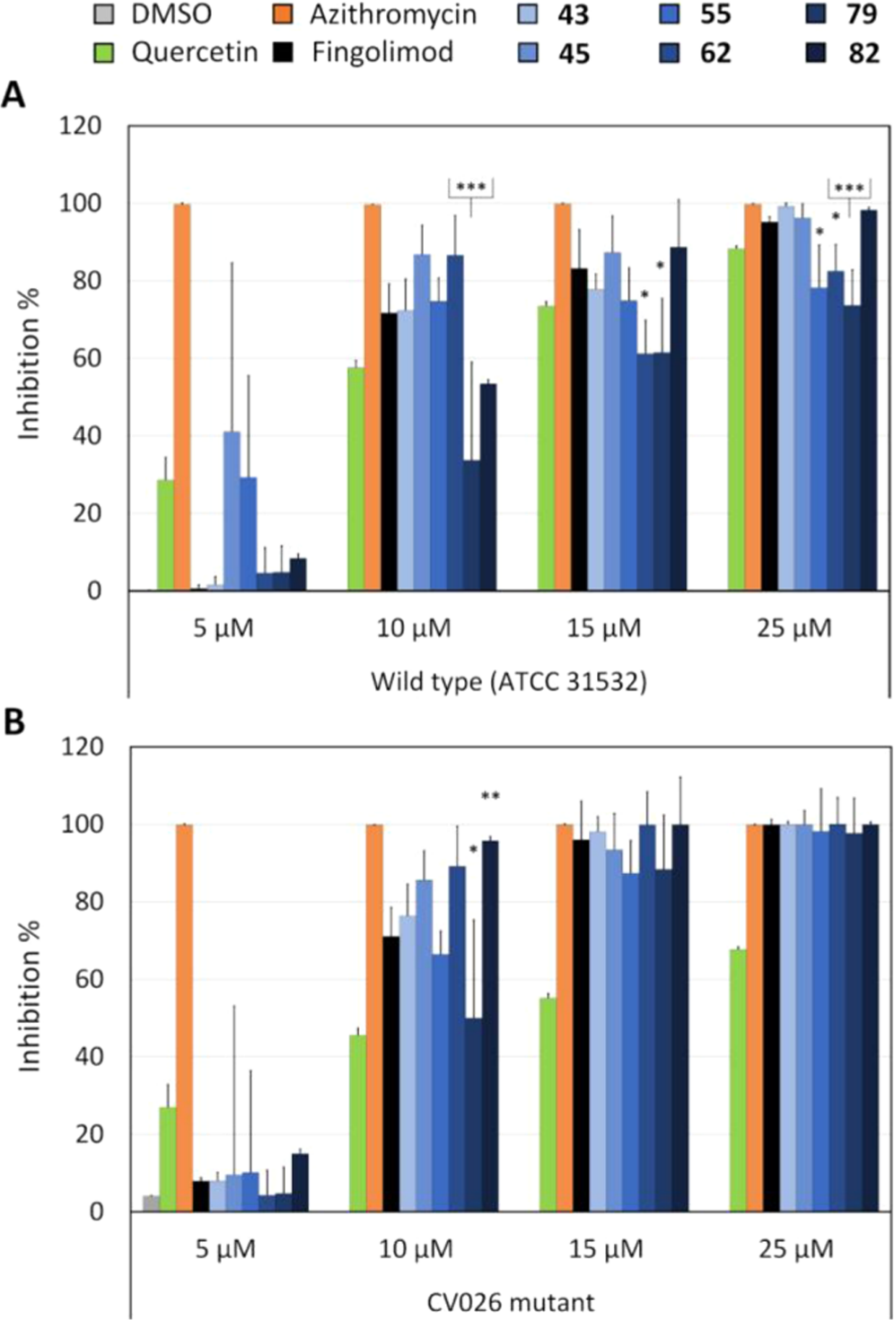
Quorum sensing inhibitory activity of selected fingolimod (Fgd) derivatives at various concentrations against (A) *C. violaceum* ATCC 31532 (wild type) and (B) *C. violaceum* CV026 (AHL-deficient mutant). The results are expressed as the inhibition percentage ± SD as compared to the untreated control. The experiment was repeated twice with two replicates per concentration. The stars indicate the significance of the difference between the derivatives and fingolimod, *: *p* < 0.05, **: *p* < 0.01, ***: *p* < 0.001.

The derivatives were tested against both the wild-type strain (ATCC 31532) and the CV026 mutant strain that needs the addition of AHL to activate QS.^30^ The use of both strains allows indicating if the QS inhibition takes place upstream or downstream of the AHL synthesis, for instance, in the case of quorum quenchers via binding to the AHL outside of the cells or by affecting the AHL diffusion through the cell wall/membrane. The viability of the bacteria was measured in parallel with a resazurin staining to assess if the inhibitory activity resulted from genuine anti-QS effects rather than bactericidal activity (Supporting information, Figure S2). Quercetin was used as a control for QS inhibition (QSI), as its activity has been reported previously,^32^ and azithromycin was used as a bactericidal control. Figure 3 shows the inhibitory effects of the selected derivatives and the controls on both the wild-type and the mutant strains. None of the derivatives displayed a bactericidal effect on either strain of *C. violaceum* (Figure S2). On the other hand, they promoted strong anti-QS effects with at least 60% inhibition at 15 μM on both strains. This indicates that the QS signal is inhibited downstream of the release of the AHL, potentially by quenching the signal or by competition with the AHL’s receptor. The inhibitory profile of the derivatives did not differ significantly from that detected with fingolimod. Intriguingly, **82** had a significantly better QSI activity against the mutant strain (*p* = 0.006). Derivative **79** was found to be an effective QSI, but displayed significantly lower QSI activity than fingolimod against the mutant strain at 10 μM *(p* = 0.021) and the wild-type strain at 10 μM (*p* < 0.001), 15 μM (*p* = 0.018), and 25 μM (*p* < 0.001). Although having a seemingly strong QSI activity against the wild-type strain at 10 μM, **62** performed significantly worse than fingolimod at 15 and 25 μM (*p* = 0.016 and 0.036, respectively). Since the QSI activity profiles of each derivative are highly similar, clear conclusions as to what structural changes benefit the QSI activity are hard to make.

### Cytotoxicity of Selected Derivatives

Fingolimod has been reported to have cytotoxic effects on various cancer cell lines with a CC_50_ value in the range of 5–20 μM.^33,34^ To see if some of the structural changes made on the derivatives could ameliorate this, the same six derivatives selected for the QSI experiment were also tested on three cell lines, A549, HEp-2, and HL-60. Table 2 shows the CC_50_ values obtained for each cell line after exposure to the compounds. In contrast with previous reports, fingolimod had a CC_50_ value above or close to the highest concentration tested (40 μM), possibly due to different incubation times and poor solubility. None of the derivatives displayed reduced cytotoxicity in comparison to fingolimod, but **43** and **79** both displayed a lesser cytotoxic activity than the other derivatives and were quite similar to fingolimod. All compounds, including fingolimod, had CC_50_ at a higher concentration than their MIC against *S. aureus* and *A. baumannii*, offering a potential therapeutic window for antibacterial treatment. While many derivatives have shown an increased antibacterial activity, fingolimod also remains an interesting potential antibacterial compound as it is less cytotoxic. In addition, it has been reported that only the nonphosphorylated form of fingolimod is cytotoxic, while the phosphorylated compound might even have a cytoprotective effect.^35,36^ As fingolimod, and possibly some of its derivatives, become phosphorylated *in vivo*, it is possible that they would prove a safe treatment option, something that could be confirmed by future *in vivo* studies.

**Table 2:** CC_50_ of Fingolimod and Six Derivatives on Three Human Cell Lines (A549, HEp-2, and HL-60), after a 24 h Incubation

	CC_50_ (μM)^a^		
	A549	HEp-2	HL-60
fingolimod	>40	>40	36.1 ± 13.4
43	>40	>40	23.4 ± 3.2
45	27.4 ± 15.1	>40	3.2 ± 1
55	35.3 ± 5.1	33.9 ± 1.2	7.4 ± 6.9
62	27.6 ± 6.4	30.9 ± 6.2	3.6 ± 0.4
79	>40	>40	26.9 ± 1.6
82	27.5 ± 3	28.8 ± 1.8	6.5 ± 0.8

a Calculated from the results of four experiments with each two technical replicates per concentration (two experiments for HL-60).

### Effect of Fingolimod on *S. aureus* in Coculture with Human Neutrophils

As biofilms are particularly prevalent in device-associated infection, a potential application for fingolimod and its derivatives could be the protection of medical devices against biofilm formation. Many factors can affect the activity of a drug *in vivo*, for instance, the presence of human cells and their by-products as well as inflammation and tissue damage following the insertion of a medical device. The placement of endotracheal tubes results in tissue injuries that induce the influx of neutrophils, increasing their number by up to 10-fold in the trachea after intubation.^37,38^ This acute inflammatory response may lead to the ineffectiveness of the innate immune system to clear out planktonic bacteria, giving a perfect opportunity for biofilm formation.^39^ Fingolimod being the least cytotoxic compound in our study, we used it in a proof-of-concept model to show how it would protect a catheter made of clinically relevant material, low-density polyethylene (LDPE), from biofilm formation in a less simplified environment. We used a coculture system that includes HL-60 cells differentiated into neutrophils and *S. aureus* ATCC 25923.^40^

**Figure 4:**
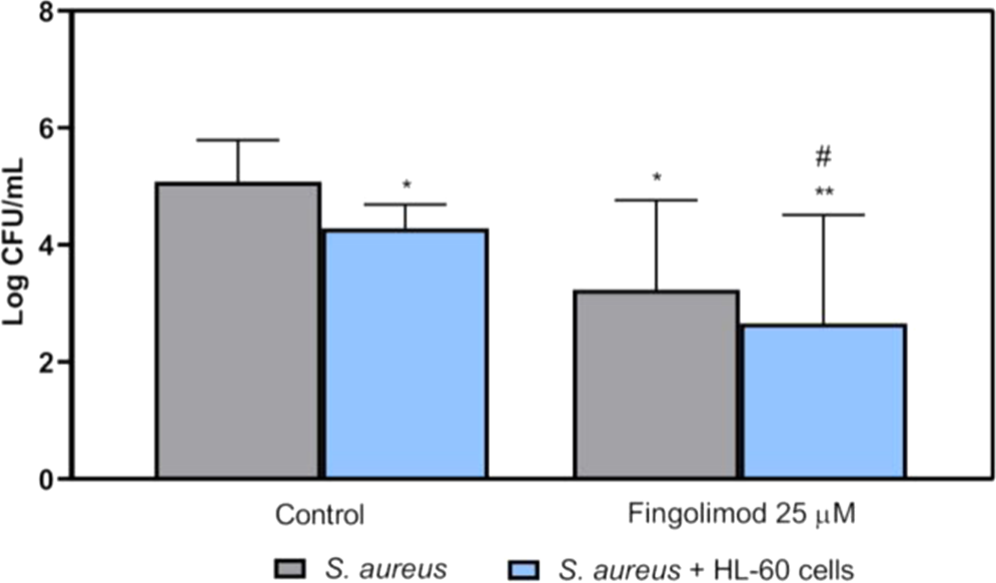
Viable counts of adhered *S. aureus* ATCC 25923 on LDPE tubes, grown alone or in coculture with differentiated HL-60 cells and exposed for 24 h to fingolimod at 25 μM. The results are expressed as mean + SD of three technical replicates in experiments repeated three times. * Indicates differences with the monoculture control, # indicates differences with the coculture control (*: *p* < 0.05, **: *p* < 0.01, #: *p* < 0.05).

Figure 4 shows the effects of fingolimod (25 μM) on the prevention of *S. aureus* ATCC 25923 attachment on LDPE tubes after a 24 h incubation. Fingolimod alone (in the absence of HL-60 cells) significantly reduced the numbers of attached viable *S. aureus* (*p* = 0.013). Adding the HL-60 cells to the system resulted in a significant reduction of attached *S. aureus* (*p* = 0.023). The addition of fingolimod in combination with HL-60 cells also caused a significant reduction of the bacterial attachment in comparison with the coculture control (*p* = 0.042). Fingolimod and the neutrophils seemed to act in synergy against the bacteria as the combination of the two had a slightly stronger effect than fingolimod or the cells alone. This showed that fingolimod, and quite probably its derivatives, are still active against *S. aureus* and has a protective activity on the catheter even in the presence of cells.

## Conclusions

In this work, we developed a library of 28 fingolimod derivatives and evaluated their antibacterial and antibiofilm activities. Compared to fingolimod, seven compounds displayed a better activity against *S. aureus*, with an MIC below 15 μM. Five derivatives were also more active against preformed *S. aureus* biofilms. On the other hand, five derivatives showed improved activity against *P. aeruginosa* and/or *A. baumannii*. Some of the most interesting compounds in our study include **43**, the *meta*-isomer of fingolimod, which was the most active against the Gram-negative species and was one of the least cytotoxic derivatives. Derivative **55** had an increased activity against *S. aureus* in both pre- and post-exposure and against *A. baumannii*. The derivative **45** was the most active against *S. aureus* in pre-exposure while showing poor activity in other conditions. By contrast, compound **54**, while having a lower MIC of 25 μM against *S. aureus*, had improved activity in post-exposure and good activity against *P. aeruginosa*. This study presents many modifications of the structure of fingolimod that affected its antibacterial activity in many interesting ways. While none of the tested derivatives displayed lower cytotoxicity than fingolimod, many optimization efforts generally do not lead to both an improvement of the desired activity and reduced cytotoxicity. Nevertheless, the information gathered in this work can guide further optimization toward less cytotoxic and more potent antibacterial molecules. This family of structures, which have gained more and more attention in recent years, have promising antibacterial and QSI activities, among other potential therapeutic uses.

## Experimental Section

### Chemistry

All reagents used in the synthesis were acquired from Merck (Darmstadt, Germany), Fluorochem (Hadfield, United Kingdom), Combi-Blocks (San Diego), and TCI (Tokyo, Japan) and used without further purification. Moisture-sensitive reactions were conducted using dry solvents in oven-dried (120 °C, >24 h) glassware under an inert argon atmosphere. The progress of chemical reactions was monitored by thin-layer chromatography (TLC) on 0.2 mm Silica gel 60 F_254_ aluminum plates (Merck, Darmstadt, Germany) and visualized by UV light (254/366 nm). Microwave reactions were conducted with a Biotage Initiator + SP Wave Microwave Synthesizer (Uppsala, Sweden). Column chromatography was carried out on automated Biotage Isolera Spektra Systems with ACI and Assist (ISO-1SW Isolera One) equipped with a variable UV–VIS (200–800 nm) photodiode array (Uppsala, Sweden), using preloaded Biotage SNAP and Sfär columns, and the indicated mobile phase gradient. Nuclear magnetic resonance spectra (^1^H, ^13^C, and ^19^F NMR) were recorded on a Bruker Ascend 400 MHz—Avance III HD NMR spectrometer (Bruker Corporation, Billerica, MA). Chemical shifts (δ) are reported in parts per million (ppm) relative to the residual NMR solvent signals: CDCl_3_ 7.26 and 77.16 ppm, CD_3_OD 3.31 and 49.00 ppm, DMSO-*d*_6_ 2.50 and 39.52 ppm, (CD_3_)_2_CO 2.05 and 29.84/206.26 for ^1^H and ^13^C NMR, respectively. When necessary, two-dimensional NMR spectra (HSQC, HMBC, COSY) were recorded to support structure determination. Multiplicities of peaks are represented as s (singlet), d (doublet), t (triplet), q (quartet), p (pentet), dd (doublet of doublets), m (multiplet), and bs (broad singlet). Coupling constants *J* are quoted in hertz (Hz). All spectra were processed for recorded FID files with MestReNova 12.0.1 software (Mestrelab Research, Santiago de Compostela, Spain). The exact mass and purity (>95%) of all tested compounds were confirmed by liquid chromatography–mass spectrometry (LC–MS) analyses with a Waters Acquity1 UPLC system (Waters, Milford, MA) equipped with an Acquity UPLC1 BEH C18 column (1.7 μm, 50 mm × 2.1 mm, Waters, Ireland), an Acquity PDA detector, and a Waters Synapt G2 HDMS mass spectrometer (Waters, Milford, MA) via an electrospray ionization (ESI) ion source in a positive mode. High-resolution mass spectrometry (HRMS-ESI) data were reported for the molecular ions [M + H]^+^.

### General Procedure A: Diethyl acetamidomalonate Alkylation

To a solution of diethyl acetamidomalonate (DEAM) and bromide or mesylate (typically 1.2–2 equiv) in anhydrous MeCN or DMF (0.1–0.2 M) was added cesium carbonate (Cs_2_CO_3_, 1.5 or 2.0 equiv), and the reaction mixture was irradiated in a microwave reactor for 1 h at 130 °C. The progress of the reaction was monitored by TLC using 50% EtOAc in *n*-heptane as a mobile phase. The mixture was filtered and concentrated on a rotary evaporator. The crude product was purified by silica gel column chromatography with an increasing gradient of EtOAc (starting with 10–30%) in *n*-heptane to afford the desired N-acetylated diester.

### General Procedure B: Suzuki Coupling

Aryl bromide or aryl chloride, boronic acid (1.2 equiv), 5 mol % of tris(dibenzylideneacetone)dipalladium(0) (Pd_2_(dba)_3_), and 10 mol % of 2-dicyclohexylphosphino-2′,6′-dimethoxybiphenyl (SPhos) were dissolved under argon in toluene/water (4:1; 0.1–0.2 M). When the starting material did not dissolve completely, a few drops of DMF were added. The reaction mixture was bubbled with argon for 1 min. Then, potassium carbonate (K_2_CO_3_, 3.0 equiv) was added, and the reaction mixture was irradiated in a microwave reactor for 1.5–8 h at 110 °C. The progress of the reaction was monitored by TLC using 50% EtOAc in *n*-heptane as a mobile phase. The mixture was filtered through Celite and concentrated on a rotary evaporator. The crude product was purified by silica gel column chromatography with an increasing gradient of EtOAc in *n*-heptane, starting with 10 or 30% of EtOAc.

### General Procedure C: O-Alkylation of Diethyl 2-Acetamido-2-(4-hydroxyphenethyl)malonate (20)

To a cooled solution of diethyl 2-acetamido-2-(4-hydroxyphenethyl)malonate (**20**) in anhydrous DMF was added anhydrous K_2_CO_3_ (2.0 equiv), and the mixture was stirred for 30 min on an ice bath. Appropriate bromide (1.2 equiv) was dissolved in anhydrous DMF, and the resulting solution was added dropwise to the reaction at 0 °C. Stirring was continued for 30 min, after which the reaction mixture was allowed to warm up to room temperature and stirred overnight. The progress of the reaction was monitored by TLC using 50% EtOAc in *n*-heptane as a mobile phase. The reaction was quenched with water and extracted with EtOAc. Combined organic layers were washed with brine, dried over anhydrous sodium sulfate (Na_2_SO_4_), filtered, and concentrated on a rotary evaporator. The crude product was purified by silica gel column chromatography with an increasing gradient of EtOAc in *n*-heptane, starting with 20% of EtOAc.

### General Procedure D: Diester Reduction and the Subsequent Amide Hydrolysis

To a solution of diester in EtOH/water (4:1), sodium borohydride (NaBH_4_, 5.0 equiv) and calcium chloride (CaCl_2_, 2.5 equiv) were added in portions over 1 h, and the reaction mixture was stirred at room temperature overnight. The progress of the reaction was monitored by TLC-NH_2_ using 5% MeOH in DCM as a mobile phase. If needed, excess quantities of NaBH_4_ (5.0 equiv) and CaCl_2_ (2.5 equiv) were added and the mixture was stirred for an additional 2–4 h. The reaction was quenched with water or a 0.1 M solution of HCl in water and extracted with either EtOAc or DCM. Combined organic layers were washed with brine, dried over anhydrous Na_2_SO_4_, filtered, and concentrated on a rotary evaporator to give an N-acetylated diol intermediate, which was used in the next step without further purification. To a solution of N-acetylated diol intermediate in MeOH/THF (1:1), a 2 M solution of lithium hydroxide (LiOH) in water was added and the reaction mixture was refluxed 4–7 h. The progress of the reaction was monitored by TLC-NH_2_ using 5% MeOH in DCM as a mobile phase. The mixture was cooled to room temperature and portioned between water and EtOAc or DCM. The layers were separated, and the aqueous layer was extracted with either EtOAc or DCM. The combined organic layers were washed with brine, dried over anhydrous Na_2_SO_4_, filtered, and concentrated on a rotary evaporator. Silica gel column chromatography was performed with a Biotage SNAP KP-NH column and an increasing gradient of MeOH in DCM, starting with 2% of MeOH, to afford the desired amino diol compounds.

#### Diethyl 2-Acetamido-2-(4-bromophenethyl)malonate (**2**)

General procedure A was followed using 4-bromophenethyl bromide (**1**) (2.00 g, 7.58 mmol, 1.5 equiv), DEAM (1.10 g, 5.05 mmol), Cs_2_CO_3_ (2.47 g, 7.58 mmol, 1.5 equiv), and anhydrous MeCN (20 mL). Compound **2** was obtained as a light yellow solid (1.16 g, 58%). ^1^H NMR (400 MHz, CDCl_3_): δ 7.40–7.35 (m, 2H), 7.04–6.99 (m, 2H), 6.75 (bs, 1H), 4.27–4.15 (m, 4H), 2.69–2.62 (m, 2H), 2.47–2.40 (m, 2H), 1.99 (s, 3H), 1.24 (t, *J* = 7.1 Hz, 6H). ^13^C NMR (101 MHz, CDCl_3_): δ 169.2, 168.1, 139.7, 131.5, 130.3, 120.0, 66.4, 62.8, 33.4, 29.7, 23.1, 14.1. HRMS-ESI (*m*/*z*): calcd for C_17_H_23_NO_5_Br [M + H]^+^ 400.0760, found: 400.0760.

#### Diethyl 2-Acetamido-2-(4-hexylphenethyl)malonate (**3**)

General procedure B was followed using **2** (0.100 g, 0.250 mmol), *n*-hexylboronic acid (39.0 mg, 0.300 mmol, 1.2 equiv), Pd_2_(dba)_3_ (11.4 mg, 0.0125 mmol, 0.05 equiv), SPhos (10.3 mg, 0.0250 mmol, 0.1 equiv), K_2_CO_3_ (0.104 g, 0.750 mmol, 3.0 equiv), and toluene/water (4:1, 2 mL). Compound **3** was obtained as a white solid (65 mg, 64%). ^1^H NMR (400 MHz, CDCl_3_): δ 7.11–7.01 (m, 4H), 6.75 (s, 1H), 4.28–4.11 (m, 4H), 2.72–2.64 (m, 2H), 2.55 (t, *J* = 7.6 Hz, 2H), 2.49–2.41 (m, 2H), 1.97 (s, 3H), 1.63–1.51 (m, 2H), 1.36–1.20 (m, 12H), 0.94–0.84 (m, 3H). ^13^C NMR (101 MHz, CDCl_3_): δ 169.1, 168.2, 140.9, 137.8, 128.5, 128.4, 66.6, 62.7, 35.7, 33.5, 31.9, 31.7, 29.8, 29.1, 23.1, 22.7, 14.2, 14.1. HRMS-ESI (*m*/*z*): calcd for C_23_H_36_NO_5_ [M + H]^+^ 406.2593, found: 406.2594.

#### Diethyl 2-Acetamido-2-(4-decylphenethyl)malonate (**4**)

General procedure B was followed using **2** (0.100 g, 0.250 mmol), *n*-decylboronic acid (46.9 mg, 0.300 mmol, 1.2 equiv), Pd_2_(dba)_3_ (11.4 mg, 0.0125 mmol, 0.05 equiv), SPhos (10.3 mg, 0.0250 mmol, 0.1 equiv), K_2_CO_3_ (0.104 mg, 0.750 mmol, 3.0 equiv), and toluene/water (4:1, 2 mL). Compound **4** was obtained as a white solid (68 mg, 59%). ^1^H NMR (400 MHz, CDCl_3_): δ 7.11–7.01 (m, 4H), 6.74 (bs, 1H), 4.26–4.12 (m, 4H), 2.72–2.64 (m, 2H), 2.54 (t, *J* = 7.6 Hz, 2H), 2.49–2.41 (m, 2H), 1.97 (s, 3H), 1.56 (m, 2H), 1.31–1.20 (m, 20H), 0.92–0.84 (m, 3H). ^13^C NMR (101 MHz, CDCl_3_): δ 169.0, 168.1, 140.8, 137.7, 128.4, 128.3, 66.4, 62.5, 35.5, 33.3, 31.9, 31.6, 29.7, 29.64, 29.61, 29.5, 29.3, 23.0, 22.7, 14.1, 14.0. HRMS-ESI (*m*/*z*): calcd for C_27_H_44_NO_5_ [M + H]^+^ 462.3219, found: 462.3222.

#### Diethyl 2-Acetamido-2-(4-benzylphenethyl)malonate (**5**)

General procedure B was followed using **2** (0.100 g, 0.250 mmol), benzylboronic acid pinacol ester (65.4 mg, 0.300 mmol, 1.2 equiv), Pd_2_(dba)_3_ (11.4 mg, 0.0125 mmol, 0.05 equiv), SPhos (10.3 mg, 0.0250 mmol, 0.1 equiv), K_2_CO_3_ (0.104 mg, 0.750 mmol, 3.0 equiv), and toluene/water (4:1, 2 mL). Compound **5** was obtained as a white solid (65 mg, 64%). ^1^H NMR (400 MHz, CDCl_3_): δ 7.30–7.23 (m, 2H), 7.21–7.14 (m, 3H), 7.11–7.03 (m, 4H), 6.73 (s, 1H), 4.25–4.09 (m, 4H), 3.93 (s, 2H), 2.72–2.63 (m, 2H), 2.49–2.41 (m, 2H), 1.95 (s, 3H), 1.22 (t, *J* = 7.1 Hz, 6H). ^13^C NMR (101 MHz, CDCl_3_): δ 169.1, 168.2, 141.4, 139.1, 138.4, 129.03, 128.97, 128.7, 128.6, 126.2, 66.5, 62.7, 41.7, 33.4, 29.8, 23.1, 14.1. HRMS-ESI (*m*/*z*): calcd for C_24_H_30_NO_5_ [M + H]^+^ 412.2124, found: 412.2124.

#### Diethyl 2-Acetamido-2-[2-(3′,4′-dimethoxy-[1,1′-biphenyl]-4-yl)ethyl]malonate (**6**)

General procedure B was followed using **2** (0.100 g, 0.250 mmol), (3,4-dimethoxyphenyl)boronic acid (54.6 mg, 0.300 mmol, 1.2 equiv), Pd_2_(dba)_3_ (11.4 mg, 0.0125 mmol, 0.05 equiv), SPhos (10.3 mg, 0.025 mmol, 0.1 equiv), K_2_CO_3_ (103.7 mg, 0.750 mmol, 3.0 equiv), and toluene/water (4:1, 1.5 mL). Compound **6** was obtained as a yellow solid (0.10 g, 87%). ^1^H NMR (400 MHz, CDCl_3_): δ 7.48–7.43 (m, 2H), 7.23–7.17 (m, 2H), 7.11 (dd, *J* = 8.3, 2.1 Hz, 1H), 7.07 (d, *J* = 2.1 Hz, 1H), 6.93 (d, *J* = 8.3 Hz, 1H), 6.78 (s, 1H), 4.28–4.16 (m, 4H), 3.94 (s, 3H), 3.91 (s, 3H), 2.76–2.68 (m, 2H), 2.56–2.48 (m, 2H), 1.99 (s, 3H), 1.26 (t, *J* = 7.1 Hz, 6H). ^13^C NMR (101 MHz, CDCl_3_): δ 169.2, 168.2, 149.3, 148.7, 139.4, 139.1, 134.1, 129.0, 126.9, 119.3, 111.6, 110.5, 66.5, 62.7, 56.12, 56.07, 33.5, 29.9, 23.1, 14.1. HRMS-ESI (*m*/*z*): calcd for C_25_H_32_NO_7_ [M + H]^+^ 458.2179, found: 458.2180.

#### Diethyl 2-Acetamido-2-[2-[4′-(benzyloxy)-[1,1′-biphenyl]-4-yl]ethyl]malonate (**7**)

General procedure B was followed using **2** (50.0 mg, 0.125 mmol), [4-(benzyloxy)phenyl]boronic acid (34.2 mg, 0.150 mmol, 1.2 equiv), Pd_2_(dba)_3_ (5.7 mg, 0.0063 mmol, 0.05 equiv), SPhos (5.1 mg, 0.013 mmol, 0.1 equiv), K_2_CO_3_ (51.8 mg, 0.375 mmol, 3.0 equiv), and toluene/water (4:1, 2 mL). Compound **7** was obtained as a yellow-white solid (52 mg, 82%). ^1^H NMR (400 MHz, CDCl_3_): δ 7.53–7.43 (m, 6H), 7.43–7.37 (m, 2H), 7.36–7.31 (m, 1H), 7.24–7.15 (m, 2H), 7.08–6.99 (m, 2H), 6.78 (s, 1H), 5.11 (s, 2H), 4.30–4.14 (m, 4H), 2.78–2.68 (m, 2H), 2.57–2.48 (m, 2H), 1.99 (s, 3H), 1.26 (t, *J* = 7.1 Hz, 6H). ^13^C NMR (101 MHz, CDCl_3_): δ 169.2, 168.2, 158.4, 139.2, 137.1, 133.9, 129.0, 128.8, 128.1, 127.6, 126.8, 115.3, 70.2, 66.5, 62.7, 33.4, 29.0, 23.1, 14.1. HRMS-ESI (*m*/*z*): calcd for C_30_H_34_NO_6_ [M + H]^+^ 504.2386, found: 504.2383.

#### Diethyl 2-Acetamido-2-[2-(4′-pentyl-[1,1′-biphenyl]-4-yl)ethyl]malonate (**8**)

General procedure B was followed using **2** (90.0 mg, 0.225 mmol), 4-pentylphenylboronic acid (51.9 mg, 0.270 mmol, 1.2 equiv), Pd_2_(dba)_3_ (10.3 mg, 0.0113 mmol, 0.05 equiv), SPhos (9.2 mg, 0.023 mmol, 0.1 equiv), K_2_CO_3_ (93.3 mg, 0.675 mmol, 3.0 equiv), and toluene/water (4:1, 2 mL). Compound **8** was obtained as a yellow-white solid (90 mg, 86%). ^1^H NMR (400 MHz, CDCl_3_): δ 7.53–7.44 (m, 4H), 7.28–7.17 (m, 4H), 6.78 (s, 1H), 4.28–4.14 (m, 4H), 2.78–2.69 (m, 2H), 2.63 (t, *J* = 7.6 Hz, 2H), 2.57–2.49 (m, 2H), 1.98 (s, 3H), 1.71–1.59 (m, 2H), 1.41–1.29 (m, 4H), 1.25 (t, *J* = 7.1 Hz, 6H), 0.95–0.86 (m, 3H). ^13^C NMR (101 MHz, CDCl_3_): δ 169.2, 168.2, 142.1, 139.5, 138.4, 128.98, 128.95, 127.1, 126.9, 66.5, 62.7, 35.7, 33.4, 31.7, 31.3, 29.9, 23.1, 22.7, 14.2, 14.1. HRMS-ESI (*m*/*z*): calcd for C_28_H_38_NO_5_ [M + H]^+^ 468.2750, found: 468.2750.

#### Diethyl (*E*)-2-Acetamido-2-[4-(3-phenylprop-1-en-1-yl)phenethyl]malonate (**9**)

General procedure B was followed using **2** (0.100 g, 0.250 mmol), *trans*-3-phenylpropen-1-yl-boronic acid (48.6 mg, 0.300 mmol, 1.2 equiv), Pd_2_(dba)_3_ (11.4 mg, 0.0125 mmol, 0.05 equiv), SPhos (10.3 mg, 0.0250 mmol, 0.1 equiv), K_2_CO_3_ (0.104 mg, 0.750 mmol, 3.0 equiv), and toluene/water (4:1, 2 mL). Compound **9** was obtained as a yellow-white solid (71 mg, 65%). ^1^H NMR (400 MHz, CDCl_3_): δ 7.35–7.17 (m, 7H), 7.10–7.04 (m, 2H), 6.76 (s, 1H), 6.41 (d, *J* = 15.8 Hz, 1H), 6.35–6.26 (m, 1H), 4.28–4.14 (m, 4H), 3.54 (d, *J* = 6.6 Hz, 2H), 2.72–2.63 (m, 2H), 2.50–2.41 (m, 2H), 1.98 (s, 3H), 1.25 (t, *J* = 7.1 Hz, 6H). ^13^C NMR (101 MHz, CDCl_3_): δ 169.1, 168.1, 140.1, 139.5, 135.5, 130.8, 128.7, 128.6, 128.5, 126.2, 126.1, 66.4, 62.6, 39.4, 33.3, 29.9, 23.0, 14.0. HRMS-ESI (*m*/*z*): calcd for C_26_H_32_NO_5_ [M + H]^+^ 438.2280, found: 438.2282.

#### 2-Amino-2-(4-hexylphenethyl)propane-1,3-diol (**10**)

General procedure D was followed using **3** (63.7 mg, 0.157 mmol), NaBH_4_ (29.7 mg, 0.786 mmol, 5.0 equiv), CaCl_2_ (43.6 mg, 0.393 mmol, 2.5 equiv), and EtOH/water (4:1, 2 mL); N*-*acetylated diol intermediate (white solid, 64 mg), MeOH/THF (2 mL), and 2 M LiOH (aq., 2 mL). Compound **10** was obtained as a white solid (24 mg, 55%). ^1^H NMR (400 MHz, CD_3_OD): δ 7.14–7.09 (m, 2H), 7.08–7.03 (m, 2H), 3.48 (q, *J* = 14.6, 10.9 Hz, 4H), 2.64–2.58 (m, 2H), 2.55 (t, *J* = 7.5 Hz, 2H), 1.70–1.62 (m, 2H), 1.61–1.53 (m, 2H), 1.37–1.26 (m, 6H), 0.93–0.85 (m, 3H). ^13^C NMR (101 MHz, CD_3_OD): δ 141.3, 141.2, 129.4, 129.2, 66.5, 56.8, 37.7, 36.5, 32.9, 32.8, 30.0, 23.7, 14.4. HRMS-ESI (*m*/*z*): calcd for C_17_H_30_NO_2_ [M + H]^+^ 280.2277, found: 280.2277.

#### 2-Amino-2-(4-decylphenethyl)propane-1,3-diol (**11**)

General procedure D was followed using **4** (63.5 mg, 0.138 mmol), NaBH_4_ (26.0 mg, 0.668 mmol, 5.0 equiv), CaCl_2_ (38.2 mg, 0.344 mmol, 2.5 equiv), and EtOH/water (4:1, 1.0 mL); N*-*acetylated diol intermediate (white solid, 51 mg), MeOH/THF (1 mL), and 2 M LiOH (aq., 2 mL). Compound **11** was obtained as a white solid (30 mg, 65%). ^1^H NMR (400 MHz, CD_3_OD): δ 7.14–7.09 (m, 2H), 7.08–7.03 (m, 2H), 3.49 (q, *J* = 14.4, 10.9 Hz, 4H), 2.65–2.58 (m, 2H), 2.55 (t, *J* = 7.4 Hz, 2H), 1.70–1.61 (m, 2H), 1.64–1.53 (m, 2H), 1.37–1.21 (m, 14H), 0.94–0.86 (m, 3H). ^13^C NMR (101 MHz, CD_3_OD): δ 141.3, 141.2, 129.4, 129.2, 66.4, 56.9, 37.7, 36.5, 33.1, 32.8, 30.73, 30.71, 30.6, 30.4, 30.3, 30.0, 23.7, 14.4. HRMS-ESI (*m*/*z*): calcd for C_21_H_38_NO_2_ [M + H]^+^ 336.2903, found: 336.2906.

#### 2-Amino-2-(4-benzylphenethyl)propane-1,3-diol (**12**)

General procedure D was followed using **5** (62.5 mg, 0.152 mmol), NaBH_4_ (28.7 mg, 0.760 mmol, 5.0 equiv), CaCl_2_ (41.9 mg, 0.380 mmol, 2.5 equiv), and EtOH/water (4:1, 2.0 mL); N*-*acetylated diol intermediate (transparent oil, 53.8 mg), MeOH/THF (1 mL), and 2 M LiOH (aq., 2 mL). Compound **12** was obtained as a white solid (21 mg, 49%). ^1^H NMR (400 MHz, CD_3_OD): δ 7.28–7.20 (m, 2H), 7.19–7.10 (m, 5H), 7.10–7.05 (m, 2H), 3.91 (s, 2H), 3.49 (q, *J* = 13.9, 10.9 Hz, 4H), 2.66–2.57 (m, 2H), 1.70–1.61 (m, 2H). ^13^C NMR (101 MHz, CD_3_OD): δ 143.0, 141.7, 140.1, 129.90, 129.85, 129.41, 129.38, 126.9, 66.4, 57.0, 42.4, 37.6, 30.0. HRMS-ESI (*m*/*z*): calcd for C_18_H_24_NO_2_ [M + H]^+^ 286.1807, found: 286.1808.^41^

#### 2-Amino-2-[2-(3′,4′-dimethoxy-[1,1′-biphenyl]-4-yl)ethyl]propane-1,3-diol (**13**)

General procedure D was followed using **6** (96.0 mg, 0.210 mmol), NaBH_4_ (39.7 mg, 1.05 mmol, 5.0 equiv), CaCl_2_ (58.3 mg, 0.525 mmol, 2.5 equiv), and EtOH/water (4:1, 2 mL); N*-*acetylated diol intermediate (white solid, 64 mg), MeOH/THF (2:1, 2 mL), and 2 M LiOH (aq., 2 mL). Compound **13** was obtained as a white solid (38 mg, 54%). ^1^H NMR (400 MHz, CD_3_OD): δ 7.51–7.46 (m, 2H), 7.30–7.25 (m, 2H), 7.17–7.13 (m, 2H), 7.00 (d, *J* = 8.2 Hz, 1H), 3.89 (s, 3H), 3.86 (s, 3H), 3.51 (q, *J* = 13.9, 10.9 Hz, 4H), 2.72–2.65 (m, 2H), 1.74–1.67 (m, 2H). ^13^C NMR (101 MHz, CD_3_OD): δ 150.7, 150.0, 142.8, 139.8, 135.7, 129.8, 127.7, 120.4, 113.4, 111.9, 66.5, 56.9, 56.7, 37.6, 30.0. HRMS-ESI (*m*/*z*): calcd for C_19_H_26_NO_4_ [M + H]^+^ 332.1862, found: 332.1862.

#### 2-Amino-2-[2-[4′-(benzyloxy)-[1,1′-biphenyl]-4-yl]ethyl]propane-1,3-diol (**14**)

General procedure D was followed using **7** (50.0 mg, 0.0993 mmol), NaBH_4_ (18.8 mg, 0.497 mmol, 5.0 equiv), CaCl_2_ (27.5 mg, 0.248 mmol, 2.5 equiv), and EtOH/water (4:1, 1 mL); N*-*acetylated diol intermediate (white solid, 37 mg), MeOH/THF (1 mL), and 2 M LiOH (aq., 2 mL). Compound **14** was obtained as a white solid (23 mg, 60%). ^1^H NMR (400 MHz, DMSO-*d*_6_): δ 7.59–7.53 (m, 2H), 7.53–7.44 (m, 4H), 7.44–7.37 (m, 2H), 7.36–7.30 (m, 1H), 7.26–7.21 (m, 2H), 7.11–7.04 (m, 2H), 5.15 (s, 2H), 4.46 (s, 1H), 3.33–3.18 (m, 5H), 2.65–2.56 (m, 2H), 1.56–1.47 (m, 2H), 1.32 (s, 1H). ^13^C NMR (101 MHz, DMSO-*d*_6_): δ 157.7, 142.0, 137.1, 137.0, 132.8, 128.7, 128.5, 127.8, 127.7, 127.5, 126.0, 115.2, 69.2, 65.4, 55.4, 36.8, 28.6. HRMS-ESI (*m*/*z*): calcd for C_24_H_28_NO_3_ [M + H]^+^ 378.2069, found: 378.2070.

#### 2-Amino-2-[2-(4′-pentyl-[1,1′-biphenyl]-4-yl)ethyl]propane-1,3-diol (**15**)

General procedure D was followed using **8** (86.0 mg, 0.184 mmol), NaBH_4_ (34.8 mg, 0.920 mmol, 5.0 equiv), CaCl_2_ (51.1 mg, 0.460 mmol, 2.5 equiv), and EtOH/water (4:1, 2 mL); N*-*acetylated diol intermediate (white solid, 69 mg), MeOH/THF (2 mL), and 2 M LiOH (aq., 2 mL). Compound **15** was obtained as a white solid (36 mg, 57%). ^1^H NMR (400 MHz, CD_3_OD): δ 7.52–7.46 (m, 4H), 7.31–7.25 (m, 2H), 7.25–7.19 (m, 2H), 3.50 (q, *J* = 14.4, 10.8 Hz, 4H), 2.73–2.66 (m, 2H), 2.63 (t, *J* = 7.3 Hz, 2H), 1.75–1.67 (m, 2H), 1.67–1.59 (m, 2H), 1.43–1.28 (m, 4H), 0.96–0.88 (m, 3H). ^13^C NMR (101 MHz, CD_3_OD): δ 142.94, 142.92, 139.9, 139.8, 129.9, 129.8, 127.8, 127.7, 66.5, 56.9, 37.6, 36.5, 32.6, 32.5, 30.1, 23.6, 14.4. HRMS-ESI (*m*/*z*): calcd for C_22_H_32_NO_2_ [M + H]^+^ 342.2433, found: 342.2434.^42^

#### (*E*)-2-Amino-2-[4-(3-phenylprop-1-en-1-yl)phenethyl]propane-1,3-diol (**16**)

General procedure D was followed using **10** (70.0 mg, 0.160 mmol), NaBH_4_ (30.3 mg, 0.800 mmol, 5.0 equiv), CaCl_2_ (44.4 mg, 0.400 mmol, 2.5 equiv), and EtOH/water (4:1, 2 mL); N*-*acetylated diol intermediate (white solid, 62 mg), MeOH/THF (1 mL), and 2 M LiOH (aq., 2 mL). Compound **16** was obtained as a white solid (34 mg, 67%). ^1^H NMR (400 MHz, CD_3_OD): δ 7.32–7.10 (m, 9H), 6.41 (d, *J* = 15.9 Hz, 1H), 6.35–6.26 (m, 1H), 3.55–3.42 (m, 6H), 2.66–2.57 (m, 2H), 1.70–1.61 (m, 2H). ^13^C NMR (101 MHz, CD_3_OD): δ 143.0, 141.7, 136.6, 132.0, 129.6, 129.53, 129.51, 129.48, 127.2, 127.1, 66.5, 56.8, 40.3, 37.5, 30.1. HRMS-ESI (*m*/*z*): calcd for C_20_H_26_NO_2_ [M + H]^+^ 312.1964, found: 312.1966.

#### 4-(Benzyloxy)phenethyl Methanesulfonate (**18**)

To a cooled solution of 2-[4-(benzyloxy)phenyl]ethanol (**17**) (1.50 g, 6.57 mmol) and triethylamine (Et_3_N; 1.37 mL, 9.86 mmol, 1.5 equiv) in anhydrous DCM (25 mL) was added methanesulfonyl chloride (0.66 mL, 8.54 mmol, 1.3 equiv), and the reaction mixture was stirred for 2 h. The mixture was diluted with DCM and washed with brine. The organic layer was dried over anhydrous Na_2_SO_4_, filtered, and concentrated on a rotary evaporator. Column chromatography, with an increasing gradient of EtOAc in *n*-heptane, starting with 20% of EtOAc, gave compound **18** as a white solid (1.72 g, 86%). ^1^H NMR (400 MHz, CDCl_3_): δ 7.47–7.36 (m, 4H), 7.36–7.30 (m, 1H), 7.18–7.12 (m, 2H), 6.97–6.91 (m, 2H), 5.06 (s, 2H), 4.38 (t, *J* = 6.9 Hz, 2H), 3.00 (t, *J* = 6.9 Hz, 2H), 2.83 (s, 3H). ^13^C NMR (101 MHz, CDCl_3_): δ 158.0, 137.1, 130.2, 128.7, 128.1, 127.6, 115.3, 70.7, 70.2, 37.4, 34.9. HRMS-ESI (*m*/*z*): calcd for C_16_H_18_O_4_S [M + H]^+^ 306.0926, found: 306.0925.

#### Diethyl 2-Acetamido-2-[4-(benzyloxy)phenethyl]malonate (**19**)

General procedure A was followed using **18** (1.70 g, 5.62 mmol, 1.2 equiv), DEAM (1.02 g, 4.68 mmol), Cs_2_CO_3_ (3.05 g, 9.36 mmol, 2.0 equiv), and anhydrous MeCN (20 mL). Compound **19** was obtained as a yellow-white solid (0.97 g, 49%). ^1^H NMR (400 MHz, CDCl_3_): δ 7.44–7.34 (m, 4H), 7.34–7.28 (m, 1H), 7.08–7.03 (m, 2H), 6.91–6.85 (m, 2H), 6.76 (bs, 1H), 5.04 (s, 2H), 4.27–4.12 (m, 4H), 2.70–2.62 (m, 2H), 2.47–2.39 (m, 2H), 1.98 (s, 3H), 1.24 (t, *J* = 7.1 Hz, 6H). ^13^C NMR (101 MHz, CDCl_3_): δ 169.1, 168.2, 157.3, 137.3, 133.0, 129.5, 128.7, 128.0, 127.6, 114.9, 70.1, 66.5, 62.7, 33.6, 29.4, 23.1, 14.1. HRMS-ESI (*m*/*z*): calcd for C_24_H_30_NO_6_ [M + H]^+^ 428.2073, found: 428.2075.

#### Diethyl 2-Acetamido-2-(4-hydroxyphenethyl)malonate (**20**)

To a solution of **19** (0.788 g, 1.84 mmol) in EtOH/EtOAc (1:1, 20 mL) was added 10% palladium on carbon (0.196 g, 0.184 mmol, 0.1 equiv). The reaction mixture was stirred at room temperature under a hydrogen atmosphere for 5 h, after which it was filtered through Celite and concentrated on a rotary evaporator. The resulting solid was washed with diisopropyl ether and dried in a vacuum to give **20** as a white solid (547 mg, 88%). ^1^H NMR (400 MHz, CD_3_OD): δ 7.00–6.94 (m, 2H), 6.73–6.68 (m, 2H), 4.27–4.13 (m, 4H), 2.54–2.48 (m, 2H), 2.45–2.39 (m, 2H), 2.01 (s, 3H), 1.25 (t, *J* = 7.1 Hz, 6H). ^13^C NMR (101 MHz, CD_3_OD): δ 172.5, 169.2, 156.7, 132.9, 130.3, 116.2, 67.9, 63.4, 35.7, 30.2, 22.3, 14.3. HRMS-ESI (*m*/*z*): calcd for C_17_H_24_NO_6_ [M + H]^+^ 338.1604, found: 338.1603.

#### Diethyl 2-Acetamido-2-[4-[[4-(trifluoromethyl)benzyl]oxy]phenethyl]malonate (**21**)

General procedure C was followed using **20** (80.0 mg, 0.237 mmol), K_2_CO_3_ (65.5 mg, 0.474 mmol, 2.0 equiv), 1-(bromomethyl)-4-(trifluoromethyl)benzene (68.0 mg, 0.285 mmol, 1.2 equiv), and anhydrous DMF (2 mL). Compound **21** was obtained as a yellow solid (102 mg, 87%). ^1^H NMR (400 MHz, CDCl_3_): δ 7.67–7.60 (m, 2H), 7.57–7.50 (m, 2H), 7.10–7.02 (m, 2H), 6.90–6.82 (m, 2H), 6.76 (bs, 1H), 5.10 (s, 2H), 4.27–4.13 (m, 4H), 2.70–2.61 (m, 2H), 2.47–2.38 (m, 2H), 1.99 (s, 3H), 1.24 (t, *J* = 7.1 Hz, 6H). ^13^C NMR (101 MHz, CDCl_3_): δ 169.1, 168.20, 168.18, 156.9, 141.4, 133.5, 130.1 (q, *J* = 32.7 Hz), 129.6, 127.5, 125.7 (q, *J* = 4.0 Hz), 114.9, 69.3, 66.5, 66.4, 62.7, 33.6, 29.4, 23.2, 23.1, 14.1. ^19^F NMR (377 MHz, CDCl_3_): δ −64.0. HRMS-ESI (*m*/*z*): calcd for C_25_H_29_NO_6_F_3_ [M + H]^+^ 496.1947, found: 496.1949.

#### Diethyl 2-Acetamido-2-[4-[[6-(trifluoromethyl)pyridin-3-yl]methoxy]phenethyl]malonate (**22**)

General procedure C was followed using **20** (90.0 mg, 0.267 mmol), K_2_CO_3_ (73.8 mg, 0.534 mmol, 2.0 equiv), 5-(bromomethyl)-2-(trifluoromethyl)pyridine (76.8 mg, 0.320 mmol, 1.2 equiv), and anhydrous DMF (2 mL). Compound **22** was obtained as a white solid (116 mg, 88%). ^1^H NMR (400 MHz, CDCl_3_): δ 8.77 (d, *J* = 1.8 Hz, 1H), 7.98–7.93 (m, 1H), 7.71 (d, *J* = 7.7 Hz, 1H), 7.11–7.05 (m, 2H), 6.90–6.84 (m, 2H), 6.77 (bs, 1H), 5.13 (s, 2H), 4.28–4.13 (m, 4H), 2.70–2.61 (m, 2H), 2.47–2.38 (m, 2H), 2.00 (s, 3H), 1.25 (t, *J* = 7.1 Hz, 6H). ^13^C NMR (101 MHz, CDCl_3_): δ 169.2, 168.2, 156.5, 149.0, 136.4, 134.0, 129.7, 120.5 (d, *J* = 2.9 Hz), 114.9, 67.0, 66.5, 62.7, 33.7, 29.4, 23.1, 14.1. ^19^F NMR (377 MHz, CDCl_3_): δ −67.9. HRMS-ESI (*m*/*z*): calcd for C_24_H_28_N_2_O_6_F_3_ [M + H]^+^ 497.1899, found: 497.1896.

#### Diethyl 2-Acetamido-2-[4-[(7-chloroquinolin-2-yl)methoxy]phenethyl]malonate (**23**)

General procedure C was followed using **20** (80.0 mg, 0.237 mmol), K_2_CO_3_ (65.5 mg, 0.474 mmol, 2.0 equiv), 2-(bromomethyl)-7-chloroquinoline (72.9 mg, 0.284 mmol, 1.2 equiv), and anhydrous DMF (2 mL). Compound **23** was obtained as a white solid (118 mg, 97%). ^1^H NMR (400 MHz, CDCl_3_): δ 8.15 (d, *J* = 8.5 Hz, 1H), 8.08 (d, *J* = 2.0 Hz, 1H), 7.75 (d, *J* = 8.7 Hz, 1H), 7.65 (d, *J* = 8.5 Hz, 1H), 7.50 (dd, *J* = 8.7, 2.1 Hz, 1H), 7.08–7.02 (m, 2H), 6.94–6.88 (m, 2H), 6.75 (bs, 1H), 5.34 (s, 2H), 4.26–4.11 (m, 4H), 2.68–2.59 (m, 2H), 2.45–2.37 (m, 2H), 1.97 (s, 3H), 1.23 (t, *J* = 7.1 Hz, 6H). ^13^C NMR (101 MHz, CDCl_3_): δ 169.1, 168.2, 159.4, 156.8, 137.0, 135.8, 133.5, 129.7, 129.0, 128.1, 127.8, 126.1, 119.4, 114.9, 71.1, 66.5, 62.7, 33.6, 29.4, 23.1, 14.1. HRMS-ESI (*m*/*z*): calcd for C_27_H_30_N_2_O_6_Cl [M + H]^+^ 513.1792, found: 513.1794.

#### Diethyl 2-Acetamido-2-[4-[(4-butylbenzyl)oxy]phenethyl]malonate (**24**)

General procedure C was followed using **20** (70.0 mg, 0.207 mmol), K_2_CO_3_ (57.2 mg, 0.414 mmol, 2.0 equiv), 4-butylbenzyl bromide (56.4 mg, 0.248 mmol, 1.2 equiv), and anhydrous DMF (2 mL). Compound **24** was obtained as a white solid (54 mg, 54%). ^1^H NMR (400 MHz, CDCl_3_): δ 7.35–7.30 (m, 2H), 7.22–7.16 (m, 2H), 7.08–7.02 (m, 2H), 6.91–6.85 (m, 2H), 6.75 (bs, 1H), 4.99 (s, 2H), 4.26–4.11 (m, 4H), 2.70–2.57 (m, 4H), 2.47–2.38 (m, 2H), 1.98 (s, 3H), 1.65–1.53 (m, 2H), 1.41–1.30 (m, 2H), 1.24 (t, *J* = 7.1 Hz, 6H), 0.92 (t, *J* = 7.3 Hz, 3H). ^13^C NMR (101 MHz, CDCl_3_): δ 169.1, 168.2, 157.4, 142.9, 134.4, 132.9, 129.5, 128.8, 127.7, 114.9, 70.1, 66.5, 62.7, 35.5, 33.7, 33.6, 29.4, 23.1, 22.5, 14.12, 14.07. HRMS-ESI (*m*/*z*): calcd for C_28_H_38_NO_6_ [M + H]^+^: 484.2699, found: 484.2700.

#### 2-Amino-2-[4-(benzyloxy)phenethyl]propane-1,3-diol (**25**)

Modified general procedure D was followed using **19** (45.0 mg, 0.105 mmol), LiBH_4_ (2 M solution in THF, 0.16 mL, 0.315 mmol, 3.0 equiv), and THF (1 mL); N-acetylated diol intermediate (transparent oil, 34 mg), MeOH/THF (1 mL), and 2 M LiOH (aq., 1 mL). Compound **25** was obtained as a white solid (12 mg, 38%). ^1^H NMR (400 MHz, CD_3_OD): δ 7.44–7.39 (m, 2H), 7.38–7.32 (m, 2H), 7.31–7.26 (m, 1H), 7.16–7.10 (m, 2H), 6.91–6.86 (m, 2H), 5.03 (s, 2H), 3.48 (q, *J* = 14.5, 10.9 Hz, 4H), 2.63–2.54 (m, 2H), 1.68–1.59 (m, 2H). ^13^C NMR (101 MHz, CD_3_OD): δ 158.4, 139.0, 136.4, 130.2, 129.5, 128.8, 128.5, 115.9, 71.0, 66.5, 56.8, 37.8, 29.5. HRMS-ESI (*m*/*z*): calcd for C_18_H_24_NO_3_ [M + H]^+^ 302.1756, found: 302.1754.

#### 2-Amino-2-[4-[[4-(trifluoromethyl)benzyl]oxy]phenethyl]propane-1,3-diol (**26**)

General procedure D was followed using **21** (91.0 mg, 0.184 mmol), NaBH_4_ (34.7 mg, 0.919 mmol, 5.0 equiv), CaCl_2_ (50.9 mg, 0.459 mmol, 2.5 equiv), and EtOH/water (4:1, 2 mL); N-acetylated diol intermediate (white solid, 77 mg), MeOH/THF (1 mL), and 2 M LiOH (aq., 2 mL). Compound **26** was obtained as a white solid (50 mg, 73%). ^1^H NMR (400 MHz, CD_3_OD): δ 7.64 (q, *J* = 10.7, 8.3 Hz, 4H), 7.19–7.10 (m, 2H), 6.95–6.86 (m, 2H), 5.14 (s, 2H), 3.50 (q, *J* = 12.3, 10.9 Hz, 4H), 2.64–2.55 (m, 2H), 1.72–1.63 (m, 2H). ^13^C NMR (101 MHz, CD_3_OD): δ 158.1, 143.7, 136.7, 130.5 (q, *J* = 32.7, 32.0 Hz), 130.4, 128.7, 127.1, 126.4 (q, *J* = 3.7 Hz), 124.4, 115.9, 70.1, 66.5, 37.8, 29.5. ^19^F NMR (377 MHz, CD_3_OD): δ −64.0. HRMS-ESI (*m*/*z*): calcd for C_19_H_23_NO_3_F_3_ [M + H]^+^ 370.1630, found: 370.1631.

#### 2-Amino-2-[4-[[5-(trifluoromethyl)pyridin-2-yl]methoxy]phenethyl]propane-1,3-diol (**27**)

Modified general procedure D was followed using **22** (0.101 g, 0.203 mmol), LiBH_4_ (2 M solution in THF, 0.30 mL, 0.609 mmol, 3.0 equiv), and THF (1 mL); N-acetylated diol intermediate (white solid, 73 mg), MeOH/THF (1.5 mL), and 2 M LiOH (aq., 1.5 mL). Compound **27** was obtained as a white solid (26 mg, 34%). ^1^H NMR (400 MHz, CD_3_OD): δ 8.78 (d, *J* = 2.1 Hz, 1H), 8.14–8.08 (m, 1H), 7.83 (d, *J* = 8.1 Hz, 1H), 7.20–7.13 (m, 2H), 6.97–6.90 (m, 2H), 5.22 (s, 2H), 3.48 (q, *J* = 14.0, 11.0 Hz, 4H), 2.65–2.56 (m, 2H), 1.69–1.60 (m, 2H). ^13^C NMR (101 MHz, CD_3_OD): δ 157.8, 150.0, 148.7, 148.3, 148.0, 138.8, 138.2, 137.1, 130.5, 124.4, 121.69, 121.66, 121.63, 121.60, 115.9, 67.8, 66.4, 56.9, 37.7, 29.5. ^19^F NMR (377 MHz, CD_3_OD): δ −69.3. HRMS-ESI (*m*/*z*): calcd for C_18_H_22_N_2_O_3_F_3_ [M + H]^+^ 371.1583, found: 371.1582.

#### 2-Amino-2-[4-[(7-chloroquinolin-2-yl)methoxy]phenethyl]propane-1,3-diol (**28**)

General procedure D was followed using **23** (99.0 mg, 0.193 mmol), NaBH_4_ (36.5 mg, 0.965 mmol, 5.0 equiv), CaCl_2_ (53.6 mg, 0.483 mmol, 2.5 equiv), and EtOH/water (4:1, 2 mL); N-acetylated diol intermediate (white solid, 80 mg), MeOH/THF (1:3, 2 mL), and 2 M LiOH (aq., 2 mL). Compound **28** was obtained as a white solid (49 mg, 66%). ^1^H NMR (400 MHz, DMSO-*d*_6_): δ 8.46 (d, *J* = 8.6 Hz, 1H), 8.09–8.03 (m, 2H), 7.69 (d, *J* = 8.5 Hz, 1H), 7.66 (dd, *J* = 8.7, 2.2 Hz, 1H), 7.13–7.07 (m, 2H), 6.98–6.92 (m, 2H), 5.33 (s, 2H), 4.41 (bs, 2H), 3.28–3.15 (m, 4H), 1.50–1.41 (m, 2H), 1.26 (bs, 1H) (one peak covered by DMSO). ^13^C NMR (101 MHz, DMSO-*d*_6_): δ 159.4, 156.0, 147.3, 137.1, 136.0, 134.4, 130.0, 129.2, 127.2, 125.8, 119.9, 114.6, 70.6, 65.4, 55.4, 37.0, 28.0. HRMS-ESI (*m*/*z*): calcd for C_21_H_24_N_2_O_3_Cl [M + H]^+^ 387.1475, found: 387.1474.

#### 2-Amino-2-[4-[(4-butylbenzyl)oxy]phenethyl]propane-1,3-diol (**29**)

General procedure D was followed using **24** (53.0 mg, 0.110 mmol), NaBH_4_ (20.8 mg, 0.550 mmol, 5.0 equiv), CaCl_2_ (30.5 mg, 0.275 mmol, 2.5 equiv), and EtOH/water (4:1, 1.5 mL); N-acetylated diol intermediate (white solid, 47 mg), MeOH/THF (2 mL), and 2 M LiOH (aq., 2 mL). Compound **29** was obtained as a white solid (26 mg, 66%). ^1^H NMR (400 MHz, CD_3_OD): δ 7.35–7.28 (m, 2H), 7.21–7.15 (m, 2H), 7.15–7.09 (m, 2H), 6.91–6.84 (m, 2H), 4.99 (s, 2H), 3.48 (q, *J* = 14.5, 10.7 Hz, 4H), 2.65–2.54 (m, 4H), 1.68–1.54 (m, 4H), 1.41–1.30 (m, 2H), 0.94 (t, *J* = 7.4 Hz, 3H). ^13^C NMR (101 MHz, CD_3_OD): δ 158.5, 143.7, 136.3, 136.1, 130.2, 129.5, 128.7, 115.9, 71.0, 66.5, 56.8, 37.8, 36.3, 35.0, 29.5, 23.3, 14.5. HRMS-ESI (*m*/*z*): calcd for C_22_H_32_NO_3_ [M + H]^+^ 358.2382, found: 358.2383.

#### 2-(Quinolin-2-yl)ethyl Methanesulfonate (**31**)

To a cooled solution of 2-(quinolin-2-yl)ethan-1-ol (**30**) (0.150 g, 0.866 mmol) and Et_3_N (0.247 mL, 1.77 mmol, 2.0 equiv) in anhydrous DCM (4 mL) was added methanesulfonyl chloride (0.122 mL, 1.06 mmol, 1.2 equiv), and the reaction mixture was stirred for 3 h. The mixture was diluted with DCM and washed with water and brine. The organic layer was dried over anhydrous Na_2_SO_4_, filtered, and concentrated on a rotary evaporator. Column chromatography, with an increasing gradient of EtOAc in *n*-heptane, starting with 20% of EtOAc, gave **31** as a white solid (78 mg, 37%). ^1^H NMR (400 MHz, CDCl_3_): δ 8.12 (d, *J* = 8.4 Hz, 1H), 8.01 (d, *J* = 8.5 Hz, 1H), 7.81 (d, *J* = 8.1 Hz, 1H), 7.74–7.68 (m, 1H), 7.57–7.48 (m, 1H), 7.34 (d, *J* = 8.4 Hz, 1H), 4.81 (t, *J* = 6.4 Hz, 2H), 3.42 (t, *J* = 6.4 Hz, 2H), 2.93 (s, 3H). ^13^C NMR (101 MHz, CDCl_3_): δ 157.2, 148.0, 136.8, 129.9, 128.9, 127.8, 127.2, 126.5, 122.0, 69.0, 38.1, 37.4.

#### Diethyl 2-Acetamido-2-[2-(quinolin-2-yl)ethyl]malonate (**32**)

General procedure A was followed using **31** (76.6 mg, 0.305 mmol, 1.2 equiv), DEAM (55.0 mg, 0.254 mmol), Cs_2_CO_3_ (166 mg, 0.508 mmol, 2.0 equiv), and anhydrous MeCN (2 mL). Compound **32** was obtained as a yellow oil (50 mg, 53%). ^1^H NMR (400 MHz, CDCl_3_): δ 8.06 (d, *J* = 8.6 Hz, 1H), 8.00 (d, *J* = 8.4 Hz, 1H), 7.76 (d, *J* = 8.2 Hz, 1H), 7.70–7.64 (m, 1H), 7.51–7.45 (m, 1H), 7.28 (d, *J* = 8.5 Hz, 1H), 6.98 (s, 1H), 4.29–4.10 (m, 4H), 2.89 (s, 4H), 1.97 (s, 3H), 1.24 (t, *J* = 7.1 Hz, 6H). ^13^C NMR (101 MHz, CDCl_3_): δ 169.3, 168.2, 161.1, 147.9, 136.5, 129.6, 128.9, 127.6, 126.9, 126.1, 121.3, 66.4, 62.7, 33.6, 32.0, 23.1, 14.1. HRMS-ESI (*m*/*z*): calcd for C_20_H_25_N_2_O_5_ [M + H]^+^ 373.1763, found: 373.1763.

#### 2-Amino-2-[2-(quinolin-2-yl)ethyl]propane-1,3-diol (**33**)

General procedure D was followed using **32** (48.0 mg, 0.129 mmol), NaBH_4_ (24.0 mg, 0.645 mmol, 5.0 equiv), CaCl_2_ (36.0 mg, 0.322 mmol, 2.5 equiv), and EtOH/water (4:1, 1 mL); N-acetylated diol intermediate (yellow oil, 33 mg), MeOH/THF (1 mL), and 2 M LiOH (aq., 2 mL). Compound **33** was obtained as a white solid (0.010 g, 31%). ^1^H NMR (400 MHz, CDCl_3_): δ 8.07 (d, *J* = 8.5 Hz, 1H), 7.99 (d, *J* = 8.5 Hz, 1H), 7.77 (d, *J* = 8.1 Hz, 1H), 7.72–7.64 (m, 1H), 7.53–7.46 (m, 1H), 7.29 (d, *J* = 8.5 Hz, 1H), 3.55 (q, *J* = 11.2 Hz, 4H), 3.19 (bs, 4H), 3.10 (t, *J* = 6.9 Hz, 2H), 2.05 (t, *J* = 6.9 Hz, 2H). ^13^C NMR (101 MHz, CDCl_3_): δ 162.6, 147.3, 137.0, 130.0, 128.16, 127.7, 126.9, 126.3, 122.0, 66.8, 56.7, 32.5, 31.8. HRMS-ESI (*m*/*z*): calcd for C_14_H_19_N_2_O_2_ [M + H]^+^ 247.1447, found: 247.1445.

#### Diethyl 2-Acetamido-2-(3-bromophenethyl)malonate (**36**)

General procedure A was followed using 1-bromo-3-(2-bromoethyl)benzene (**34**) (0.500 g, 1.89 mmol, 1.4 equiv), DEAM (0.294 g, 1.35 mmol), Cs_2_CO_3_ (0.661 g, 2.03 mmol, 1.5 equiv), and anhydrous MeCN (10 mL). Compound **36** was obtained as a yellow solid (283 mg, 52%). ^1^H NMR (400 MHz, CDCl_3_): δ 7.34–7.28 (m, 2H), 7.16–7.10 (m, 1H), 7.09–7.05 (m, 1H), 6.76 (bs, 1H), 4.29–4.14 (m, 4H), 2.72–2.63 (m, 2H), 2.50–2.41 (m, 2H), 2.01 (s, 3H), 1.25 (t, *J* = 7.1 Hz, 6H). ^13^C NMR (101 MHz, CDCl_3_): δ 169.3, 168.1, 143.1, 131.6, 130.1, 129.4, 127.3, 122.5, 66.4, 62.82, 33.3, 30.0, 23.1, 14.1. HRMS-ESI (*m*/*z*): calcd for C_17_H_23_NO_5_Br [M + H]^+^ 400.0760, found: 400.0763.

#### Diethyl 2-Acetamido-2-(4-bromobenzyl)malonate (**37**)

General procedure A was followed using 1-bromo-4-(bromomethyl)benzene (**35**) (1.50 g, 6.00 mmol, 1.4 equiv), DEAM (0.931 g, 4.29 mmol), Cs_2_CO_3_ (2.10 g, 6.43 mmol, 1.5 equiv), and anhydrous MeCN (15 mL). Compound **37** was obtained as a white solid (1.49 g, 90%). ^1^H NMR (400 MHz, CDCl_3_): δ 7.41–7.35 (m, 2H), 6.91–6.85 (m, 2H), 6.52 (bs, 1H), 4.34–4.18 (m, 4H), 3.61 (s, 2H), 2.03 (s, 3H), 1.29 (t, *J* = 7.1 Hz, 6H). ^13^C NMR (101 MHz, CDCl_3_): δ 169.3, 167.5, 134.5, 131.7, 131.6, 121.5, 67.1, 62.9, 37.4, 23.2, 14.2. HRMS-ESI (*m*/*z*): calcd for C_16_H_21_NO_5_Br [M + H]^+^ 386.0603, found: 386.0606.

#### Diethyl (*E*)-2-Acetamido-2-[4-(oct-1-en-1-yl)phenethyl]malonate (**38**)

General procedure B was followed using **2** (0.100 g, 0.250 mmol), *trans*-1-octen-1-yl-boronic acid (46.9 mg, 0.300 mmol, 1.2 equiv), Pd_2_(dba)_3_ (11.4 mg, 0.0125 mmol, 0.05 equiv), SPhos (10.3 mg, 0.0250 mmol, 0.1 equiv), K_2_CO_3_ (0.104 g, 0.750 mmol, 3.0 equiv), and toluene/water (4:1, 2 mL). Compound **38** was obtained as a yellow solid (84 mg, 78%). ^1^H NMR (400 MHz, CDCl_3_): δ 7.26–7.21 (m, 2H), 7.08–7.04 (m, 2H), 6.76 (s, 1H), 6.32 (d, *J* = 15.6 Hz, 1H), 6.21–6.12 (m, 1H), 4.27–4.13 (m, 4H), 2.71–2.63 (m, 2H), 2.50–2.41 (m, 2H), 2.22–2.13 (m, 2H), 1.98 (s, 3H), 1.51–1.40 (m, 2H), 1.37–1.20 (m, 12H), 0.92–0.83 (m, 3H). ^13^C NMR (101 MHz, CDCl_3_): δ 169.2, 168.2, 139.2, 136.1, 130.8, 129.4, 128.7, 126.0, 66.5, 62.7, 33.4, 33.2, 31.9, 29.9, 29.5, 29.0, 23.1, 22.8, 14.2, 14.1. HRMS-ESI (*m*/*z*): calcd for C_25_H_38_NO_5_ [M + H]^+^ 432.2750, found: 432.2747.

#### Diethyl 2-Acetamido-2-(3-octylphenethyl)malonate (**39**)

General procedure B was followed using **36** (0.100 g, 0.250 mmol), *n*-octylboronic acid (47.4 mg, 0.300 mmol, 1.2 equiv), Pd_2_(dba)_3_ (11.4 mg, 0.0125 mmol, 0.05 equiv), SPhos (10.3 mg, 0.0250 mmol, 0.1 equiv), K_2_CO_3_ (0.104 g, 0.750 mmol, 3.0 equiv), and toluene/water (4:1, 2 mL). Compound **39** was obtained as a white solid (81 mg, 75%). ^1^H NMR (400 MHz, CDCl_3_): δ 7.20–7.13 (m, 1H), 7.02–6.97 (m, 1H), 6.97–6.92 (m, 2H), 6.76 (s, 1H), 4.30–4.13 (m, 4H), 2.72–2.64 (m, 2H), 2.55 (t, *J* = 7.7 Hz, 2H), 2.49–2.41 (m, 2H), 1.98 (s, 3H), 1.64–1.52 (m, 2H), 1.36–1.21 (m, 16H), 0.92–0.84 (m, 3H). ^13^C NMR (101 MHz, CDCl_3_): δ 169.2, 168.2, 143.3, 140.6, 128.7, 128.4, 126.3, 125.8, 66.6, 62.7, 36.1, 33.5, 32.0, 31.8, 30.2, 29.6, 29.6, 29.4, 23.1, 22.8, 14.2, 14.1. HRMS-ESI (*m*/*z*): calcd for C_25_H_40_NO_5_ [M + H]^+^ 434.2906, found: 434.2909.

#### Diethyl 2-Acetamido-2-(4-octylbenzyl)malonate (**40**)

General procedure B was followed using **37** (0.100 g, 0.259 mmol), *n*-octylboronic acid (49.1 mg, 0.311 mmol, 1.2 equiv), Pd_2_(dba)_3_ (11.8 mg, 0.0129 mmol, 0.05 equiv), SPhos (10.6 mg, 0.0259 mmol, 0.1 equiv), K_2_CO_3_ (0.107 g, 0.777 mmol, 3.0 equiv), and toluene/water (4:1, 2 mL). Compound **40** was obtained as a white solid (73 mg, 68%). ^1^H NMR (400 MHz, CDCl_3_): δ 7.10–7.02 (m, 2H), 6.94–6.86 (m, 2H), 6.52 (s, 1H), 4.33–4.20 (m, 4H), 3.60 (s, 2H), 2.55 (t, *J* = 7.7 Hz, 2H), 2.03 (s, 3H), 1.63–1.52 (m, 2H), 1.37–1.20 (m, 16H), 0.92–0.84 (m, 3H). ^13^C NMR (101 MHz, CDCl_3_): δ 169.1, 167.8, 142.0, 132.4, 129.9, 128.5, 67.4, 62.7, 37.0, 35.7, 32.0, 31.5, 29.6, 29.5, 29.4, 23.2, 22.8, 14.3, 14.2. HRMS-ESI (*m*/*z*): calcd for C_24_H_38_NO_5_ [M + H]^+^ 420.2750, found: 420.2754.

#### Diethyl 2-Acetamido-2-(4-decylbenzyl)malonate (**41**)

General procedure B was followed using **37** (0.200 g, 0.518 mmol), *n*-decylboronic acid (0.116 g, 0.621 mmol, 1.2 equiv), Pd_2_(dba)_3_ (23.6 mg, 0.0258 mmol, 0.05 equiv), SPhos (21.2 mg, 0.0518 mmol, 0.1 equiv), K_2_CO_3_ (0.215 g, 1.55 mmol, 3.0 equiv), and toluene/water (4:1, 4 mL). Compound **41** was obtained as a white solid (136 mg, 59%). ^1^H NMR (400 MHz, CDCl_3_): δ 7.09–7.03 (m, 2H), 6.93–6.87 (m, 2H), 6.52 (s, 1H), 4.32–4.21 (m, 4H), 3.60 (s, 2H), 2.54 (t, *J* = 7.9 Hz, 2H), 2.03 (s, 3H), 1.57 (p, *J* = 7.3, 6.7 Hz, 2H), 1.35–1.21 (m, 20H), 0.88 (t, *J* = 6.8 Hz, 3H). ^13^C NMR (101 MHz, CDCl_3_): δ 169.1, 167.8, 142.0, 132.4, 129.9, 128.5, 67.4, 62.7, 37.6, 35.7, 32.1, 31.5, 29.8, 29.7, 29.6, 29.54, 29.48, 23.2, 22.8, 14.36, 14.12. HRMS-ESI (*m*/*z*): calcd for C_26_H_42_NO_5_ [M + H]^+^ 448.3063, found: 448.3065.

#### (*E*)-2-Amino-2-[4-(oct-1-en-1-yl)phenethyl]propane-1,3-diol (**42**)

General procedure D was followed using **38** (83.0 mg, 0.192 mmol), NaBH_4_ (36.3 mg, 0.960 mmol, 5.0 equiv), CaCl_2_ (53.3 mg, 0.480 mmol, 2.5 equiv), and EtOH/water (4:1, 2 mL); N-acetylated diol intermediate (white solid, 69 mg), MeOH/THF 1:1 (1 mL), and 2 M LiOH (aq.) (2 mL). Compound **42** was obtained as a white solid (42 mg, 72%). ^1^H NMR (400 MHz, CD_3_OD): δ 7.27–7.20 (m, 2H), 7.16–7.11 (m, 2H), 6.33 (d, *J* = 15.9 Hz, 1H), 6.24–6.12 (m, 1H), 3.48 (q, *J* = 14.3, 10.9 Hz, 4H), 2.67–2.57 (m, 2H), 2.24–2.14 (m, 2H), 1.70–1.61 (m, 2H), 1.46 (td, *J* = 7.7, 4.5 Hz, 2H), 1.41–1.24 (m, 6H), 0.95–0.88 (m, 3H). ^13^C NMR (101 MHz, CD_3_OD): δ 142.7, 136.9, 131.0, 130.9, 129.5, 127.0, 66.4, 56.9, 37.5, 34.1, 32.9, 30.6, 30.09, 30.06, 23.7, 14.4. HRMS-ESI (*m*/*z*): calcd for C_19_H_32_NO_2_ [M + H]^+^ 306.2433, found: 306.2437.

#### 2-Amino-2-(3-octylphenethyl)propane-1,3-diol (**43**)

General procedure D was followed using **39** (78.2 mg, 0.180 mmol), NaBH_4_ (34.1 mg, 0.902 mmol, 5.0 equiv), CaCl_2_ (50.1 mg, 0.451 mmol, 2.5 equiv), and EtOH/water (4:1, 2 mL); N-acetylated diol intermediate (transparent oil, 60 mg), MeOH/THF (1 mL), and 2 M LiOH (aq., 2 mL). Compound **43** was obtained as a white solid (40 mg, 71%). ^1^H NMR (400 MHz, CD_3_OD): δ 7.14 (t, *J* = 7.5 Hz, 1H), 7.06–6.99 (m, 2H), 6.96 (d, *J* = 7.5 Hz, 1H), 3.49 (q, *J* = 14.5, 10.8 Hz, 4H), 2.66–2.59 (m, 2H), 2.56 (t, *J* = 7.5 Hz, 2H), 1.70–1.63 (m, 2H), 1.63–1.55 (m, 2H), 1.38–1.22 (m, 10H), 0.94–0.85 (m, 3H). ^13^C NMR (101 MHz, CD_3_OD): δ 144.1, 144.0, 129.5, 129.3, 126.8, 126.7, 66.5, 56.8, 37.7, 36.9, 33.0, 32.8, 30.6, 30.41, 30.38, 23.7, 14.4. HRMS-ESI (*m*/*z*): calcd for C_19_H_34_NO_2_ [M + H]^+^ 308.2590, found: 308.2588.^43^

#### 2-Amino-2-(4-octylbenzyl)propane-1,3-diol (**44**)

General procedure D was followed using **40** (67.9 mg, 0.162 mmol), NaBH_4_ (30.6 mg, 0.809 mmol, 5.0 equiv), CaCl_2_ (44.9 mg, 0.405 mmol, 2.5 equiv), and EtOH/water (4:1, 1 mL); N-acetylated diol intermediate (white solid, 54 mg), MeOH/THF (1 mL), and 2 M LiOH (aq., 2 mL). Compound **44** was obtained as a white solid (28 mg, 60%). ^1^H NMR (400 MHz, CD_3_OD): δ 7.18–7.14 (m, 2H), 7.13–7.08 (m, 2H), 3.38 (q, *J* = 10.8, 1.9 Hz, 4H), 2.68 (s, 2H), 2.57 (t, *J* = 8.6, 6.8 Hz, 2H), 1.59 (p, *J* = 7.2 Hz, 2H), 1.38–1.22 (m, 10H), 0.93–0.85 (m, 3H). ^13^C NMR (101 MHz, CD_3_OD): δ 142.2, 135.2, 131.6, 129.3, 66.0, 57.3, 40.1, 36.5, 33.0, 32.8, 30.6, 30.40, 30.37, 23.7, 14.4. HRMS-ESI (*m*/*z*): calcd for C_18_H_32_NO_2_ [M + H]^+^ 294.2433, found: 294.2435.

#### 2-Amino-2-(4-decylbenzyl)propane-1,3-diol (**45**)

General procedure D was followed using **41** (125 mg, 0.279 mmol), NaBH_4_ (52.8 mg, 1.40 mmol, 5.0 equiv), CaCl_2_ (77.5 mg, 0.698 mmol, 2.5 equiv), and EtOH/water (4:1, 2 mL); N-acetylated diol intermediate (white solid, 91 mg), MeOH/THF (2 mL), and 2 M LiOH (aq., 3 mL). Compound **45** was obtained as a white solid (53 mg, 59%). ^1^H NMR (400 MHz, CD_3_OD): δ 7.18–7.13 (m, 2H), 7.13–7.08 (m, 2H), 3.38 (q, *J* = 10.7, 1.9 Hz, 4H), 2.68 (s, 2H), 2.57 (t, *J* = 7.7 Hz, 2H), 1.59 (p, *J* = 7.1 Hz, 2H), 1.30 (d, *J* = 15.7 Hz, 14H), 0.95–0.84 (m, 3H). ^13^C NMR (101 MHz, CD_3_OD): δ 142.2, 135.2, 131.6, 129.3, 66.0, 57.2, 40.1, 36.5, 33.1, 32.8, 30.7, 30.6, 30.5, 30.4, 23.7, 14.4. HRMS-ESI (*m*/*z*): calcd for C_20_H_36_NO_2_ [M + H]^+^ 322.2746, found: 322.2748.

#### Diethyl 2-Acetamido-2-[4-(octyloxy)phenethyl]malonate (**46**)

General procedure C was followed using **20** (0.100 g, 0.296 mmol), K_2_CO_3_ (81.9 mg, 0.593 mmol, 2.0 equiv), 1-bromooctane (0.115 g, 0.593 mmol, 2.0 equiv), and anhydrous DMF (2 mL). Compound **46** was obtained as a white solid (0.101 g, 76%). ^1^H NMR (400 MHz, CDCl_3_): δ 7.07–7.00 (m, 2H), 6.82–6.77 (m, 2H), 6.76 (bs, 1H), 4.27–4.14 (m, 4H), 3.91 (t, *J* = 6.6 Hz, 2H), 2.70–2.61 (m, 2H), 2.46–2.37 (m, 2H), 1.99 (s, 3H), 1.81–1.70 (m, 2H), 1.49–1.38 (m, 2H), 1.37–1.21 (m, 14H), 0.92–0.84 (m, 3H). ^13^C NMR (101 MHz, CDCl_3_): δ 169.1, 168.2, 157.7, 132.5, 129.4, 114.6, 68.2, 66.5, 62.3, 33.7, 31.9, 29.5, 29.43, 29.37, 26.2, 23.2, 22.8, 14.2, 14.1. HRMS-ESI (*m*/*z*): calcd for C_25_H_40_NO_6_ [M + H]^+^ 450.2856, found: 450.2856.

#### Diethyl 2-Acetamido-2-[4-(decyloxy)phenethyl]malonate (**47**)

General procedure C was followed using **20** (0.100 g, 0.296 mmol), K_2_CO_3_ (81.9 mg, 0.593 mmol, 2.0 equiv), 1-bromodecane (0.131 g, 0.593 mmol, 2.0 equiv), and anhydrous DMF (2 mL). Compound **47** was obtained as a white solid (0.110 g, 78%). ^1^H NMR (400 MHz, CDCl_3_): δ 7.06–7.01 (m, 2H), 6.82–6.77 (m, 2H), 6.76 (bs, 1H), 4.28–4.13 (m, 4H), 3.91 (t, *J* = 6.6 Hz, 2H), 2.70–2.61 (m, 2H), 2.46–2.37 (m, 2H), 1.99 (s, 3H), 1.81–1.69 (m, 2H), 1.49–1.37 (m, 2H), 1.36–1.21 (m, 18H), 0.92–0.84 (m, 3H). ^13^C NMR (101 MHz, CDCl_3_): δ 169.1, 168.2, 157.7, 132.5, 129.4, 114.6, 68.2, 66.5, 62.7, 33.7, 32.0, 29.72, 29.70, 29.54, 29.46, 29.44, 29.36, 26.2, 23.2, 22.8, 14.3, 14.1. HRMS-ESI (*m*/*z*): calcd for C_27_H_44_NO_6_ [M + H]^+^ 478.3169, found: 478.3170.

#### 2-Amino-2-[4-(octyloxy)phenethyl]propane-1,3-diol (**48**)

General procedure D was followed using **46** (95.1 mg, 0.212 mmol), NaBH_4_ (40.0 mg, 1.06 mmol, 5.0 equiv), CaCl_2_ (58.7 mg, 0.529 mmol, 2.5 equiv), and EtOH/water (4:1, 2 mL); N-acetylated diol intermediate (white solid, 76 mg), MeOH/THF (1 mL), and 2 M LiOH (aq., 2 mL). Compound **48** was obtained as a white solid (50 mg, 73%). ^1^H NMR (400 MHz, CD_3_OD): δ 7.14–7.08 (m, 2H), 6.83–6.77 (m, 2H), 3.92 (t, *J* = 6.5 Hz, 2H), 3.48 (q, *J* = 14.8, 10.7 Hz, 4H), 2.62–2.54 (m, 2H), 1.80–1.68 (m, 2H), 1.68–1.59 (m, 2H), 1.51–1.42 (m, 2H), 1.41–1.24 (m, 8H), 0.95–0.87 (m, 3H). ^13^C NMR (101 MHz, CD_3_OD): δ 158.7, 135.9, 130.2, 115.5, 69.0, 66.5, 56.8, 37.8, 33.0, 30.51, 30.48, 30.4, 29.5, 27.2, 23.7, 14.4. HRMS-ESI (*m*/*z*): calcd for C_19_H_34_NO_3_ [M + H]^+^ 324.2539, found: 324.2542.^44^

#### 2-Amino-2-[4-(decyloxy)phenethyl]propane-1,3-diol (**49**)

General procedure D was followed using **47** (0.104 g, 0.218 mmol), NaBH_4_ (41.2 mg, 1.09 mmol, 5.0 equiv), CaCl_2_ (60.4 mg, 0.544 mmol, 2.5 equiv), and EtOH/water (4:1, 2 mL); N-acetylated diol intermediate (white solid, 87 mg), MeOH/THF (1 mL), and 2 M LiOH (aq., 2 mL). Compound **49** was obtained as a white solid (54 mg, 71%). ^1^H NMR (400 MHz, CD_3_OD): δ 7.14–7.08 (m, 2H), 6.83–6.76 (m, 2H), 3.92 (t, *J* = 6.5 Hz, 2H), 3.48 (q, *J* = 14.6, 10.9 Hz, 4H), 2.62–2.54 (m, 2H), 1.80–1.68 (m, 2H), 1.68–1.59 (m, 2H), 1.46 (p, *J* = 7.2 Hz, 2H), 1.40–1.24 (m, 12H), 0.94–0.86 (m, 3H). ^13^C NMR (101 MHz CD_3_OD): δ 158.7, 135.9, 130.2, 115.5, 69.0, 66.5, 56.8, 37.8, 33.1, 30.73, 30.68, 30.53, 30.48, 30.45, 29.5, 27.2, 23.7, 14.4. HRMS-ESI (*m*/*z*): calcd for C_21_H_38_NO_3_ [M + H]^+^ 352.2852, found: 352.2852.

#### Diethyl 2-Acetamido-2-[2-(4-bromophenyl)-2-oxoethyl]malonate (**51**)

General procedure A was followed using 2-bromo-1-(4-bromophenyl)ethan-1-one (**50**) (1.00 g, 3.60 mmol, 1.4 equiv), DEAM (0.558 g, 2.57 mmol), Cs_2_CO_3_ (1.76 g, 5.40 mmol, 1.5 equiv), and anhydrous MeCN (15 mL). Compound **51** was obtained as an orange solid (429 mg, 40%). ^1^H NMR (400 MHz, CDCl_3_): δ 7.85–7.79 (m, 2H), 7.64–7.58 (m, 2H), 7.09 (s, 1H), 4.31–4.24 (m, 4H), 4.23 (s, 2H), 1.97 (s, 3H), 1.24 (t, *J* = 7.1 Hz, 6H). ^13^C NMR (101 MHz, CDCl_3_): δ 196.1, 169.7, 167.3, 134.9, 132.2, 129.9, 129.2, 64.1, 63.1, 42.3, 23.1, 14.1. HRMS-ESI (*m*/*z*): calcd for C_17_H_21_NO_6_Br [M + H]^+^ 414.0552, found: 414.0553.

#### Diethyl 2-Acetamido-2-[2-(4-octylphenyl)-2-oxoethyl]malonate (**52**)

General procedure B was followed using **51** (0.150 g, 0.362 mmol), *n*-octylboronic acid (68.7 mg, 0.435 mmol, 1.2 equiv), Pd_2_(dba)_3_ (16.6 mg, 0.0181 mmol, 0.05 equiv), SPhos (14.9 mg, 0.0362 mmol, 0.1 equiv), K_2_CO_3_ (0.150 g, 1.09 mmol, 3.0 equiv), and toluene/water (4:1, 2 mL). Compound **52** was obtained as a yellow oil (147 mg, 91%). ^1^H NMR (400 MHz, CDCl_3_): δ 7.91–7.84 (m, 2H), 7.30–7.22 (m, 2H), 7.11 (bs, 1H), 4.33–4.21 (m, 6H), 2.65 (t, *J* = 7.5 Hz, 2H), 1.97 (s, 3H), 1.67–1.55 (m, 2H), 1.35–1.20 (m, 16H), 0.92–0.84 (m, 3H). ^13^C NMR (101 MHz, CDCl_3_): δ 196.7, 169.6, 167.5, 149.8, 134.0, 128.9, 128.5, 64.2, 63.0, 42.3, 36.2, 32.0, 31.2, 29.5, 29.4, 29.3, 23.1, 22.8, 14.2, 14.0. HRMS-ESI (*m*/*z*): calcd for C_25_H_38_NO_6_ [M + H]^+^ 448.2699, found: 448.2700.

#### Diethyl 2-Acetamido-2-[2-(4-decylphenyl)-2-oxoethyl]malonate (**53**)

General procedure B was followed using **51** (0.150 g, 0.362 mmol), *n*-decylboronic acid (80.9 mg, 0.435 mmol, 1.2 equiv), Pd_2_(dba)_3_ (16.6 mg, 0.0181 mmol, 0.05 equiv), SPhos (14.9 mg, 0.0362 mmol, 0.1 equiv), K_2_CO_3_ (0.150 g, 1.09 mmol, 3.0 equiv), and toluene/water (4:1, 2 mL). Compound **53** was obtained as a yellow oil (124 mg, 72%). ^1^H NMR (400 MHz, CDCl_3_): δ 7.91–7.84 (m, 2H), 7.30–7.23 (m, 2H), 7.10 (s, 1H), 4.33–4.21 (m, 6H), 2.65 (t, *J* = 7.9 Hz, 2H), 1.97 (s, 3H), 1.67–1.55 (m, 2H), 1.35–1.20 (m, 20H), 0.92–0.84 (m, 3H). ^13^C NMR (101 MHz, CDCl_3_): δ 196.7, 169.5, 167.5, 149.8, 134.0, 128.9, 128.5, 64.2, 63.0, 42.3, 36.2, 32.0, 31.2, 29.73, 29.68, 29.58, 29.44, 29.38, 23.1, 22.8, 14.2, 14.0. HRMS-ESI (*m*/*z*): calcd for C_27_H_42_NO_6_ [M + H]^+^ 476.3012, found: 476.3013.

#### 3-Amino-3-(hydroxymethyl)-1-(4-octylphenyl)butane-1,4-diol (**54**)

General procedure D was followed using **52** (131 mg, 0.293 mmol), NaBH_4_ (55.4 mg, 1.46 mmol, 5.0 equiv), CaCl_2_ (81.2 mg, 0.732 mmol, 2.5 equiv), and EtOH/water (4:1, 2 mL); N-acetylated diol intermediate (light yellow oil, 104 mg), MeOH/THF (2 mL), and 2 M LiOH (aq., 3 mL). Compound **54** was obtained as a white solid (67 mg, 71%). ^1^H NMR (400 MHz, CD_3_OD): δ 7.31–7.25 (m, 2H), 7.16–7.10 (m, 2H), 4.93 (dd, *J* = 10.5, 2.7 Hz, 1H), 3.57 (q, *J* = 10.8, 6.8 Hz, 2H), 3.50 (s, 2H), 2.58 (t, *J* = 7.6 Hz, 2H), 1.78 (dd, *J* = 14.8, 10.5 Hz, 1H), 1.69 (dd, *J* = 14.7, 2.7 Hz, 1H), 1.65–1.53 (m, 2H), 1.38–1.22 (m, 10H), 0.93–0.85 (m, 3H). ^13^C NMR (101 MHz, CD_3_OD): δ 144.4, 143.0, 129.3, 126.8, 71.6, 67.7, 66.4, 57.3, 44.5, 36.6, 33.0, 32.8, 30.6, 30.4, 30.3, 23.7, 14.4. HRMS-ESI (*m*/*z*): calcd for C_19_H_34_NO_3_ [M + H]^+^ 324.2539, found: 324.2540.^45^

#### 3-Amino-3-(hydroxymethyl)-1-(4-decylphenyl)butane-1,4-diol (**55**)

General procedure D was followed using **53** (112 mg, 0.234 mmol), NaBH_4_ (44.3 mg, 1.17 mmol, 5.0 equiv), CaCl_2_ (65.0 mg, 0.586 mmol, 2.5 equiv), and EtOH/water (4:1, 2 mL); N-acetylated diol intermediate (white solid, 93 mg), MeOH/THF 1:1 (2 mL), and 2 M LiOH (aq.) (3 mL). Compound **55** was obtained as a white solid (60 mg, 73%). ^1^H NMR (400 MHz, CD_3_OD): δ 7.31–7.25 (m, 2H), 7.16–7.10 (m, 2H), 4.93 (dd, *J* = 10.5, 2.7 Hz, 1H), 3.58 (q, *J* = 10.9, 6.9 Hz, 2H), 3.50 (s, 2H), 2.58 (t, *J* = 7.6 Hz, 2H), 1.78 (dd, *J* = 14.7, 10.5 Hz, 1H), 1.69 (dd, *J* = 14.7, 2.7 Hz, 1H), 1.65–1.53 (m, 2H), 1.37–1.22 (m, 14H), 0.94–0.86 (m, 3H). ^13^C NMR (101 MHz, CD_3_OD): δ 144.4, 143.0, 129.3, 126.8, 71.6, 67.7, 66.4, 57.3, 44.5, 36.6, 33.1, 32.8, 30.74, 30.72, 30.6, 30.4, 30.2, 23.7, 14.4. HRMS-ESI (*m*/*z*): calcd for C_21_H_38_NO_3_ [M + H]^+^ 352.2852, found: 352.2856.

#### 2-(5-Bromopyridin-2-yl)ethyl Methanesulfonate (**57**)

To a cooled solution of 2-(5-bromopyridin-2-yl)ethan-1-ol (**56**) (0.500 g, 2.48 mmol) and Et_3_N (0.518 mL, 3.71 mmol, 1.5 equiv) in anhydrous DCM (12 mL) was added methanesulfonyl chloride (0.249 mL, 3.22 mmol, 1.3 equiv), and the reaction mixture was stirred for 3 h. The mixture was diluted with DCM and washed repeatedly with water and brine. The organic layer was dried over anhydrous Na_2_SO_4_ and concentrated on a rotary evaporator to give **57** as a yellow oil (662 mg, 96%). ^1^H NMR (400 MHz, CDCl_3_): δ 8.61 (d, *J* = 2.4 Hz, 1H), 7.76 (dd, *J* = 8.3, 2.4 Hz, 1H), 7.13 (d, *J* = 8.3 Hz, 1H), 4.63 (t, *J* = 6.4 Hz, 2H), 3.18 (t, *J* = 6.4 Hz, 2H), 2.93 (s, 3H). ^13^C NMR (101 MHz, CDCl_3_): δ 155.2, 150.8, 139.3, 125.3, 119.3, 68.6, 37.4, 37.0. HRMS-ESI (*m*/*z*): calcd for C_8_H_11_NO_3_SBr [M + H]^+^ 279.9643, found: 279.9643.

#### Diethyl 2-Acetamido-2-[2-(5-bromopyridin-2-yl)ethyl]malonate (**58**)

General procedure A was followed using **57** (0.661 g, 2.36 mmol, 1.2 equiv), DEAM (0.427 g, 1.97 mmol), Cs_2_CO_3_ (1.28 g, 3.93 mmol, 2.0 equiv), and anhydrous MeCN (15 mL). Compound **58** was obtained as a yellow solid (342 mg, 43%). ^1^H NMR (400 MHz, CDCl_3_): δ 8.55 (d, *J* = 2.2 Hz, 1H), 7.70 (dd, *J* = 8.3, 2.4 Hz, 1H), 7.05 (d, *J* = 8.2 Hz, 1H), 6.83 (s, 1H), 4.31–4.14 (m, 4H), 2.79–2.71 (m, 2H), 2.69–2.61 (m, 2H), 2.00 (s, 3H), 1.25 (t, *J* = 7.1 Hz, 6H). ^13^C NMR (101 MHz, CDCl_3_): δ 169.3, 168.1, 159.3, 150.3, 139.0, 124.2, 118.4, 66.3, 62.8, 32.2, 32.1, 23.2, 14.1. HRMS-ESI (*m*/*z*): calcd for C_16_H_22_N_2_O_5_Br [M + H]^+^ 401.0712, found: 401.0711.

#### Diethyl 2-Acetamido-2-[2-(5-octylpyridin-2-yl)ethyl]malonate (**59**)

General procedure B was followed using **58** (0.100 g, 0.249 mmol), *n*-octylboronic acid (47.3 mg, 0.299 mmol, 1.2 equiv), Pd_2_(dba)_3_ (11.4 mg, 0.0125 mmol, 0.05 equiv), SPhos (10.2 mg, 0.0250 mmol, 0.1 equiv), K_2_CO_3_ (0.103 g, 0.748 mmol, 3.0 equiv), and toluene/water (4:1, 2 mL). Compound **59** was obtained as a yellow oil (51 mg, 47%). ^1^H NMR (400 MHz, CDCl_3_): δ 8.30 (d, *J* = 2.0 Hz, 1H), 7.39 (dd, *J* = 7.9, 2.4 Hz, 1H), 7.05 (d, *J* = 7.9 Hz, 1H), 6.94 (bs, 1H), 4.29–4.12 (m, 4H), 2.80–2.70 (m, 2H), 2.71–2.62 (m, 2H), 2.55 (t, *J* = 7.5 Hz, 2H), 1.99 (s, 3H), 1.56 (p, *J* = 7.3 Hz, 2H), 1.34–1.20 (m, 16H), 0.91–0.83 (m, 3H). ^13^C NMR (101 MHz, CDCl_3_): δ 169.2, 168.2, 157.8, 149.3, 136.5, 135.6, 122.3, 66.4, 62.7, 32.7, 32.4, 32.3, 32.0, 31.4, 29.5, 29.4, 29.3, 23.2, 22.8, 14.2, 14.1. HRMS-ESI (*m*/*z*): calcd for C_24_H_39_N_2_O_5_ [M + H]^+^ 435.2859, found: 435.2851.

#### Diethyl 2-Acetamido-2-[2-(5-decylpyridin-2-yl)ethyl]malonate (**60**)

General procedure B was followed using **58** (0.100 g, 0.249 mmol), *n*-decylboronic acid (55.6 mg, 0.299 mmol, 1.2 equiv), Pd_2_(dba)_3_ (11.4 mg, 0.0125 mmol, 0.05 equiv), SPhos (10.2 mg, 0.0250 mmol, 0.1 equiv), K_2_CO_3_ (0.103 g, 0.748 mmol, 3.0 equiv), and toluene/water (4:1, 2 mL). Compound **60** was obtained as a yellow oil (69 mg, 60%). ^1^H NMR (400 MHz, CDCl_3_): δ 8.30 (d, *J* = 2.3 Hz, 1H), 7.39 (dd, *J* = 7.9, 2.3 Hz, 1H), 7.05 (d, *J* = 8.0 Hz, 1H), 6.95 (bs, 1H), 4.30–4.12 (m, 4H), 2.80–2.70 (m, 2H), 2.71–2.60 (m, 2H), 2.54 (t, *J* = 7.5 Hz, 2H), 1.99 (s, 3H), 1.56 (p, *J* = 7.4 Hz, 2H), 1.33–1.20 (m, 20H), 0.91–0.83 (m, 3H). ^13^C NMR (101 MHz, CDCl_3_): δ 169.2, 168.2, 157.8, 149.3, 136.5, 135.6, 122.3, 66.4, 62.7, 32.7, 32.4, 32.3, 32.0, 31.4, 29.73, 29.70, 29.6, 29.4, 29.3, 23.2, 22.8, 14.2, 14.1. HRMS-ESI (*m*/*z*): calcd for C_26_H_43_N_2_O_5_ [M + H]^+^ 463.3172, found: 463.3173.

#### 2-Amino-2-[2-(5-octylpyridin-2-yl)ethyl]propane-1,3-diol (**61**)

General procedure D was followed using **59** (47.5 mg, 0.109 mmol), NaBH_4_ (20.7 mg, 0.547 mmol, 5.0 equiv), CaCl_2_ (30.3 mg, 0.273 mmol, 2.5 equiv), and EtOH/water (4:1, 1 mL); N-acetylated diol intermediate (yellow oil, 33 mg), MeOH/THF (1 mL), and 2 M LiOH (aq., 2 mL). Compound **61** was obtained as a white solid (18 mg, 52%). ^1^H NMR (400 MHz, CD_3_OD): δ 8.25 (d, *J* = 1.8 Hz, 1H), 7.59 (dd, *J* = 8.0, 2.3 Hz, 1H), 7.25 (d, *J* = 8.0 Hz, 1H), 3.49 (q, *J* = 13.3, 11.0 Hz, 4H), 2.83–2.75 (m, 2H), 2.61 (t, *J* = 7.5 Hz, 2H), 1.77–1.68 (m, 2H), 1.61 (p, *J* = 7.3, 6.7 Hz, 2H), 1.40–1.22 (m, 10H), 0.93–0.85 (m, 3H). ^13^C NMR (101 MHz, CD_3_OD): δ 160.7, 149.3, 138.8, 137.4, 124.2, 66.3, 57.0, 35.8, 33.4, 33.0, 32.3, 31.9, 30.5, 30.4, 30.2, 23.7, 14.4. HRMS-ESI (*m*/*z*): calcd for C_18_H_33_N_2_O_2_ [M + H]^+^ 309.2542, found: 309.2542.

#### 2-Amino-2-[2-(5-decylpyridin-2-yl)ethyl]propane-1,3-diol (**62**)

General procedure D was followed using **60** (64.7 mg, 0.140 mmol), NaBH_4_ (26.5 mg, 0.700 mmol, 5.0 equiv), CaCl_2_ (38.8 mg, 0.350 mmol, 2.5 equiv), and EtOH/water (4:1, 1 mL); N-acetylated diol intermediate (transparent oil, 51 mg), MeOH/THF (1 mL), and 2 M LiOH (aq., 2 mL). Compound **62** was obtained as a white solid (15 mg, 31%). ^1^H NMR (400 MHz, CD_3_OD): δ 8.25 (d, *J* = 1.8 Hz, 1H), 7.59 (dd, *J* = 8.0, 2.3 Hz, 1H), 7.25 (d, *J* = 7.9 Hz, 1H), 3.49 (q, *J* = 13.3, 11.1 Hz, 4H), 2.83–2.75 (m, 2H), 2.61 (t, *J* = 7.6 Hz, 2H), 1.77–1.68 (m, 2H), 1.61 (p, *J* = 7.2 Hz, 2H), 1.39–1.21 (m, 14H), 0.94–0.86 (m, 3H). ^13^C NMR (101 MHz, CD_3_OD): δ 160.7, 149.3, 138.8, 137.4, 124.2, 66.3, 57.0, 35.8, 33.4, 33.1, 32.3, 32.0, 30.7, 30.5, 30.4, 30.2, 23.7, 14.4. HRMS-ESI (*m*/*z*): calcd for C_20_H_37_N_2_O_2_ [M + H]^+^ 337.2855, found: 337.2858.

#### Ethyl 2-(6-Bromoquinolin-2-yl)acetate (**65**)

A mixture of diisopropylamine (1.89 mL, 13.5 mmol, 3.0 equiv) and anhydrous THF (2.0 mL) was cooled to −78 °C under an argon atmosphere. *N*-Butyllithium (a 2.5 M solution in hexane, 5.04 mL, 12.6 mmol, 2.8 equiv) was slowly added and the mixture was stirred for 30 min. Then, a solution of 6-bromoquinaldine (**64**, 1.00 g, 4.50 mmol) in dry THF (4.0 mL) was slowly added. After 30 min, diethyl carbonate (1.91 mL, 15.8 mmol, 3.5 equiv) was slowly added and the mixture was stirred for 2 h. The reaction was quenched with water and extracted with EtOAc. The combined organic layers were washed with brine, dried over anhydrous Na_2_SO_4_, filtered, and concentrated on a rotary evaporator, to give compound **65** as an orange solid (1.28 g, 97%). ^1^H NMR (400 MHz, CDCl_3_): δ 8.04 (d, *J* = 8.5 Hz, 1H), 7.96 (d, *J* = 2.2 Hz, 1H), 7.93 (d, *J* = 8.9 Hz, 1H), 7.76 (dd, *J* = 9.0, 2.2 Hz, 1H), 7.46 (d, *J* = 8.5 Hz, 1H), 4.20 (q, *J* = 7.1 Hz, 2H), 4.02 (s, 2H), 1.27 (t, *J* = 7.1 Hz, 3H). ^13^C NMR (101 MHz, CDCl_3_): δ 170.4, 155.5, 146.6, 135.7, 133.2, 131.0, 129.7, 128.3, 122.8, 120.4, 61.4, 44.9, 14.3. HRMS-ESI (*m*/*z*): calcd for C_13_H_13_NO_2_Br [M + H]^+^ 294.0130, found: 294.0129.

#### 2-(6-Bromoquinolin-2-yl)ethan-1-ol (66)

To a solution of **65** (1.25 g, 4.24 mmol) in anhydrous THF (10 mL) was added a 1 M solution of DIBAL-H in THF (12.7 mL, 12.7 mmol, 3.0 equiv) dropwise over 30 min at −78 °C and under an argon atmosphere. After the completion of the addition, the mixture was warmed to −10 °C and stirred for 3 h. The reaction was quenched with methanol. Then, 10% Rochelle′s salt and EtOAc were added and the mixture was stirred vigorously for 30 min. The layers were separated and the water layer was further extracted with EtOAc. The combined organic layers were washed with brine, dried over anhydrous Na_2_SO_4_, filtered, and concentrated on a rotary evaporator. Column chromatography, with an increasing gradient of EtOAc (starting with 30%) in *n*-heptane, gave compound **66** as yellow solid (0.848 g, 79%). ^1^H NMR (400 MHz, CDCl_3_): δ 8.00 (d, *J* = 8.3 Hz, 1H), 7.94 (d, *J* = 2.2 Hz, 1H), 7.88 (d, *J* = 8.9 Hz, 1H), 7.75 (dd, *J* = 9.0, 2.2 Hz, 1H), 7.29 (d, *J* = 8.5 Hz, 1H), 4.46 (bs, 1H), 4.14 (t, *J* = 5.5 Hz, 2H), 3.19 (t, *J* = 5.5 Hz, 2H). ^13^C NMR (101 MHz, CDCl_3_): δ 162.1, 146.1, 135.7, 133.2, 130.7, 129.8, 128.1, 122.9, 112.0, 61.3, 39.5. HRMS-ESI (*m*/*z*): calcd for C_11_H_11_NOBr [M + H]^+^ 225.0024, found: 225.0025.

#### 2-(6-Bromoquinolin-2-yl)ethyl Methanesulfonate (**67**)

To a cooled solution of **66** (0.750 g, 2.96 mmol) and Et_3_N (0.62 mL, 4.45 mmol, 1.5 equiv) in anhydrous DCM (20 mL) was added methanesulfonyl chloride (0.298 mL, 3.85 mmol, 1.3 equiv), and the reaction mixture was stirred for 3 h. The mixture was diluted with DCM and washed with water and brine. The organic layer was dried over anhydrous Na_2_SO_4_, filtered, and concentrated on a rotary evaporator. Greyish solid (0.901 g, 92%). ^1^H NMR (400 MHz, CDCl_3_): δ 8.03 (d, *J* = 8.3 Hz, 1H), 7.97 (d, *J* = 2.2 Hz, 1H), 7.88 (d, *J* = 8.9 Hz, 1H), 7.77 (dd, *J* = 9.0, 2.2 Hz, 1H), 7.35 (d, *J* = 8.4 Hz, 1H), 4.80 (t, *J* = 6.5 Hz, 2H), 3.40 (t, *J* = 6.5 Hz, 2H), 2.94 (s, 3H). ^13^C NMR (101 MHz, CDCl_3_): δ 157.7, 146.6, 135.8, 133.3, 130.7, 129.8, 128.3, 122.9, 120.3, 68.7, 38.0, 37.4. HRMS-ESI (*m*/*z*): calcd for C_12_H_13_NO_3_SBr [M + H]^+^ 329.9800, found: 329.9799.

#### 6-Bromo-2-(bromomethyl)quinolone (**69**)

To a solution of 6-bromoquinaldine (**64**, 1.00 g, 4.50 mmol) in MeCN (20 mL) was added *tert*-butyl hydroperoxide (4.64 mL, 36.0 mmol, 8.0 equiv) and copper bromide (0.904 g, 6.30 mmol, 1.4 equiv), and the mixture was stirred at 70 °C for 8 h. Then, it was cooled to room temperature and portioned between water and DCM. The layers were separated and the aqueous layer was extracted with DCM. Residual bromide in the organic phase was quenched with a saturated solution of sodium thiosulphate in H_2_O. The layers were separated and the organic layer was washed with a saturated solution of NH_4_Cl in H_2_O and brine, dried over Na_2_SO_4_, filtered, and concentrated on a rotary evaporator. Column chromatography, with an increasing gradient of EtOAc (starting with 0%) in *n*-heptane, gave compound **69** as a white solid (358 mg, 26%). ^1^H NMR (400 MHz, CDCl_3_): δ 8.09 (d, *J* = 8.6 Hz, 1H), 7.98 (d, *J* = 2.2 Hz, 1H), 7.93 (d, *J* = 9.0 Hz, 1H), 7.79 (dd, *J* = 9.0, 2.2 Hz, 1H), 7.59 (d, *J* = 8.5 Hz, 1H), 4.69 (s, 2H). ^13^C NMR (101 MHz, CDCl_3_): δ 157.5, 146.3, 136.4, 133.6, 131.1, 129.7, 128.6, 122.2, 121.2, 34.2. HRMS-ESI (*m*/*z*): calcd for C_10_H_8_NBr [M + H]^+^ 299.9024, found: 299.9023.

#### Diethyl 2-Acetamido-2-[(6-bromonaphthalen-2-yl)methyl]malonate (70)

General procedure A was followed using 2-bromo-6-(bromomethyl)naphthalene (**63**, 0.250 g, 0.833 mmol, 1.4 equiv), DEAM (0.129 g, 0.595 mmol), Cs_2_CO_3_ (0.291 g, 0.893 mmol, 1.5 equiv), and anhydrous MeCN/DMF (1:1, 4 mL). Compound **70** was obtained as a yellow solid (214 mg, 83%). ^1^H NMR (400 MHz, CDCl_3_): δ 7.96 (d, *J* = 2.0 Hz, 1H), 7.64 (d, *J* = 8.5 Hz, 1H), 7.60 (d, *J* = 8.9 Hz, 1H), 7.52 (dd, *J* = 8.7, 2.0 Hz, 1H), 7.45 (s, 1H), 7.14 (dd, *J* = 8.4, 1.7 Hz, 1H), 6.52 (s, 1H), 4.35–4.23 (m, 4H), 3.81 (s, 2H), 2.04 (s, 3H), 1.31 (t, *J* = 7.1 Hz, 6H). ^13^C NMR (101 MHz, CDCl_3_): δ 169.3, 167.6, 133.7, 133.6, 131.8, 129.8, 129.7, 129.3, 129.0, 128.3, 127.1, 119.9, 67.3, 62.9, 38.0, 23.2, 14.2. HRMS-ESI (*m*/*z*): calcd for C_20_H_23_NO_5_Br [M + H]^+^ 436.0760, found: 436.0761.

#### Diethyl 2-Acetamido-2-[2-(6-bromoquinolin-2-yl)ethyl]malonate (71)

General procedure A was followed using **67** (0.500 g, 1.51 mmol, 1.2 equiv), DEAM (0.274 g, 1.26 mmol), Cs_2_CO_3_ (0.617 g, 1.89 mmol, 1.5 equiv), and anhydrous DMF (10 mL). Compound **71** was obtained as an orange solid (0.215 g, 38%). ^1^H NMR (400 MHz, CDCl_3_): δ 7.97 (d, *J* = 8.4 Hz, 1H), 7.93 (d, *J* = 2.2 Hz, 1H), 7.87 (d, *J* = 9.0 Hz, 1H), 7.74 (dd, *J* = 9.0, 2.2 Hz, 1H), 7.30 (d, *J* = 8.5 Hz, 1H), 6.90 (s, 1H), 4.29–4.12 (m, 4H), 2.93–2.83 (m, 4H), 1.97 (s, 3H), 1.24 (t, *J* = 7.1 Hz, 6H). ^13^C NMR (101 MHz, CDCl_3_): δ 169.3, 168.1, 161.6, 146.5, 135.4, 133.0, 130.8, 129.7, 128.0, 122.2, 119.8, 66.4, 62.8, 33.6, 31.8, 23.2, 14.1. HRMS-ESI (*m*/*z*): calcd for C_20_H_24_N_2_O_5_Br [M + H]^+^ 451.0869, found: 457.0870.

#### Diethyl 2-Acetamido-2-[(7-chloroquinolin-2-yl)methyl]malonate (72)

General procedure A was followed using 2-(bromomethyl)-7-chloroquinoline (**68**, 90.0 mg, 0.531 mmol, 1.4 equiv), DEAM (54.5 mg, 0.251 mmol), Cs_2_CO_3_ (0.164 g, 0.502 mmol, 2.0 equiv), and anhydrous MeCN (3 mL). Compound **72** was obtained as a pink solid (69 mg, 70%). ^1^H NMR (400 MHz, CDCl_3_): δ 8.01 (d, *J* = 8.4 Hz, 1H), 7.89 (d, *J* = 2.0 Hz, 1H), 7.71 (d, *J* = 8.7 Hz, 1H), 7.44 (dd, *J* = 8.7, 2.1 Hz, 1H), 7.20 (d, *J* = 8.4 Hz, 1H), 6.87 (s, 1H), 4.32 (q, *J* = 7.1 Hz, 4H), 4.06 (s, 2H), 1.92 (s, 3H), 1.29 (t, *J* = 7.1 Hz, 6H). ^13^C NMR (101 MHz, CDCl_3_): δ 169.4, 167.9, 158.4, 148.0, 136.1, 135.4, 128.9, 128.0, 127.3, 125.3, 122.8, 66.2, 62.8, 40.5, 23.1, 14.1. HRMS-ESI (*m*/*z*): calcd for C_19_H_22_N_2_O_5_Cl [M + H]^+^ 393.1217, found: 393.1220.

#### Diethyl 2-Acetamido-2-[(6-bromoquinolin-2-yl)methyl]malonate (73)

General procedure A was followed using **69** (0.350 g, 1.16 mmol, 1.2 equiv), DEAM (0.230 g, 1.06 mmol), Cs_2_CO_3_ (0.517 g, 1.59 mmol, 1.5 equiv), and anhydrous DMF (10 mL). Compound **73** was obtained as a brown solid (295 mg, 64%). ^1^H NMR (400 MHz, CDCl_3_): δ 7.98–7.92 (m, 2H), 7.78 (d, *J* = 9.0 Hz, 1H), 7.72 (dd, *J* = 8.9, 2.1 Hz, 1H), 7.23 (d, *J* = 8.4 Hz, 1H), 6.87 (s, 1H), 4.31 (q, *J* = 7.1 Hz, 4H), 4.05 (s, 2H), 1.92 (s, 3H), 1.29 (t, *J* = 7.1 Hz, 6H). ^13^C NMR (101 MHz, CDCl_3_): δ 169.4, 167.9, 157.7, 146.2, 135.3, 133.0, 130.7, 129.8, 128.1, 123.4, 120.1, 66.2, 62.8, 40.5, 23.1, 14.1. HRMS-ESI (*m*/*z*): calcd for C_19_H_22_N_2_O_5_Br [M + H]^+^ 437.0712, found: 437.0711.

#### Diethyl 2-Acetamido-2-[(6-octylnaphthalen-2-yl)methyl]malonate (74)

General procedure B was followed using **70** (0.100 g, 0.229 mmol), *n*-octylboronic acid (43.5 mg, 0.275 mmol, 1.2 equiv), Pd_2_(dba)_3_ (10.5 mg, 0.0115 mmol, 0.05 equiv), SPhos (9.4 mg, 0.023 mmol, 0.1 equiv), K_2_CO_3_ (95.0 mg, 0.688 mmol, 3.0 equiv), and toluene/water (4:1, 2 mL). Compound **74** was obtained as a white solid (85 mg, 79%). ^1^H NMR (400 MHz, CDCl_3_): δ 7.65 (dd, *J* = 8.4, 5.2 Hz, 2H), 7.56 (s, 1H), 7.43 (s, 1H), 7.31 (dd, *J* = 8.4, 1.7 Hz, 1H), 7.08 (dd, *J* = 8.4, 1.8 Hz, 1H), 6.51 (s, 1H), 4.29 (q, *J* = 7.1 Hz, 4H), 3.80 (s, 2H), 2.74 (t, *J* = 7.4 Hz, 2H), 2.03 (s, 3H), 1.74–1.62 (m, 2H), 1.36–1.23 (m, 16H), 0.93–0.84 (m, 3H). ^13^C NMR (101 MHz, CDCl_3_): δ 169.3, 167.7, 140.7, 132.9, 132.0, 131.9, 128.7, 127.9, 127.6, 127.5, 126.2, 67.5, 62.8, 38.0, 36.2, 32.0, 31.6, 29.6, 29.5, 29.4, 23.2, 22.8, 14.24, 14.21. HRMS-ESI (*m*/*z*): calcd for C_28_H_40_NO_5_ [M + H]^+^ 470.2906, found: 470.2908.

#### Diethyl 2-Acetamido-2-[2-(6-octylquinolin-2-yl)ethyl]malonate (75)

General procedure B was followed using **71** (0.100 g, 0.222 mmol), *n*-octylboronic acid (42.0 mg, 0.266 mmol, 1.2 equiv), Pd_2_(dba)_3_ (10.1 mg, 0.0111 mmol, 0.05 equiv), SPhos (9.1 mg, 0.022 mmol, 0.1 equiv), K_2_CO_3_ (91.9 mg, 0.665 mmol, 3.0 equiv), and toluene/water (4:1, 2 mL). Compound **75** was obtained as a white solid (91 mg, 84%). ^1^H NMR (400 MHz, CDCl_3_): δ 7.99 (d, *J* = 8.4 Hz, 1H), 7.91 (d, *J* = 9.1 Hz, 1H), 7.56–7.49 (m, 2H), 7.24 (d, *J* = 8.4 Hz, 1H), 6.99 (s, 1H), 4.29–4.11 (m, 4H), 2.88 (s, 4H), 2.76 (t, *J* = 7.5 Hz, 2H), 1.97 (s, 3H), 1.69 (p, *J* = 7.5 Hz, 2H), 1.40–1.20 (m, 16H), 0.91–0.83 (m, 3H). ^13^C NMR (101 MHz, CDCl_3_): δ 169.3, 168.2, 160.1, 146.7, 140.9, 136.0, 131.2, 128.7, 126.9, 125.9, 121.2, 66.5, 62.7, 36.0, 33.6, 32.1, 32.0, 31.4, 29.6, 29.44, 29.39, 23.2, 22.8, 14.2, 14.1. HRMS-ESI (*m*/*z*): calcd for C_28_H_41_N_2_O_5_ [M + H]^+^ 485.3015, found: 485.3016.

#### Diethyl 2-Acetamido-2-[2-(6-decylquinolin-2-yl)ethyl]malonate (76)

General procedure B was followed using **71** (0.100 g, 0.222 mmol), *n*-decylboronic acid (49.0 mg, 0.266 mmol, 1.2 equiv), Pd_2_(dba)_3_ (10.1 mg, 0.0111 mmol, 0.05 equiv), SPhos (9.1 mg, 0.0222 mmol, 0.1 equiv), K_2_CO_3_ (91.9 mg, 0.665 mmol, 3.0 equiv), and toluene/water (4:1, 2 mL). Compound **76** was obtained as an orange solid (72 mg, 64%). ^1^H NMR (400 MHz, CDCl_3_): δ 8.00 (d, *J* = 8.5 Hz, 1H), 7.92 (d, *J* = 9.0 Hz, 1H), 7.56–7.50 (m, 2H), 7.24 (s, 0H), 7.00 (s, 1H), 4.29–4.11 (m, 4H), 2.88 (s, 4H), 2.76 (t, *J* = 7.5 Hz, 2H), 1.97 (s, 3H), 1.69 (p, *J* = 7.5 Hz, 2H), 1.38–1.20 (m, 20H), 0.91–0.83 (m, 3H). ^13^C NMR (101 MHz, CDCl_3_): δ 169.3, 168.2, 160.1, 146.7, 140.9, 136.1, 131.2, 128.7, 126.9, 125.9, 121.2, 66.5, 62.7, 36.0, 33.6, 32.1, 32.0, 31.4, 29.75, 29.73, 29.65, 29.5, 29.4, 23.2, 22.8, 14.3, 14.1. HRMS-ESI (*m*/*z*): calcd for C_30_H_45_N_2_O_5_ [M + H]^+^ 513.3328, found: 513.3327.

#### Diethyl 2-Acetamido-2-[(7-octylquinolin-2-yl)methyl]malonate (77)

General procedure B was followed using **72** (68.0 mg, 0.173 mmol), *n*-octylboronic acid (32.9 mg, 0.208 mmol, 1.2 equiv), Pd_2_(dba)_3_ (7.9 mg, 0.0087 mmol, 0.05 equiv), SPhos (7.1 mg, 0.017 mmol, 0.1 equiv), K_2_CO_3_ (71.7 mg, 0.519 mmol, 3.0 equiv), and toluene/water (4:1, 2 mL). Compound **77** was obtained as a yellow solid (75 mg, 93%). ^1^H NMR (400 MHz, CDCl_3_): δ 7.99 (d, *J* = 8.3 Hz, 1H), 7.71–7.64 (m, 2H), 7.34 (dd, *J* = 8.2, 1.8 Hz, 1H), 7.13 (d, *J* = 8.3 Hz, 1H), 6.96 (s, 1H), 4.32 (q, *J* = 7.1 Hz, 4H), 4.03 (s, 2H), 2.79 (t, *J* = 7.6 Hz, 2H), 1.93 (s, 3H), 1.75–1.65 (m, 2H), 1.42–1.22 (m, 16H), 0.91–0.84 (m, 3H). ^13^C NMR (101 MHz, CDCl_3_): δ 169.4, 168.0, 156.9, 147.9, 144.9, 136.0, 127.8, 127.4, 125.3, 121.7, 66.4, 62.7, 40.6, 36.3, 32.0, 31.4, 29.6, 29.5, 29.4, 23.2, 22.8, 14.2, 14.1. HRMS-ESI (*m*/*z*): calcd for C_27_H_39_N_2_O_5_ [M + H]^+^ 471.2859, found: 471.2859.

#### Diethyl 2-Acetamido-2-[(6-decylquinolin-2-yl)methyl]malonate (78)

General procedure B was followed using **73** (0.150 g, 0.229 mmol), *n*-decylboronic acid (51.1 mg, 0.274 mmol, 1.2 equiv), Pd_2_(dba)_3_ (10.4 mg, 0.0114 mmol, 0.05 equiv), SPhos (9.4 mg, 0.023 mmol, 0.1 equiv), K_2_CO_3_ (94.8 mg, 0.686 mmol, 3.0 equiv), and toluene/water (4:1, 2 mL). Compound **78** was obtained as an orange solid (107 mg, 63%). ^1^H NMR (400 MHz, CDCl_3_): δ 7.96 (d, *J* = 8.3 Hz, 1H), 7.81 (d, *J* = 8.3 Hz, 1H), 7.55–7.47 (m, 2H), 7.16 (d, *J* = 8.4 Hz, 1H), 6.94 (s, 1H), 4.38–4.26 (m, 4H), 4.02 (s, 2H), 2.76 (t, *J* = 7.6 Hz, 2H), 1.93 (s, 3H), 1.69 (p, *J* = 7.5 Hz, 2H), 1.46–1.19 (m, 20H), 0.93–0.83 (m, 3H). ^13^C NMR (101 MHz, CDCl_3_): δ 169.4, 168.0, 156.1, 146.4, 141.1, 135.8, 131.1, 128.7, 127.0, 126.0, 122.5, 66.4, 62.7, 40.5, 36.0, 32.0, 31.5, 29.75, 29.73, 29.65, 29.5, 29.4, 23.2, 22.8, 14.3, 14.1. HRMS-ESI (*m*/*z*): calcd for C_29_H_43_N_2_O_5_ [M + H]^+^ 499.3172, found: 499.3173.

#### 2-Amino-2-[(6-octylnaphthalen-2-yl)methyl]propane-1,3-diol (79)

General procedure D was followed using **74** (82.9 mg, 0.177 mmol), NaBH_4_ (33.4 mg, 0.883 mmol, 5.0 equiv), CaCl_2_ (49.0 mg, 0.441 mmol, 2.5 equiv), and EtOH/water (4:1, 2 mL); N-acetylated diol intermediate (white solid, 71 mg), MeOH/THF (1 mL), and 2 M LiOH (aq., 2 mL). Compound **79** was obtained as a white solid (37 mg, 60%). ^1^H NMR (400 MHz, CD_3_OD): δ 7.72 (dd, *J* = 8.4, 3.9 Hz, 2H), 7.68 (s, 1H), 7.58 (s, 1H), 7.39 (dd, *J* = 8.4, 1.8 Hz, 1H), 7.32 (dd, *J* = 8.4, 1.7 Hz, 1H), 3.43 (q, *J* = 10.8, 1.0 Hz, 4H), 2.87 (s, 2H), 2.76 (t, *J* = 7.5 Hz, 2H), 1.69 (p, *J* = 7.2 Hz, 2H), 1.42–1.20 (m, 10H), 0.92–0.84 (m, 3H). ^13^C NMR (101 MHz, CD_3_OD): δ 141.2, 134.8, 134.0, 133.4, 130.1, 129.9, 128.5, 128.5, 128.2, 127.0, 66.0, 57.6, 40.6, 37.0, 33.0, 32.6, 30.6, 30.41, 30.36, 23.7, 14.4. HRMS-ESI (*m*/*z*): calcd for C_22_H_34_NO_2_ [M + H]^+^ 344.2590, found: 344.2592.

#### 2-Amino-2-[2-(6-octylquinolin-2-yl)ethyl]propane-1,3-diol (80)

To a cooled solution of **75** (85.0 mg, 0.183 mmol) in anhydrous THF (2.0 mL) was added a 1 M solution of LiAlH_4_ in THF (0.37 mL, 0.37 mmol, 2.0 equiv) dropwise over 10 min, and the mixture was stirred for 1 h on an ice bath. Then, it was warmed up to room temperature and stirred for an additional 1 h. The reaction was slowly quenched with water. Then, 10% Rochelle’s salt and EtOAc were added and the mixture was stirred vigorously overnight. The layers were separated and the water layer was further extracted with EtOAc. The combined organic layers were washed with brine, dried over anhydrous Na_2_SO_4_, filtered, and concentrated on a rotary evaporator to give an N-acetylated diol intermediate as a yellow oil (72 mg). The intermediate was dissolved in THF/MeOH (2 mL), 2 M LiOH (aq., 2 mL) was added, and the reaction mixture was refluxed for 4 h. The reaction mixture was cooled to room temperature and portioned between water and EtOAc. The layers were separated and the aqueous layer was extracted with EtOAc. The combined organic layers were washed with brine, dried over anhydrous Na_2_SO_4_, filtered, and concentrated on a rotary evaporator. Column chromatography, using a Biotage SNAP KP-NH column and an increasing gradient of MeOH in DCM, starting with 2% of MeOH, gave compound **80** as a pale yellow solid (19 mg, 29%). ^1^H NMR (400 MHz, CD_3_OD): δ 8.19 (d, *J* = 8.4 Hz, 1H), 7.87 (d, *J* = 8.6 Hz, 1H), 7.66 (d, *J* = 1.9 Hz, 1H), 7.60 (dd, *J* = 8.7, 2.0 Hz, 1H), 7.44 (d, *J* = 8.5 Hz, 1H), 3.54 (q, *J* = 10.9 Hz, 4H), 3.04–2.96 (m, 2H), 2.80 (t, *J* = 7.5 Hz, 2H), 1.89–1.80 (m, 2H), 1.72 (p, *J* = 7.5 Hz, 2H), 1.43–1.22 (m, 10H), 0.92–0.84 (m, 3H). ^13^C NMR (101 MHz, CD_3_OD): δ 163.6, 147.2, 142.3, 138.3, 132.5, 128.4, 128.2, 127.3, 122.8, 66.3, 57.1, 36.7, 35.5, 33.2, 33.0, 32.5, 30.6, 30.38, 30.35, 23.7, 14.4. HRMS-ESI (*m*/*z*): calcd for C_22_H_35_N_2_O_2_ [M + H]^+^ 359.2699, found: 359.2698.

#### 2-Amino-2-[2-(6-decylquinolin-2-yl)ethyl]propane-1,3-diol (81)

Synthesis was performed as described for compound **80** using **76** (65.0 mg, 0.127 mmol), a 1 M solution of LiAlH_4_ in THF (0.25 mL, 0.25 mmol, 2.0 equiv), and anhydrous THF (2.0 mL); N-acetylated diol intermediate (yellow oil, 56 mg), THF/MeOH (1.5 mL), and 2 M LiOH (aq. 2 mL). Compound **81** was obtained as a pale yellow solid (19 mg, 40%). ^1^H NMR (400 MHz, CD_3_OD): δ 8.19 (d, *J* = 8.3 Hz, 1H), 7.87 (d, *J* = 8.6 Hz, 1H), 7.66 (d, *J* = 1.9 Hz, 1H), 7.60 (dd, *J* = 8.7, 2.0 Hz, 1H), 7.44 (d, *J* = 8.4 Hz, 1H), 3.53 (q, *J* = 12.1, 11.0 Hz, 4H), 3.04–2.96 (m, 2H), 2.80 (t, *J* = 7.6 Hz, 2H), 1.89–1.80 (m, 2H), 1.71 (p, *J* = 7.4 Hz, 2H), 1.42–1.23 (m, 14H), 0.92–0.84 (m, 3H). ^13^C NMR (101 MHz, CD_3_OD): δ 163.6, 147.2, 142.3, 138.3, 132.5, 128.4, 128.2, 127.3, 122.8, 66.3, 57.2, 36.7, 35.5, 33.2, 33.1, 32.4, 30.7, 30.6, 30.4, 30.3, 23.7, 14.4. HRMS-ESI (*m*/*z*): calcd for C_24_H_39_N_2_O_2_ [M + H]^+^ 387.3012, found: 387.3013.

#### 2-Amino-2-[(7-octylquinolin-2-yl)methyl]propane-1,3-diol (82)

General procedure D was followed using **77** (74.0 mg, 0.157 mmol), NaBH_4_ (29.7 mg, 0.785 mmol, 5.0 equiv), CaCl_2_ (43.6 mg, 0.393 mmol, 2.5 equiv), and EtOH/water (4:1, 2 mL); N-acetylated diol intermediate (yellow oil, 59 mg), MeOH/THF (2 mL), and 2 M LiOH (aq., 2 mL). Compound **82** was obtained as a white solid (28 mg, 51%). ^1^H NMR (400 MHz, CD_3_OD): δ 8.20 (d, *J* = 8.4 Hz, 1H), 7.81 (d, *J* = 8.3 Hz, 1H), 7.75 (s, 1H), 7.45–7.40 (m, 2H), 3.47 (s, 4H), 3.09 (s, 2H), 2.82 (t, *J* = 7.6 Hz, 2H), 1.73 (p, *J* = 7.4 Hz, 2H), 1.44–1.21 (m, 10H), 0.92–0.84 (m, 3H). ^13^C NMR (101 MHz, CD_3_OD): δ 160.3, 148.7, 146.5, 137.9, 129.0, 128.7, 127.4, 126.7, 124.0, 66.8, 58.5, 43.6, 37.1, 33.0, 32.3, 30.6, 30.4, 30.3, 23.7, 14.4. HRMS-ESI (*m*/*z*): calcd for C_21_H_33_N_2_O_2_ [M + H]^+^ 345.2542, found: 345.2544.

#### 2-Amino-2-[(6-decylquinolin-2-yl)methyl]propane-1,3-diol (83)

General procedure D was followed using **78** (0.100 g, 0.201 mmol), NaBH_4_ (37.9 mg, 1.00 mmol, 5.0 equiv), CaCl_2_ (55.6 mg, 0.501 mmol, 2.5 equiv), and EtOH/water (4:1, 2 mL); N-acetylated diol intermediate (yellow solid, 83 mg), MeOH/THF (2 mL), and 2 M LiOH (aq., 2 mL). Compound **83** was obtained as a light yellow solid (13 mg, 17%).^1^H NMR (400 MHz, CD_3_OD): δ 8.19 (d, *J* = 8.4 Hz, 1H), 7.89 (d, *J* = 8.6 Hz, 1H), 7.67 (d, *J* = 1.9 Hz, 1H), 7.61 (dd, *J* = 8.7, 2.0 Hz, 1H), 7.46 (d, *J* = 8.4 Hz, 1H), 3.47 (s, 4H), 3.10 (s, 2H), 2.81 (t, *J* = 7.6 Hz, 2H), 1.72 (p, *J* = 7.3 Hz, 2H), 1.39–1.23 (m, 14H), 0.93–0.85 (m, 3H). ^13^C NMR (101 MHz, CD_3_OD): δ 159.4, 147.2, 142.5, 137.7, 132.5, 128.8, 128.4, 127.2, 124.7, 66.5, 58.8, 43.2, 36.7, 33.1, 32.44 30.7, 30.6, 30.4, 30.3, 23.7, 14.4. HRMS-ESI (*m*/*z*): calcd for C_23_H_37_N_2_O_2_ [M + H]^+^ 373.2855, found: 373.2858.

## Biology

### Bacterial Strains and Culture Conditions

*S. aureus* ATCC 25923 was provided by the Faculty of Pharmacy of the University of Helsinki (Helsinki, Finland). *S. aureus* ATCC 12598 was kindly provided by Dr. Per Saris from the Antimicrobials, probiotics and fermented food laboratory, Faculty of Agriculture and Forestry, University of Helsinki (Helsinki, Finland). *S. aureus* P2 was isolated from a hip prosthetic implant at the Hospital Fundación Jiménez Díaz (Madrid, Spain) and was kindly given by Dr. Ramón Pérez-Tanoira. *A. baumannii* NCTC 13423 and *C. violaceum* NCTC 13278 (Tn5-mutant CV026) were bought from the National Collection of Type Culture (NCTC) (Salisbury, United Kingdom). *P. aeruginosa* ATCC 9027 and *C. violaceum* ATCC 31532 were bought from the American Type Culture Collection (ATCC) (Wesel, Germany). At the start of all experiments, every strain (except for *P. aeruginosa* and *C. violaceum*) was first grown on tryptic soy agar (TSA) (Lab M Ltd., Heywood, U.K.) overnight at 37 °C under aerobic conditions. Then, the colonies were suspended in 5 mL of tryptic soy broth (TSB) (Lab M Ltd., Heywood, U.K.) in a 50 mL Falcon tube (Greiner Bio-One, Kremsmünster, Austria) and grown to exponential phase under aerobic conditions at 37 °C with shaking (220 rpm), until the culture reached a concentration of approximately 1 × 10^8^ colony-forming unit (CFU) mL^–1^. The culture was diluted to 1 × 10^6^ CFU mL^–1^ before starting the experiment. The same was done with *P. aeruginosa* using Lennox broth (LB) and LB-agar (LBA) (Lab M Ltd., Heywood, U.K.). For the QS inhibition analysis, *C. violaceum* was grown on LBA overnight at 27 °C, and the colonies were used to start the experiment directly.

### Compounds

Fingolimod, with purity ≥98%, was purchased from Carbosynth (Compton, U.K.). Fingolimod was dissolved into dimethyl sulfoxide (DMSO) and sonicated for 5 min using a Branson 3800 ultrasonic bath (Cleanosonic, Richmond, VA) at 40 Hz.

### Antibacterial Activity Evaluation

The antimicrobial activity was assessed in a similar way for the initial screening and the dose–response evaluation against different bacterial strains. With the exception of the post-exposure experiment, the compounds were added to the wells along with the bacteria to test the ability of compounds to prevent bacterial growth and biofilm formation (pre-exposure). The compounds were diluted in DMSO and plated directly in 96-well plates before adding the bacterial culture, and plates were incubated for 18 h under aerobic conditions at 37 °C with shaking (220 rpm). The antibacterial activity of the compounds was assessed on planktonic cells and biofilms separately using two different measurements for each: optical density (turbidity) and resazurin reduction (viability) on planktonic cells and resazurin followed by a crystal violet staining assay (total biomass) on biofilms, as described in the following sections.

For the post-exposure experiment, bacteria were grown with no compounds in the same conditions for 18 h, after which the media was changed, and compounds were added on the preformed biofilms. The plates were then incubated in the same conditions for an additional 24 h before proceeding to the staining assays.

### Resazurin Staining

The resazurin staining was performed according to the protocol previously optimized with a few modifications.^46^ Briefly, the planktonic solution was first transferred into a new 96-well plate and the OD of the planktonic solution was measured using a Varioskan LUX Multimode microplate reader (Thermo Scientific, Vantaa, Finland). Then, 10 μL of a 400 μM resazurin (R7017, Sigma-Aldrich, St. Louis, MO) diluted in phosphate-buffered saline (PBS) (Thermo Fisher Scientific, MA) was added to the wells (for a final resazurin concentration of 20 μM). The plate was incubated in the dark with shaking (220 rpm) for about 3–10 min at room temperature with *S. aureus* and *A. baumannii* (until the bacteria controls turned pink) or for 20 min at 37 °C with *P. aeruginosa*. Fluorescence was measured at λ_ex_ = 560 nm and λ_em_ = 590 nm using the top optics of the Varioskan LUX Multimode microplate reader. The planktonic plate was then discarded. The original challenge plate containing the biofilms was washed once with PBS, and 200 μL of a 20 μM resazurin solution was added to the wells. The plate was incubated in the dark at 37 °C with shaking (220 rpm) for 30 min with *S. aureus*, 60 min with *A. baumannii*, and 90 min with *P. aeruginosa*. Fluorescence was measured as described for the planktonic solution. The biofilms were next treated with crystal violet staining.

### Crystal Violet Staining

After removing the resazurin solution from the plate, biofilms were fixed with ethanol 100% for 15 min at room temperature. Ethanol was then removed, and the biofilms were left to air-dry completely at RT. Crystal violet, diluted to 1:100 in deionized water from the 2.3% commercial solution (HT90132, Sigma-Aldrich, St. Louis, MO), was added to the wells and, after a 5 min incubation with the dye, the biofilms were washed twice with deionized water and left to air-dry for about 10 min. The dye was solubilized in ethanol 100% for 1 h, and absorbance was measured at 595 nm using a Multiskan Sky microplate spectrophotometer (Thermo Scientific, Vantaa, Finland).

### Quorum Sensing Inhibition Assay

The QS inhibitory activity of the compounds was assessed as described previously.^31,47^ Briefly, two QS reporter strains, *C. violaceum* ATCC 31532 and the violacein-negative, mini-Tn5 mutant of *C. violaceum* CV026 (NCTC 13278), were grown on LBA (for CV026, the agar was supplemented with kanamycin at 100 μg mL^–1^) overnight at 27 °C. Colonies were suspended in PDYT (0.5% peptone, 0.3% d-glucose, 0.25% yeast extract, 0.05% l-tryptophan, w/v) to reach an OD600 of 0.02. The CV026 culture was supplemented with C6-HSL (*N*-hexanoyl-l-homoserine lactone) (10007896, Cayman Chemical, MI) at 0.5 μM to induce the QS-moderated synthesis of violacein. For each strain, 200 μL of bacterial culture was added per well in two identical 96-well plates containing the compounds (2 μL per well for a final DMSO concentration of 1%). In each 96-well plate, untreated cells were used as negative controls and quercetin (Q4951, Sigma-Aldrich) and azithromycin (PZ0007, Sigma-Aldrich) were included as positive controls for QS inhibition and cell viability (bactericidal activity), respectively. The plates were incubated at 27 °C under aerobic conditions (200 rpm) for 22 h.

The synthesized and nonsoluble violacein was collected from the first 96-well plate by centrifugation (4000 rpm, for 15 min, 20 °C), and the supernatants were removed. Violacein was dissolved in 100 μL per well of 96% (v/v) ethanol and was separated from cells by centrifugation as described above. The supernatant containing violacein was then transferred to a new 96-well plate, and the absorbance was measured at 595 nm using a Multiskan Sky Microplate spectrophotometer. To detect any bactericidal effects, the viability of the cells was measured in the second replica plate by adding 10 μL of 400 μM resazurin in each well and incubating the plate for 30 min in the dark at 27 °C (220 rpm). After centrifugation of the plate to separate the cells from the solution, 100 μL of each well were transferred into a new 96-well plate and the fluorescence was recorded as described above using the Varioskan LUX multimode plate reader.

### Culture of Human Cells

The human lung adenocarcinoma epithelial cells A549, the cervical adenocarcinoma HeLa-derived human epithelial type 2 (Hep-2) cells, and the HL-60 cells were all purchased from the American Type Culture Collection (ATCC, Wesel, Germany). The A549 cells were grown in Dulbecco’s modified Eagle’s medium (DMEM) (BE12-707F, Lonza, Basel, Switzerland) supplemented with 10% heat-inactivated fetal bovine serum (FBS) (Sigma-Aldrich, St. Louis, MO), 2 mM glutamine (Sigma-Aldrich, St. Louis, MO), and 20 μg mL^–1^ gentamycin (Sigma-Aldrich, St. Louis, MO). The HEp-2 cells were grown in DMEM (41966-029, Sigma-Aldrich, St. Louis, MO), supplemented with 10% (v/v) heat-inactivated FBS and 20 μg mL^–1^ gentamycin. Both cell lines were maintained at 37 °C in 5% CO_2_ in a humidified incubator. HL-60 cells were grown and maintained in Roswell Park Memorial Institute (RPMI) 1640 Medium (R8758, Sigma-Aldrich, St. Louis, MO), supplemented with 10% (v/v) heat-inactivated FBS and 1% (v/v) penicillin/streptomycin (Sigma-Aldrich, St. Louis, MO). The cells were maintained at 37 °C in 5% CO_2_ in a humidified incubator. Before each experiment, the HL-60 cells were differentiated into neutrophile-like cells by culturing the cells for 6 days in the maintenance medium supplemented with 100 mM of *N*,*N*–dimethylformamide (DMF) (Sigma-Aldrich, St. Louis, MO).^48^

### Cytotoxicity Assessment

The cytotoxicity of the compounds was assessed on the three different human cell lines, A549, HEp-2, and HL-60, using the redox-based resazurin staining assay. The cells were seeded in clear 96-well plates at a density of 1 × 10^4^ cells per well in a volume of 200 μL per well. The adherent cells were let to attach for 24 h before changing the media and adding the compounds (final concentration of 0.25% DMSO). HL-60 were counted and added to the plates at a density of 1 × 10^4^ cells per well in a volume of 200 μL directly with the compounds. Untreated cells were used as negative controls, and cells treated with 1% TritonX-100 (Sigma-Aldrich, St. Louis, MO) were used as positive controls for cytotoxicity. The cells were incubated with the compounds for 24 h.

For the resazurin assay, the media was first removed, and the adherent cells were washed once with PBS. Then, 200 μL of 20 μM resazurin was added per well and cells were incubated for two more hours before the measurement of fluorescence, as mentioned earlier. For HL-60, 10 μL of 400 μM resazurin was added directly to the wells and the cells were incubated for an additional 4 h before the measurement of fluorescence.

### Effect of Fingolimod on *S. aureus* in Coculture with Differentiated HL-60 Cells

The coculture system was as previously described.^40^ Briefly, the bacteria were prepared as described above and the concentration was adjusted to 2 × 10^7^ CFU mL^–1^ in RPMI 1640. The media of the differentiated HL-60 cells was refreshed with RPMI 1640 24 h before the experiments to remove possible traces of antibiotics. On the day of the experiment, the cells were counted and adjusted to a concentration of 2 × 10^5^ cells mL^–1^ in RPMI 1640. Each suspension (bacterial and differentiated HL-60 cells, 500 μL of each) was added on 1-cm-long sections of a sterilized fine bore LDPE tubing (Smiths Medical ASD, Minneapolis, MN) in 24-well plates (Nunclon Δ surface, Nunc, Roskilde, Denmark). Fingolimod was also added to the wells at a concentration of 25 μM (0.25% DMSO). Coculture controls with no added compound were included, as well as bacterial controls without differentiated HL-60 cells with and without fingolimod. The plates were incubated for 24 h in the same conditions as for cell maintenance. Then, the LDPE tubes were gently washed with TSB to remove adhering planktonic cells and transferred to Eppendorf tubes containing 1 mL of 0.5% (w/v) Tween 20-TSB solution. Next, the tubes were sonicated in a water bath sonicator (Branson Ultrasonics, Danbury, CT) for 5 min at 35 kHz. The tubes were vortexed for 20 s prior to and after the sonication step. Serial dilutions of the resulting bacterial suspensions were made from 10^–1^ up to 10^–7^ and plated on TSA plates.

### Statistical Analysis

In the QS inhibition assay, statistical difference between the test compounds was calculated at each concentration using a one-way analysis of variance (ANOVA) with a Welch correction in the case of violation of the homogeneity of variance. A significant difference was observed against the wild-type strain at 10 μM [F(6, 9.26) = 10.71, *p* < 0.001], 15 μM [F(6, 21) = 5.87, *p* = 0.001], and 25 μM [F(6, 8.79) = 9.51, *p* = 0.002] as well as against the mutant strain at 10 μM [F(6, 9.25) = 12.64, *p* < 0.001]. A Dunnett’s post hoc test was used to detect statistical differences between fingolimod and each of its derivatives. In the bacteria-neutrophil coculture assay, significance was determined using unpaired *t*-tests.

## Supporting Information Available

Molecular formula strings (XLSX)
Initial screening of fingolimod derivatives (Figure S1); viability inhibition of fingolimod derivatives against *C. violaceum* (Figure S2); aggregation data for fingolimod, **43**, **45**, **55**, **62**, **79**, and **82** (Figure S3), NMR spectra (Figures S4–S59), additional tables providing inhibition data (Tables S1, S2, and S3) (PDF)

## Abbreviations

AHL: acyl-homoserine lactones
ATP: adenosine triphosphate
CFU: colony-forming unit
DMSO: dimethyl sulfoxide
Fgd: fingolimod
LB: Lennox broth
LBA: Lennox broth agar
MIC: minimum inhibitory concentration
OD: optical density
PBS: phosphate-buffered saline
QS: quorum sensing
QSI: quorum sensing inhibitor
rt: room temperature
TSA: tryptic soy agar
TSB: tryptic soy broth
