# Supplementary material for: Synthesis and Biological Evaluation of Fingolimod Derivatives as Antibacterial Agents
Source: ACS Omega. 2021 Jul 9;6(28):18465–86. doi: 10.1021/acsomega.1c02591 (PMC8296573; doi:10.1021/acsomega.1c02591)
Supplement: Supplementary file 2 — ao1c02591_si_002.pdf [file ao1c02591_si_002.pdf]

Supporting information

Synthesis and Biological Evaluation of Fingolimod Derivatives as Antibacterial Agents

Matej Zore[§,‡], Shella Gilbert-Girard[†,‡], Inés Reigada[†], Jayendra Z. Patel[§], Kirsi Savijoki[†], Adyary Fallarero[†] and Jari Yli-Kauhaluoma[*,§]

[§] Drug Research Program, Division of Pharmaceutical Chemistry and Technology, Faculty of Pharmacy, University of Helsinki, Viikinkaari 5 E, FI-00014 Helsinki, Finland

[†] Drug Research Program, Division of Pharmaceutical Biosciences, Faculty of Pharmacy, University of Helsinki, Viikinkaari 5 E, FI-00014 Helsinki, Finland

Table of contents

1. Initial screening of fingolimod derivatives (Figure S1) 3

2. Viability inhibition of fingolimod derivatives against *C. violaceum* (Figure S2)........................ 3

3. Aggregation data (Figure S3) 4

4. NMR spectra (Figure S4-S59)... 5

5. Inhibition results against *S. aureus* ATCC 25923 (Table S1) 33

6. Inhibition results against the clinical strains of *S. aureus* (Table S2)................................ 37

7. Inhibition results against the gram-negative bacteria (Table S3)....................................... 39

1. Initial screening of fingolimod derivatives (Figure S1)

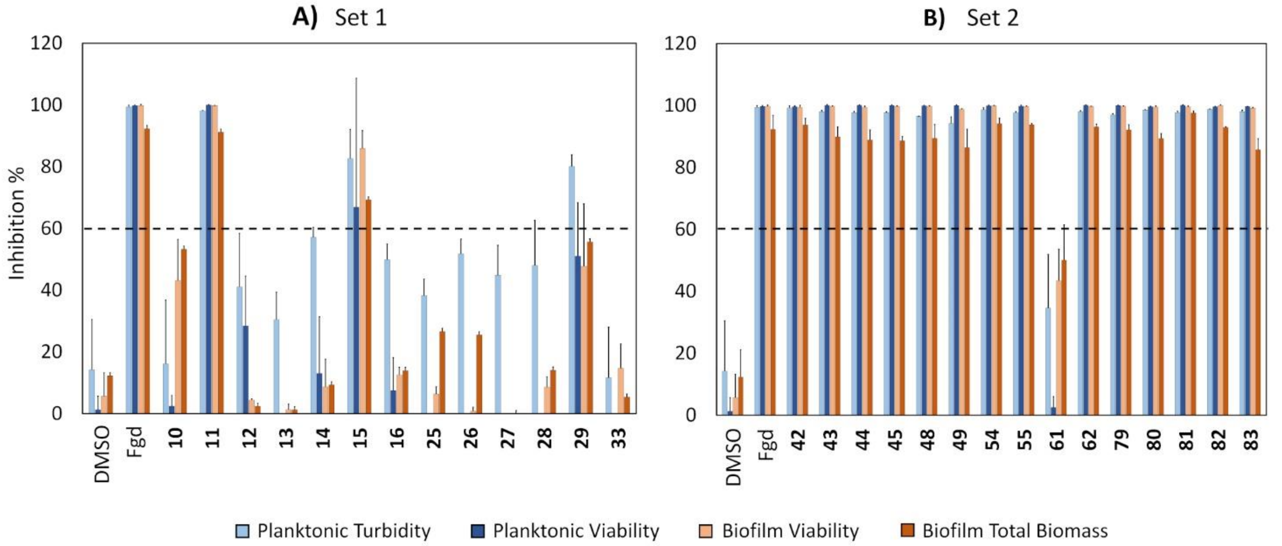

Figure S1. Screening of the two groups (**A)** Set 1 and **B)** Set 2) of fingolimod (Fgd) derivatives at 50 µM against planktonic cells and biofilms of *S. aureus* ATCC 25923. Results are expressed as the inhibition percentage ± SD as compared to the untreated controls. The experiment was repeated twice.

2. Viability inhibition of fingolimod derivatives against *C. violaceum* (Figure S2)

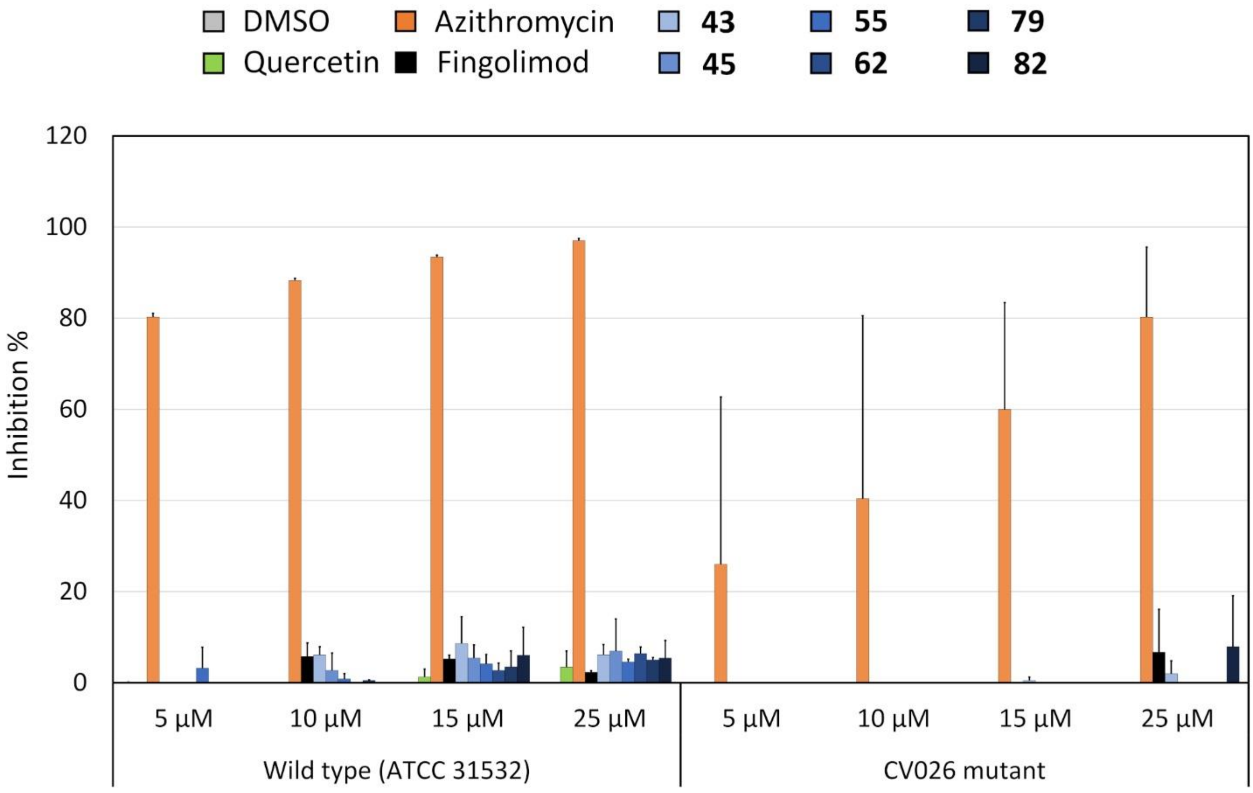

Figure S2. Viability inhibitory activity of selected fingolimod (Fgd) derivatives at various concentrations against *C. violaceum* ATCC 31532 (wild type) and *C. violaceum* CV026 (AHL-deficient mutant). Results are expressed as the inhibition percentage ± SD as compared to the untreated control. The experiment was repeated twice with two replicates per concentration.

3. Aggregation data (Figure S3)

Potential colloidal aggregation in biological assays was studied at five concentrations (5 µM, 10 µM, 15 µM, 25 µM, 50 µM). The original assay conditions were simulated, and the light scattering of potential aggregates in the mixture was studied by nephelometric methods using Nepheloskan Ascent (Labsystems, Finland). Aggregation of the blank and compounds fingolimod, **43**, **45**, **55**, **62**, **79** and **82** were measured as triplicates from one independent experiment at 400 V and at room temperature. All compounds showed aggregate formation at 50 µM. Fingolimod, **43**, **45** and **79** showed potential aggregation also at 25 µM. However, at 15 µM, 10 µM and 5 µM, the obtained values were close to those observed by blank, therefore indicating only minor or no detectable aggregation.

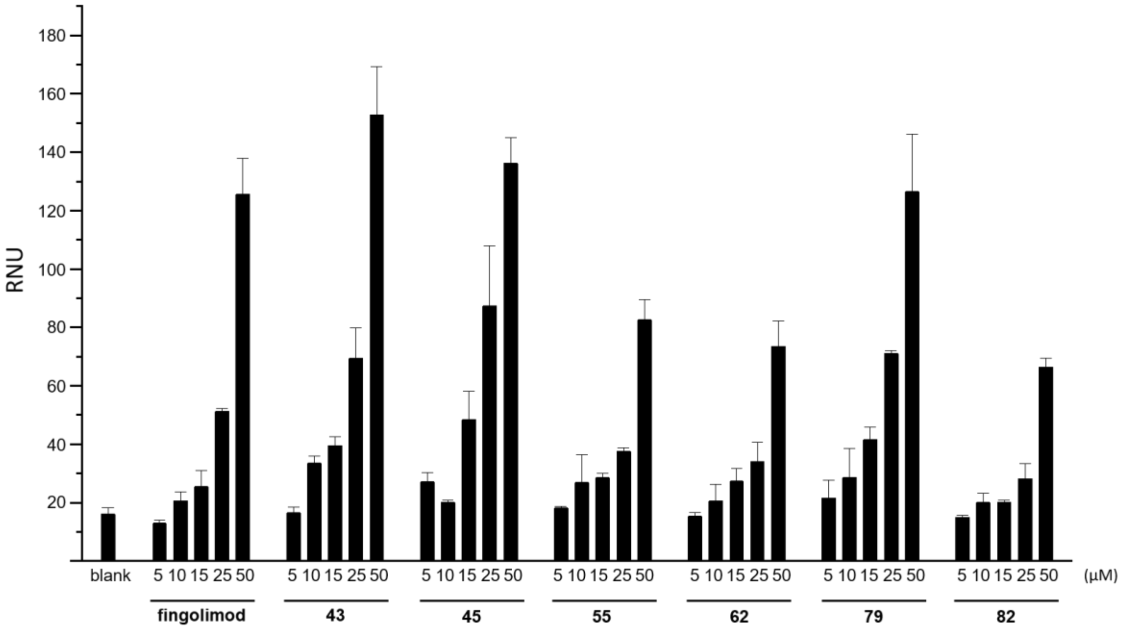

Figure S3: Aggregation data for fingolimod and compounds **43**, **45**, **55**, **62**, **79**, **82**. RNU = relative nephelometric unit. The data are shown as the mean ± SD and normalized to the blank.

4. NMR spectra (Figure S4-S59)

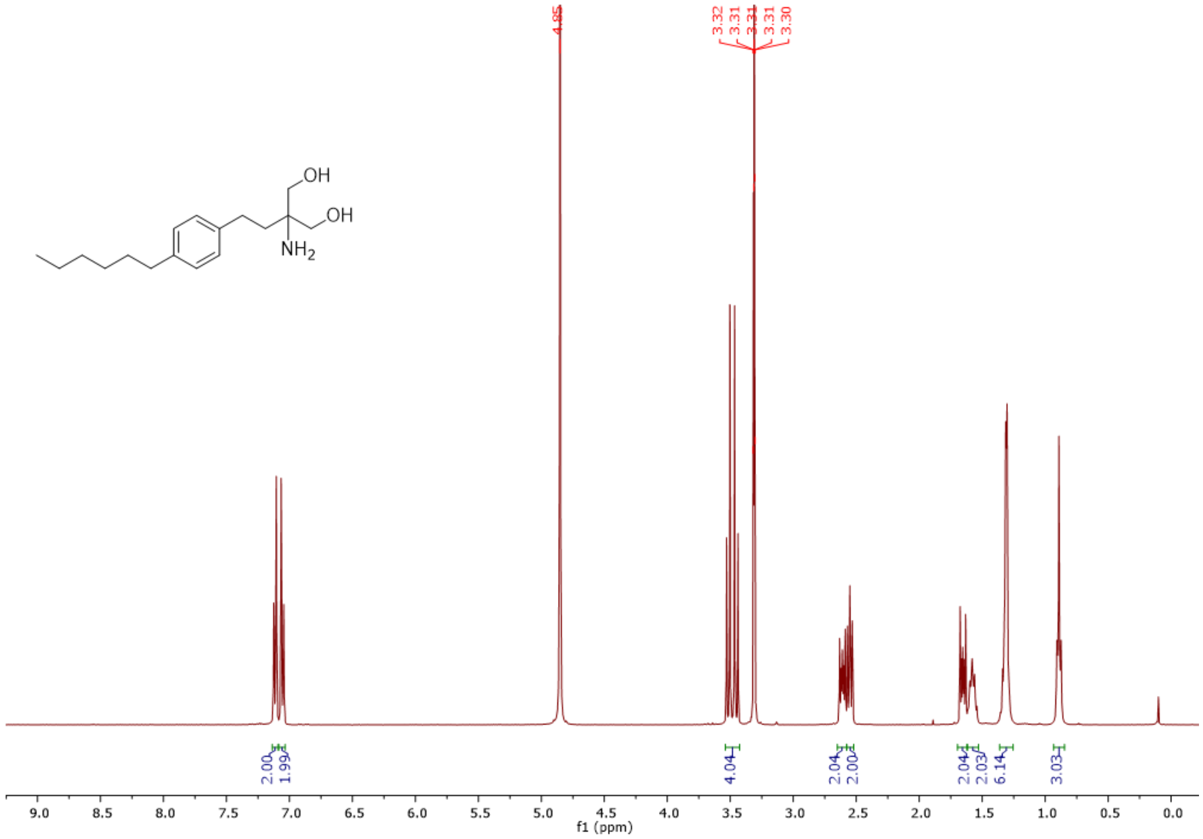

Figure S4: ^{1}H NMR spectra of compound **10** in CD_3OD.

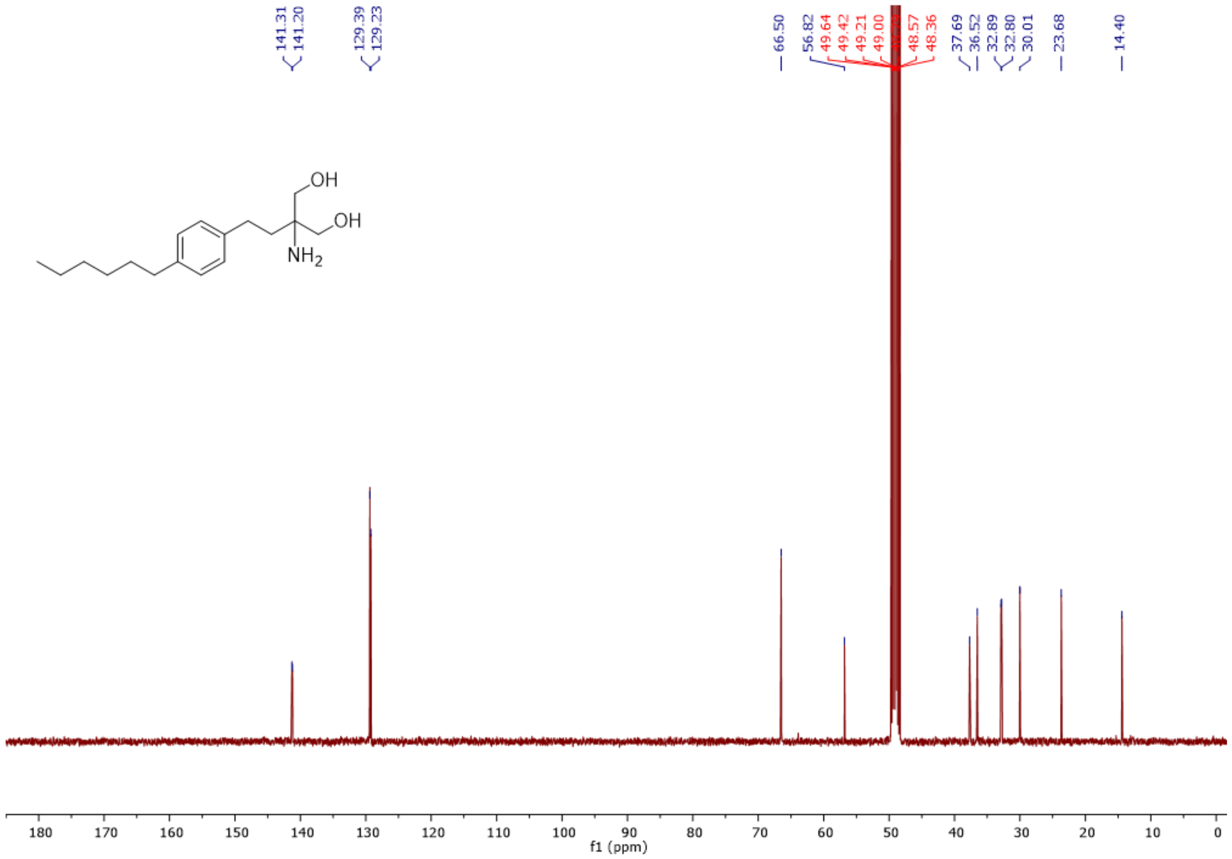

Figure S5: ^{13}C NMR spectra of compound **10** in CD_3OD.

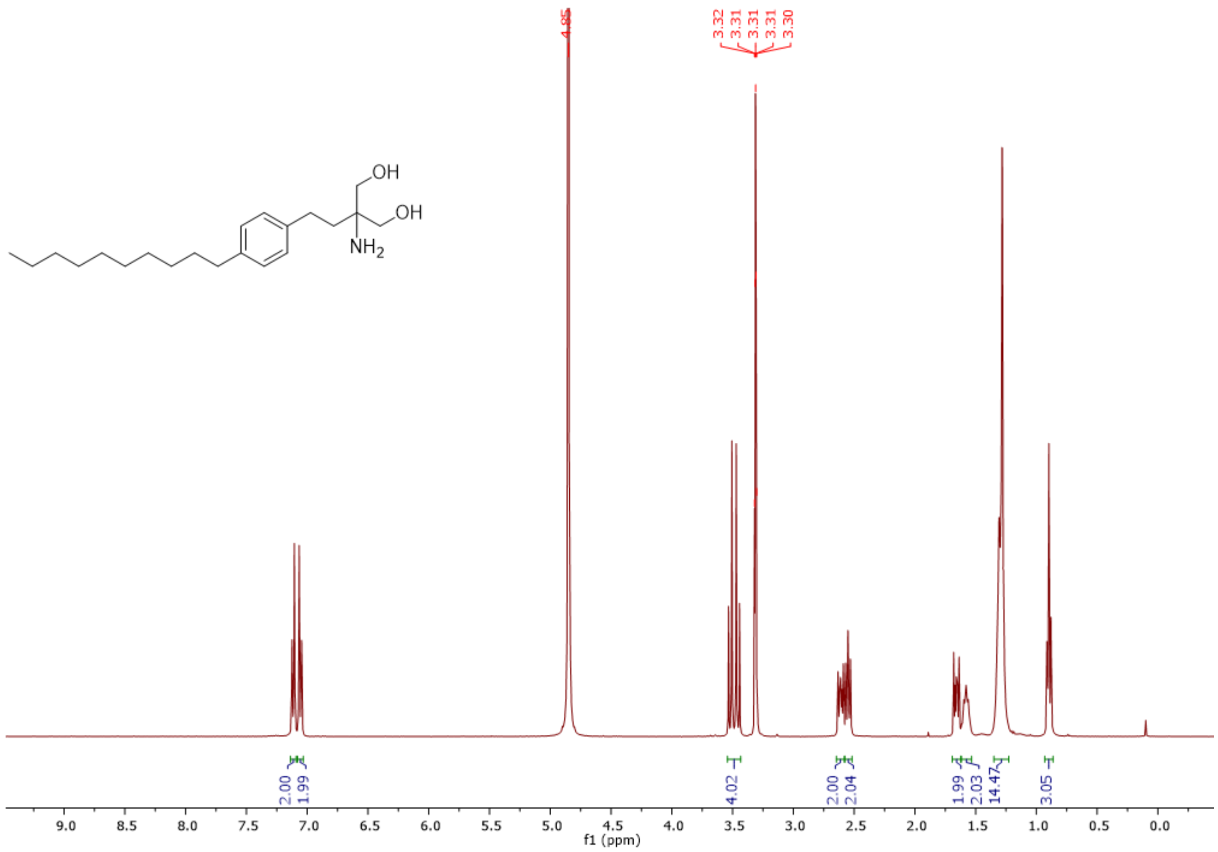

Figure S6: ^{1}H NMR spectra of compound **11** in CD_3OD.

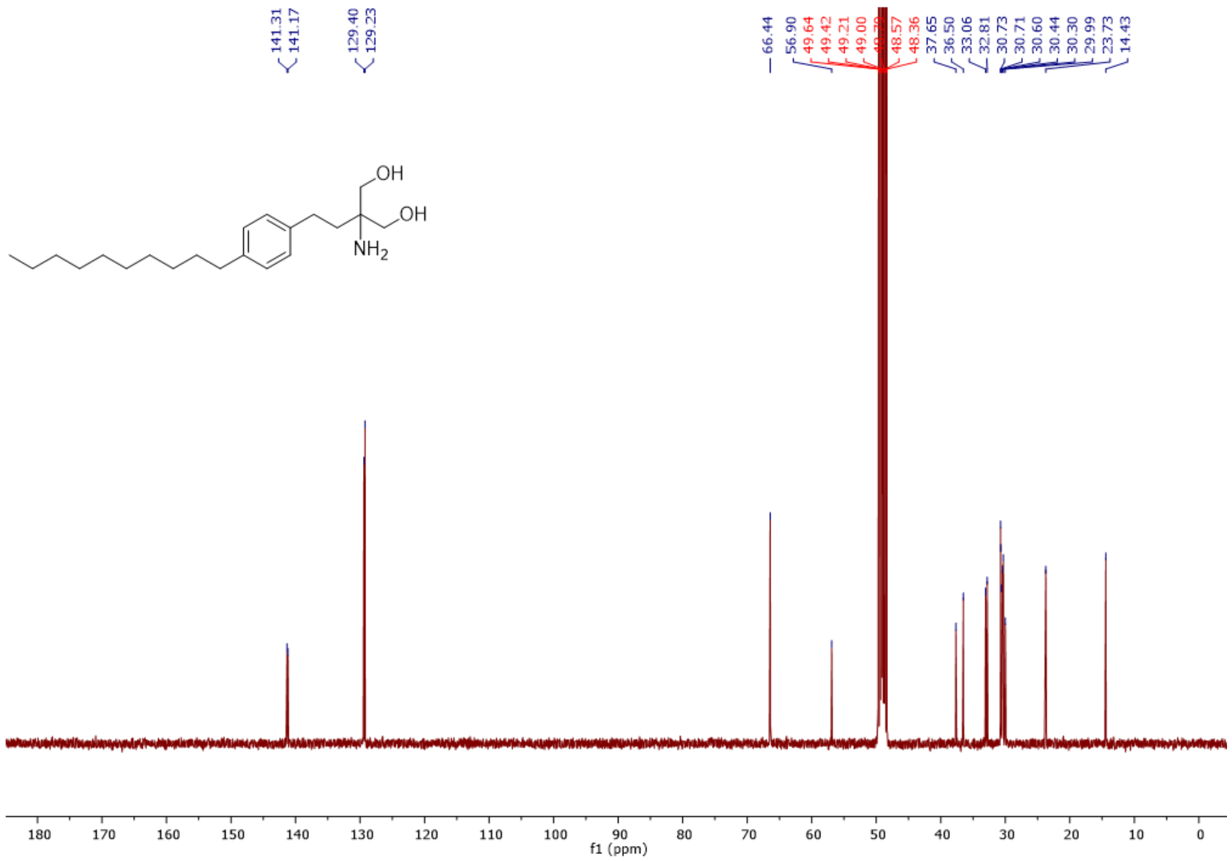

Figure S7: ^{13}C NMR spectra of compound **11** in CD_3OD.

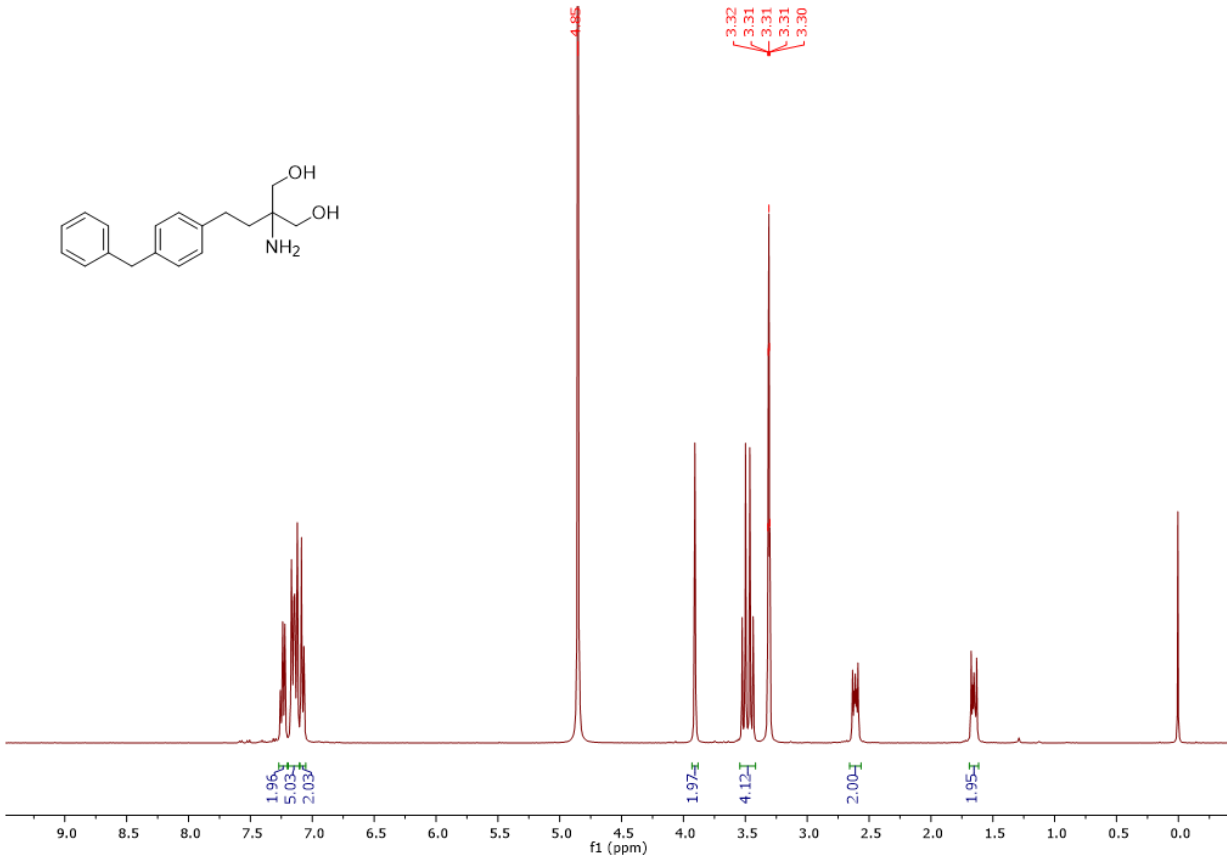

Figure S8: ^{1}H NMR spectra of compound **12** in CD_3OD.

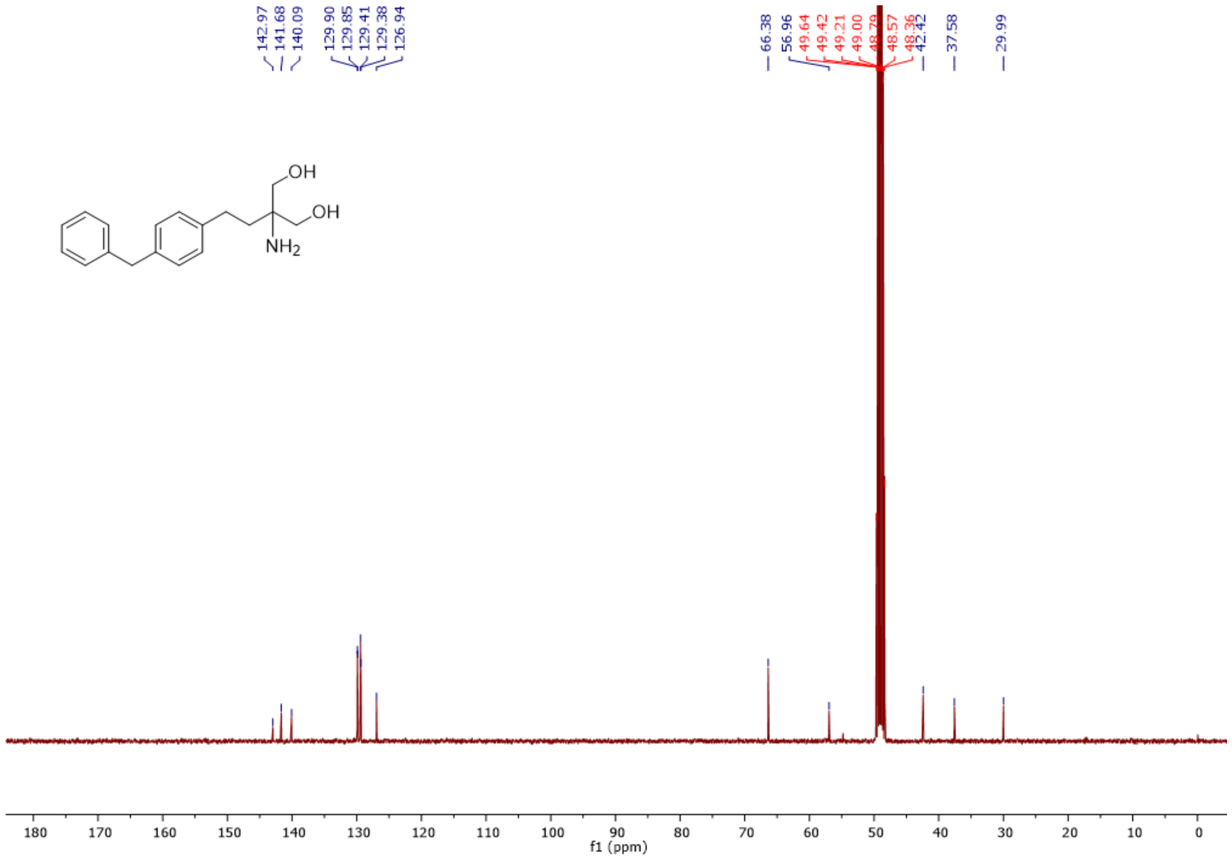

Figure S9: ^{13}C NMR spectra of compound **12** in CD_3OD.

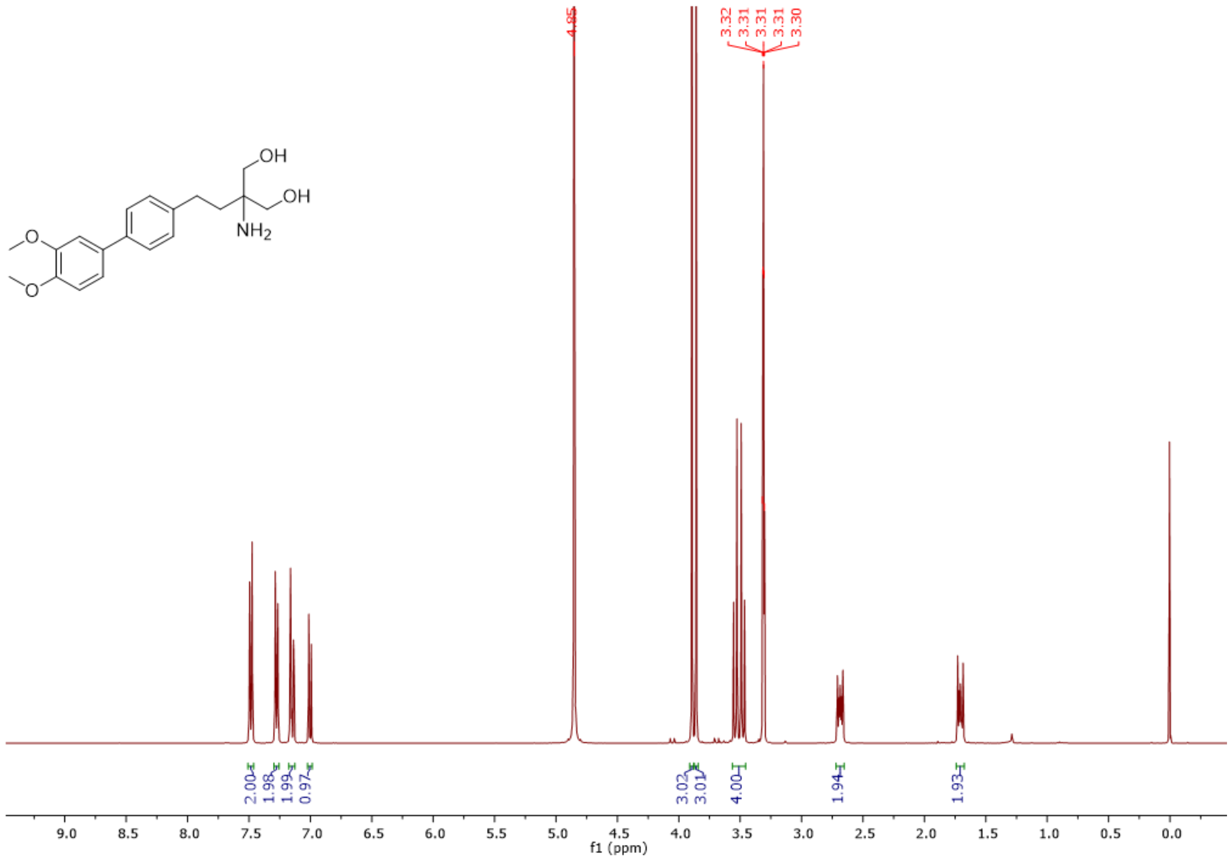

Figure S10: ^{1}H NMR spectra of compound **13** in CD_3OD.

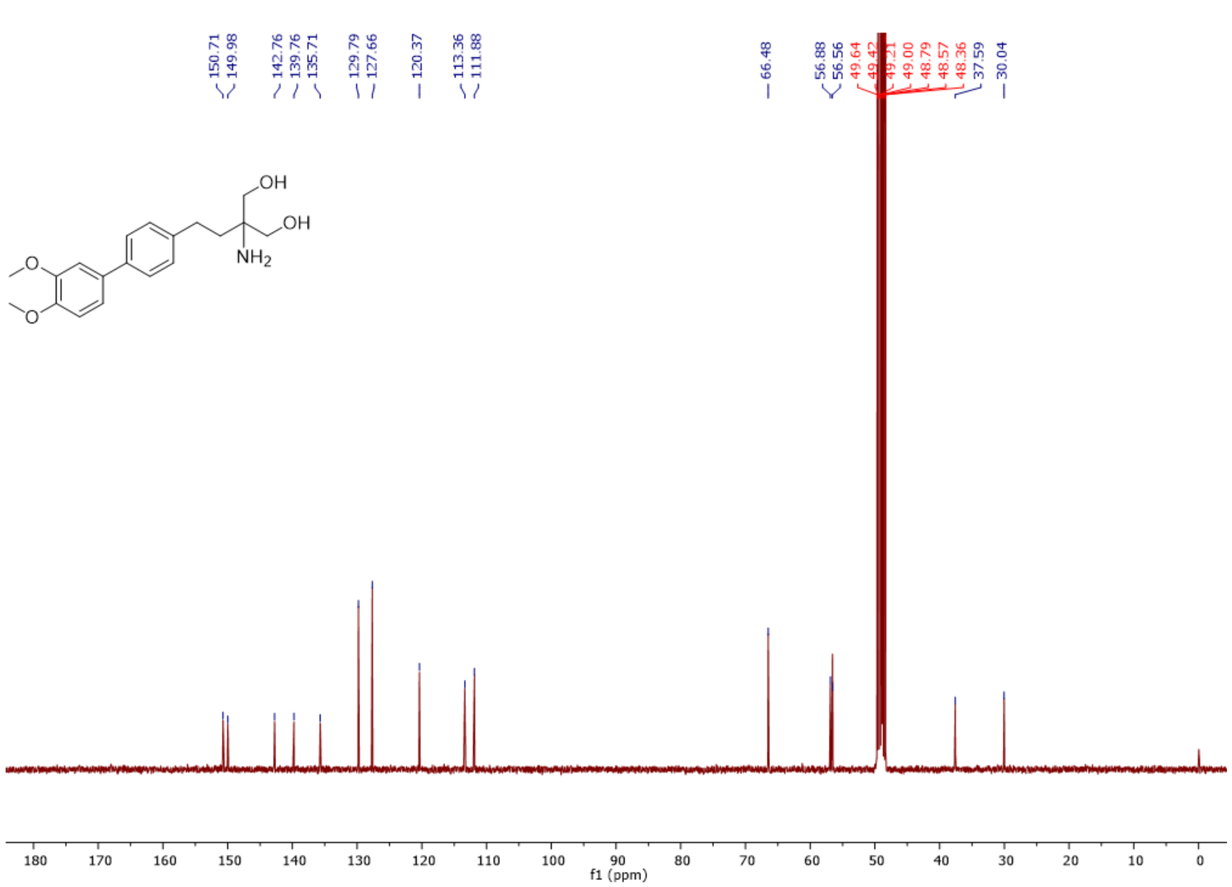

Figure S11: ^{13}C NMR spectra of compound **13** in CD_3OD.

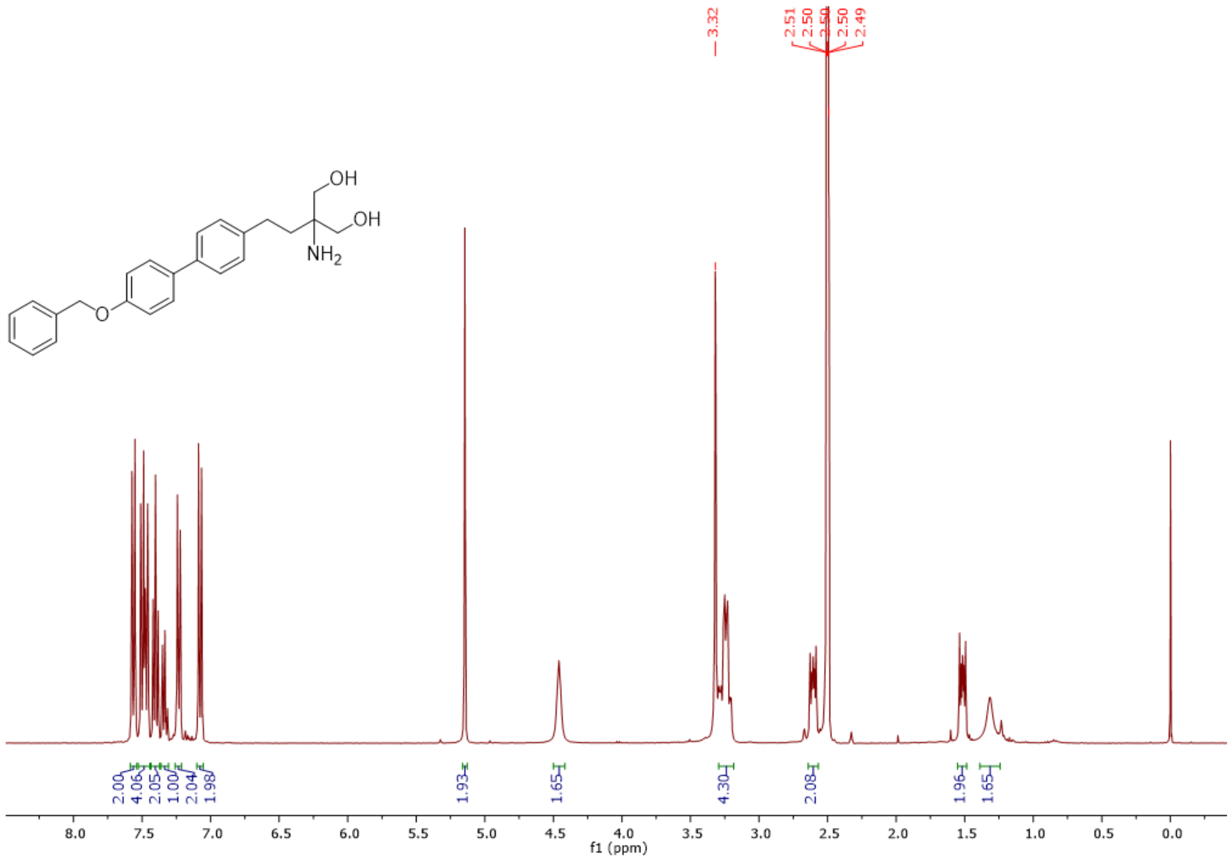

Figure S12: ^{1}H NMR spectra of compound **14** in DMSO-*d*$_6$.

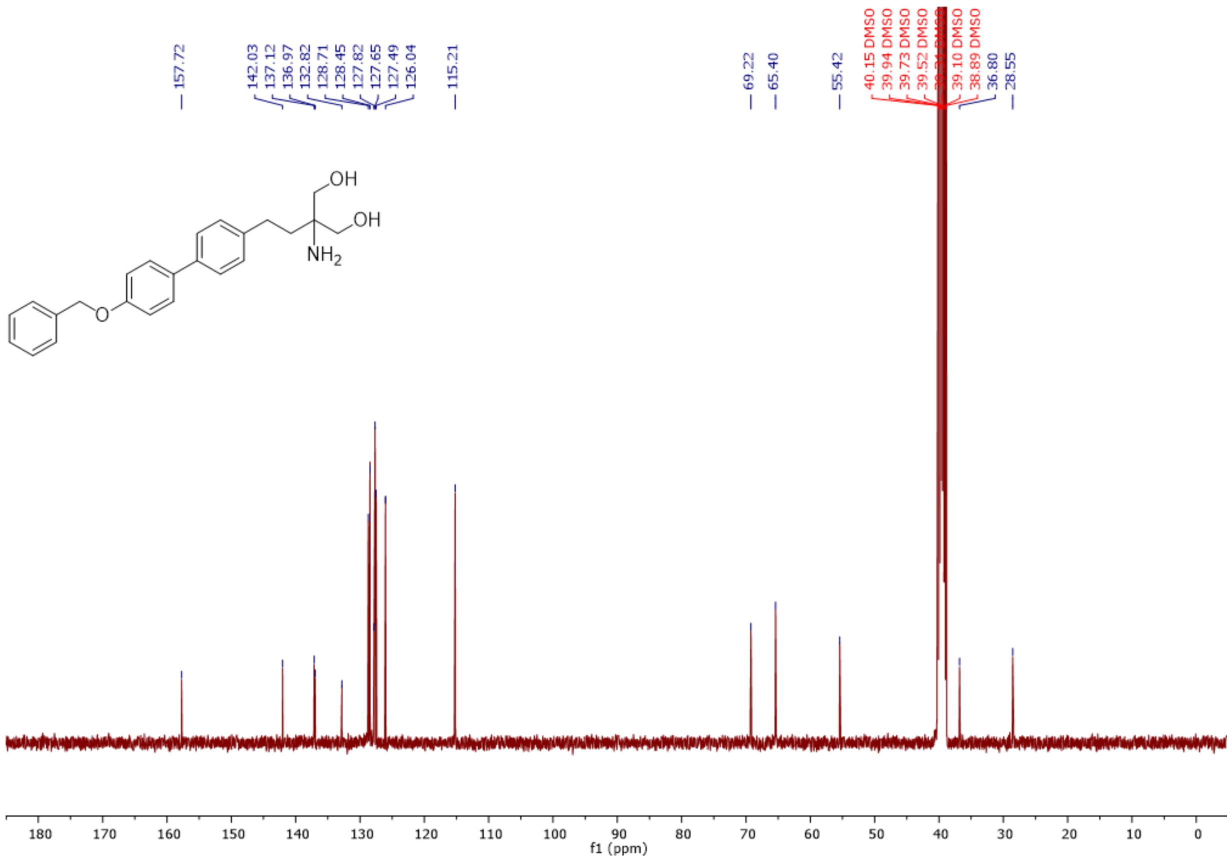

Figure S13: ^{13}C NMR spectra of compound **14** in DMSO-*d*$_6$.

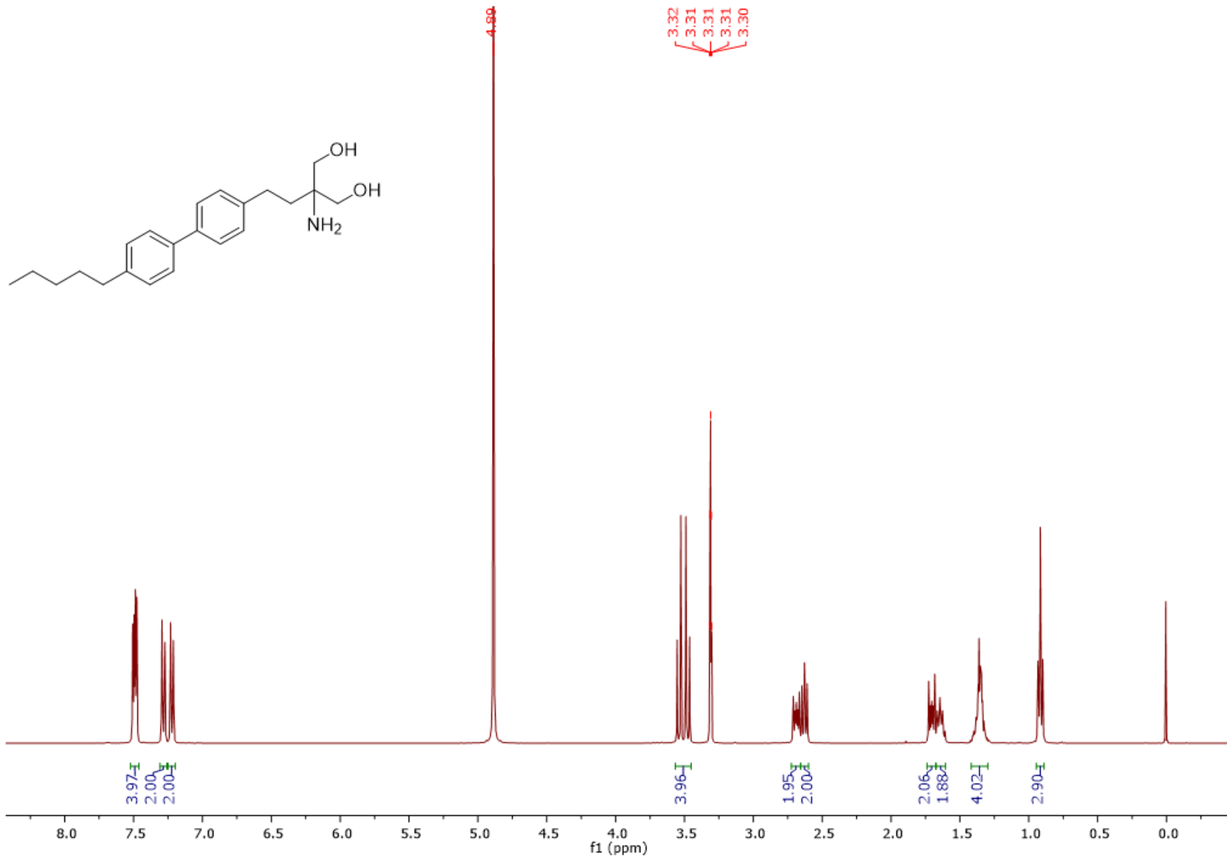

Figure S14: ^{1}H NMR spectra of compound **15** in CD_3OD.

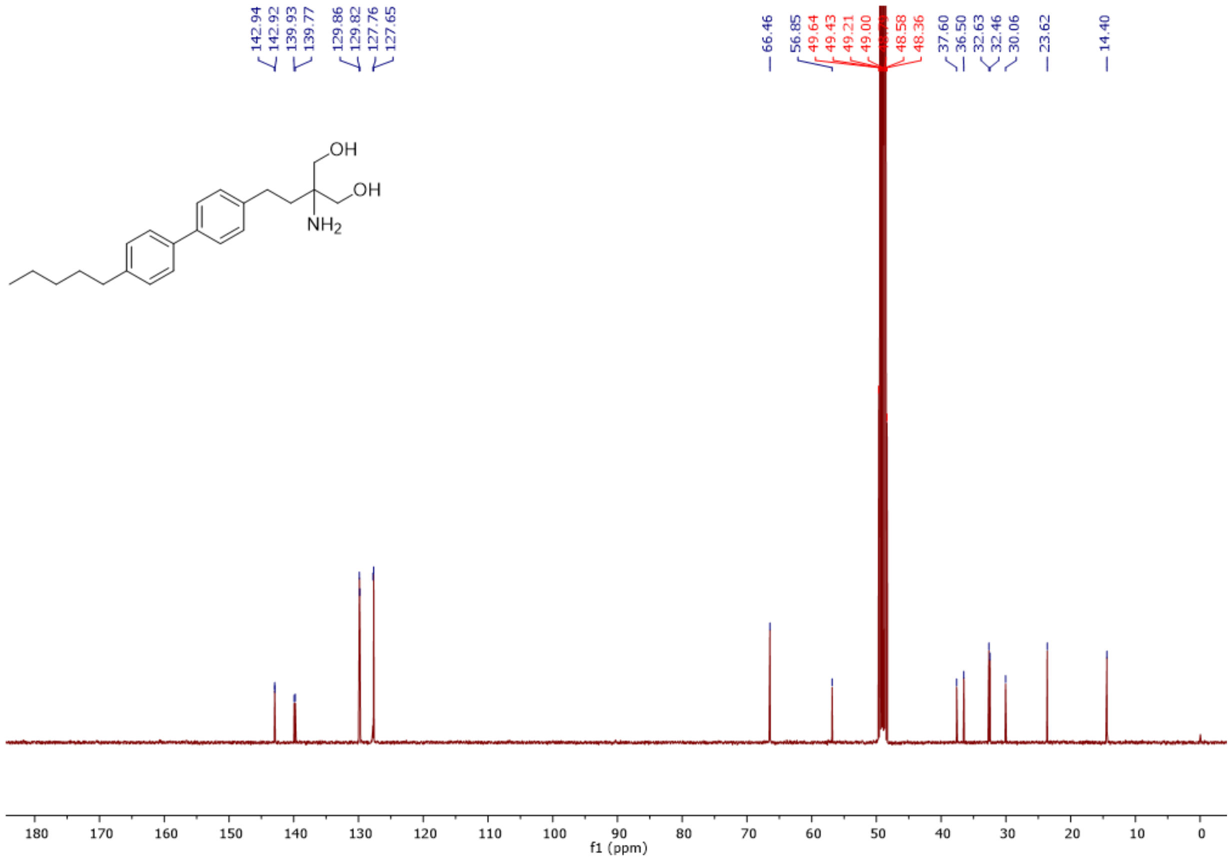

Figure S15: ^{13}C NMR spectra of compound **15** in CD_3OD.

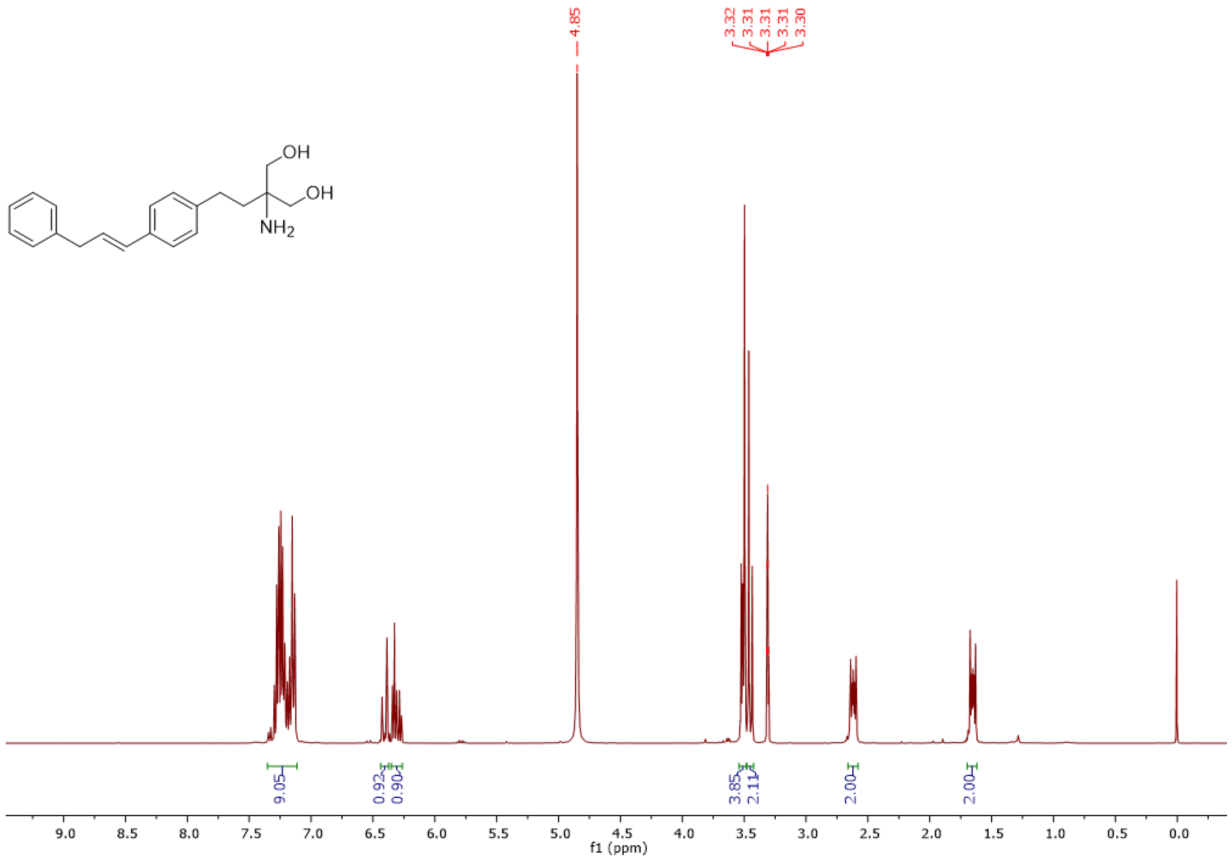

Figure S16: ^{1}H NMR spectra of compound **16** in CD_3OD.

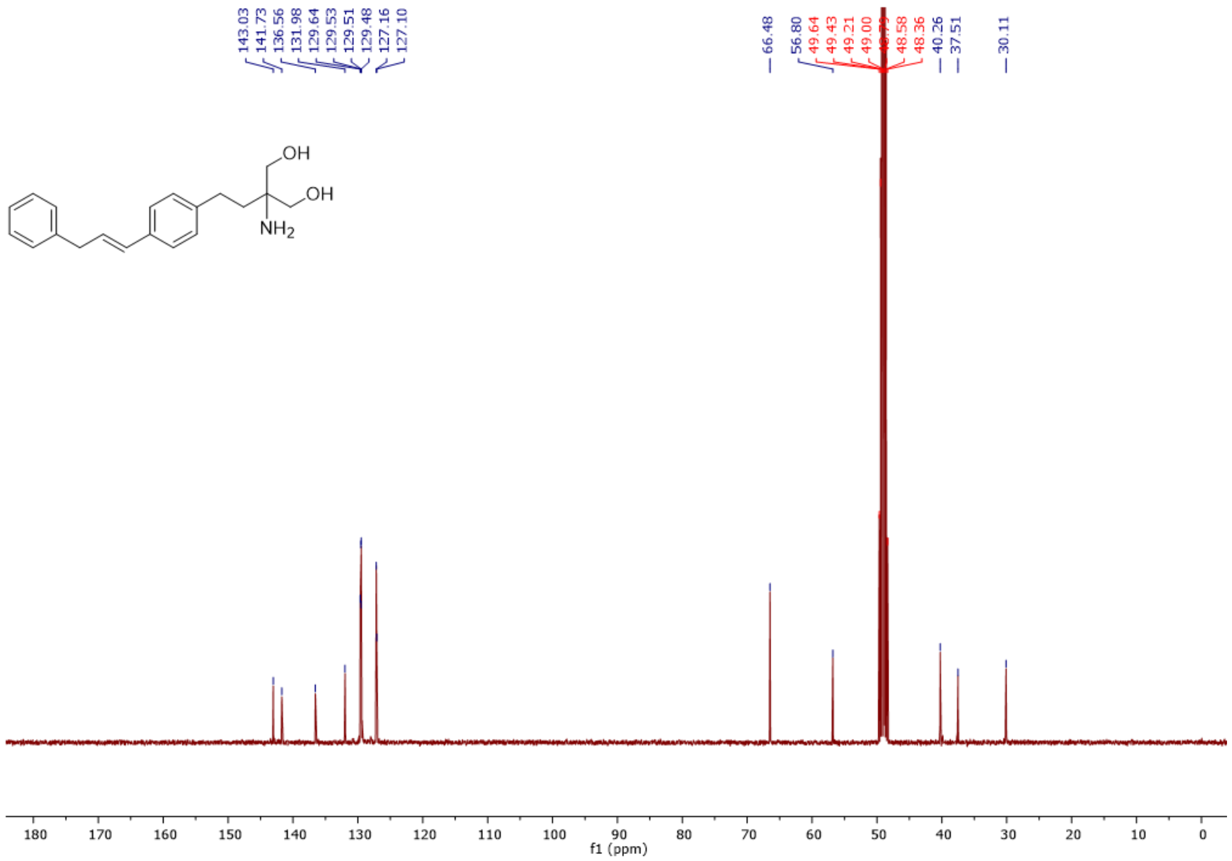

Figure S17: ^{13}C NMR spectra of compound **16** in CD_3OD.

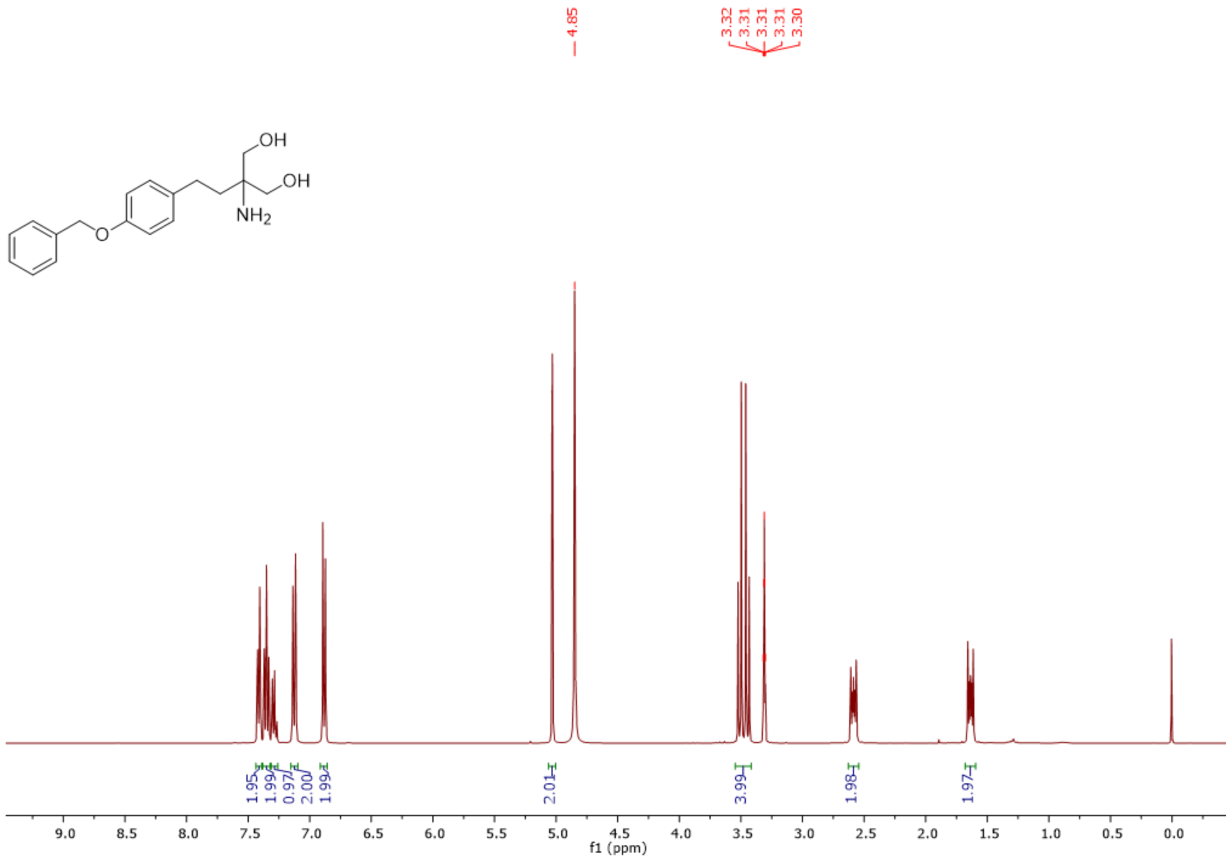

Figure S18: ^{1}H NMR spectra of compound **25** in CD_3OD.

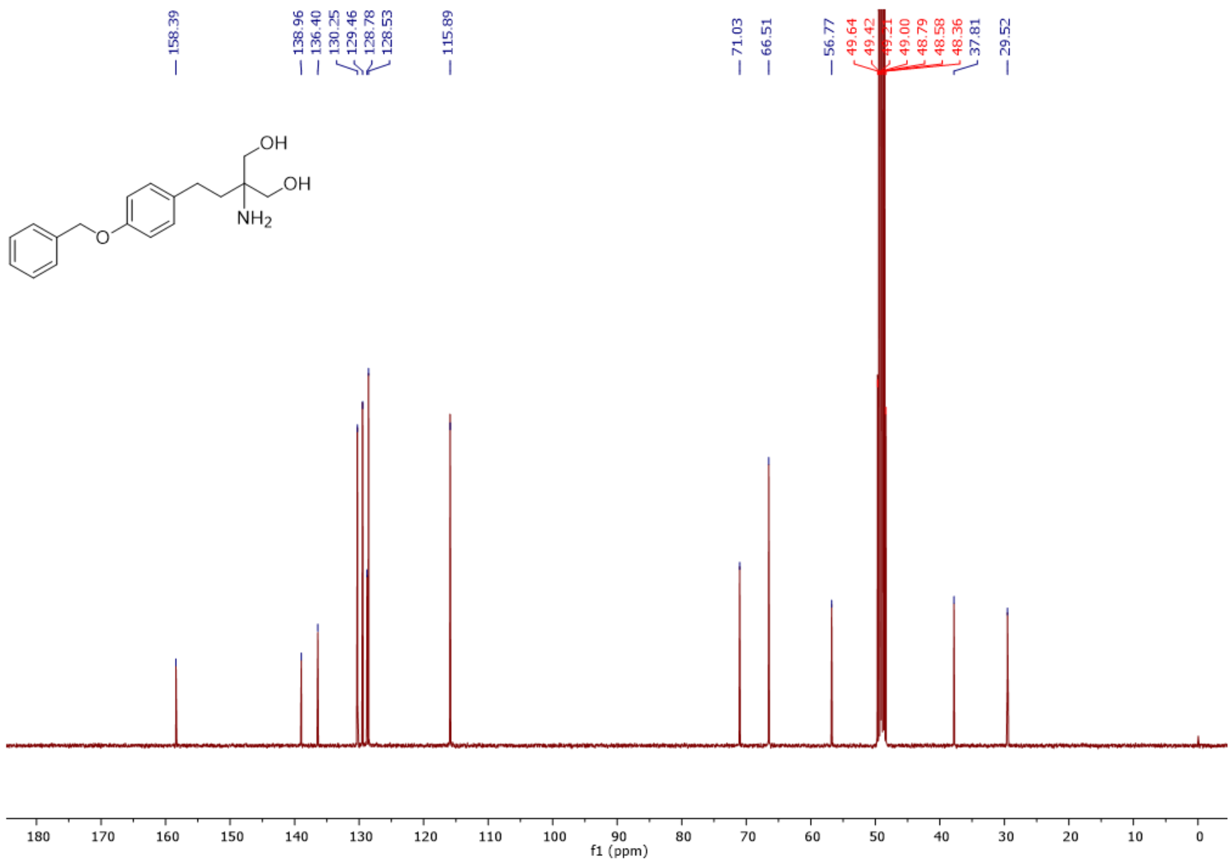

Figure S19: ^{13}C NMR spectra of compound **25** in CD_3OD.

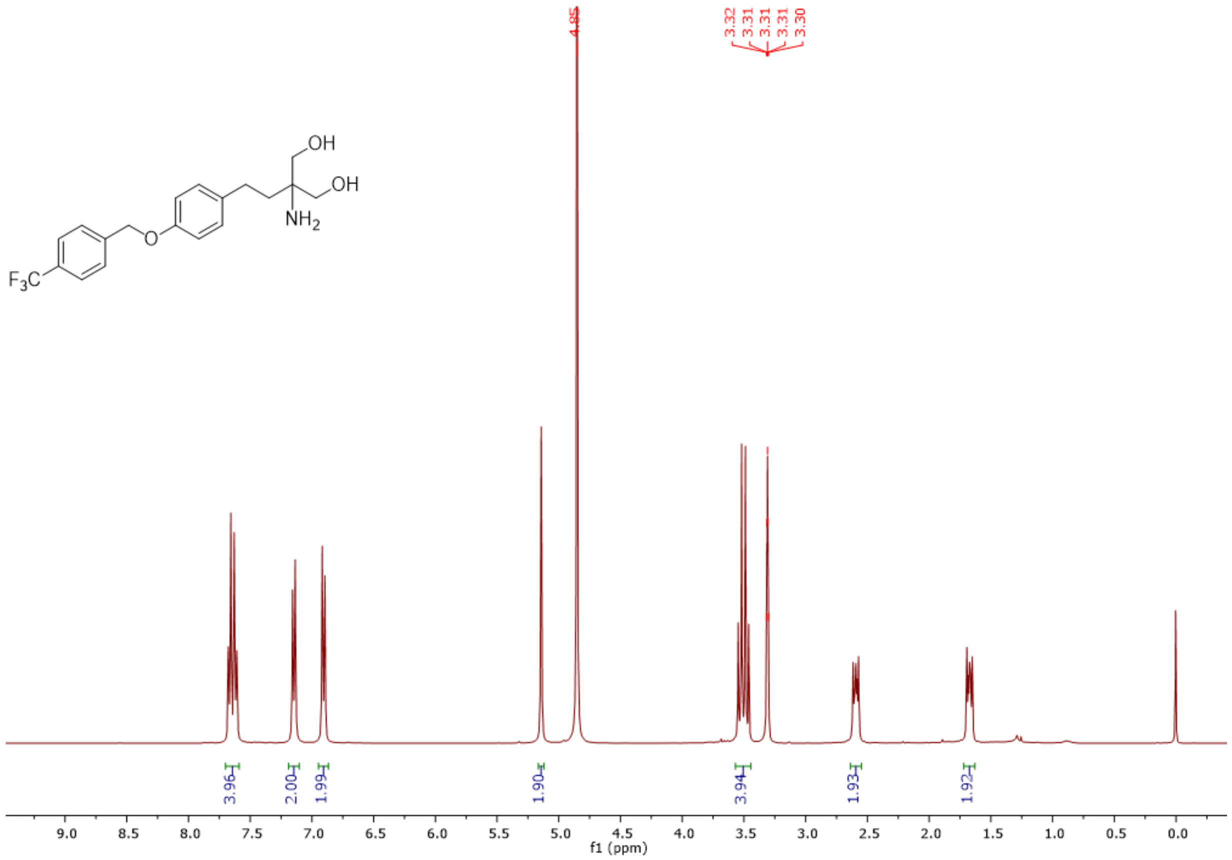

Figure S20: ^{1}H NMR spectra of compound **26** in CD_3OD.

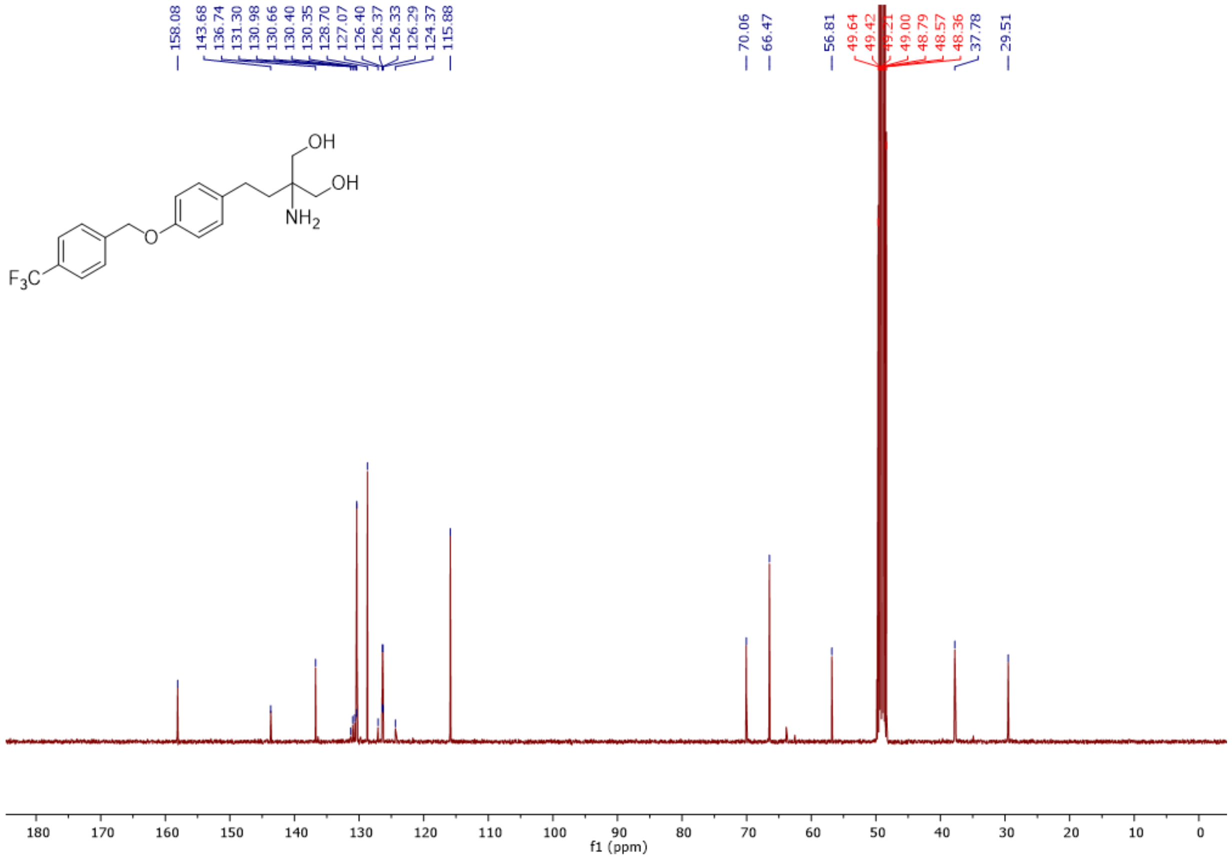

Figure S21: ^{13}C NMR spectra of compound **26** in CD_3OD.

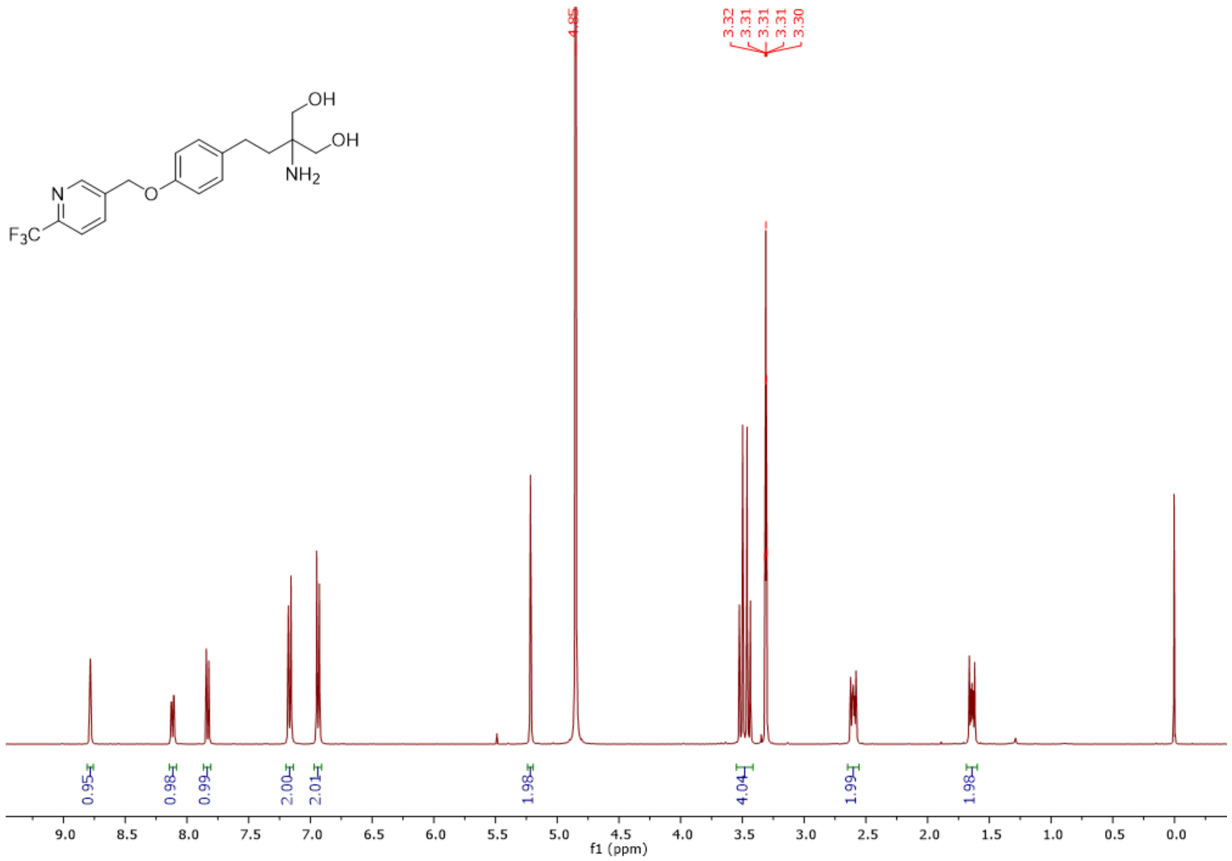

Figure S22: ^{1}H NMR spectra of compound **27** in CD₃OD.

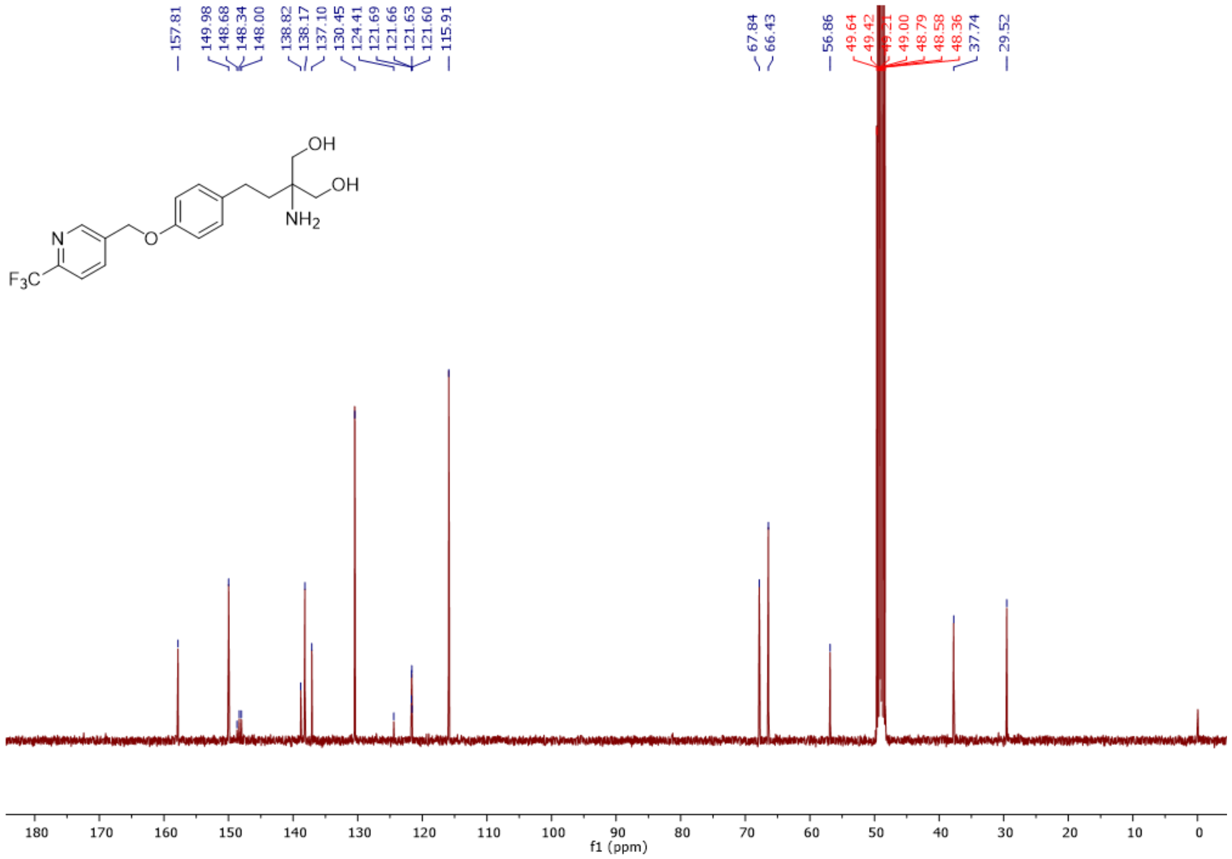

Figure S23: ^{13}C NMR spectra of compound **27** in CD₃OD.

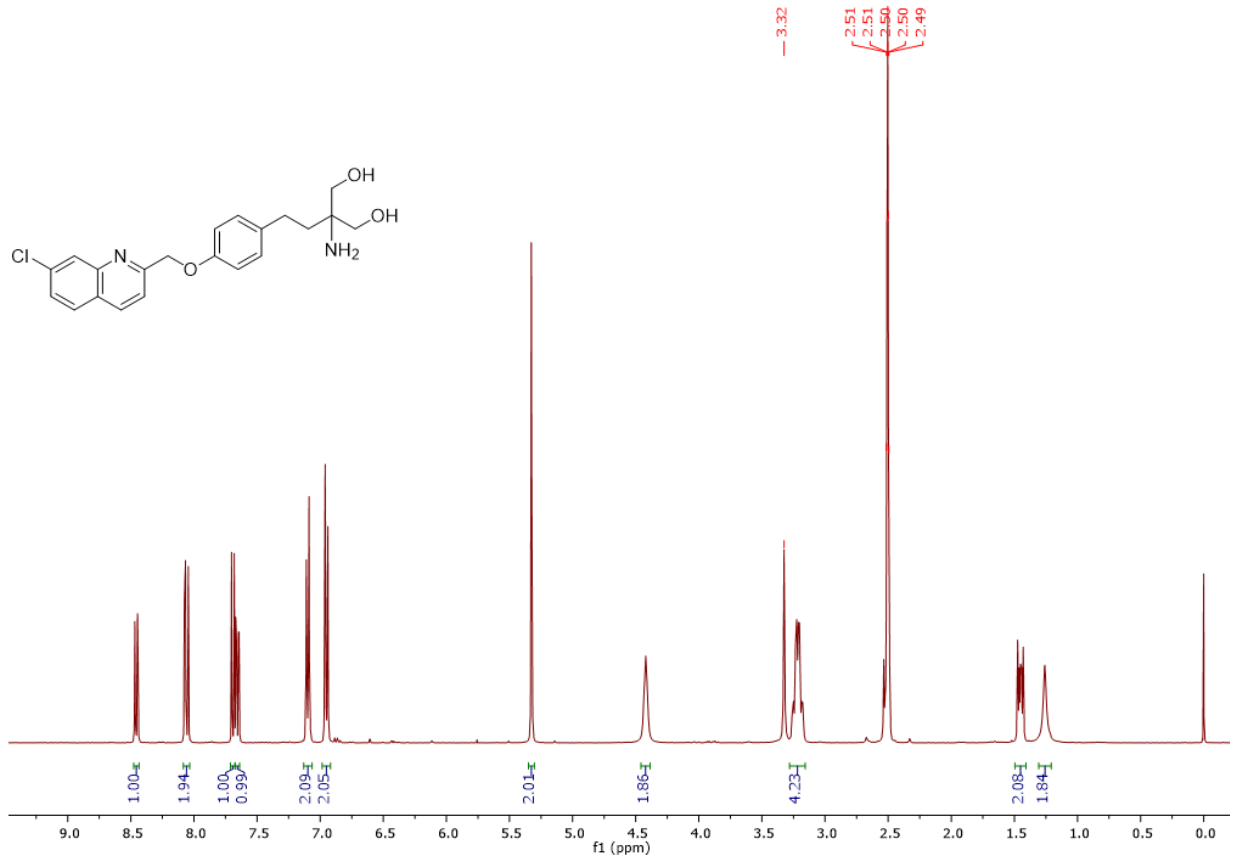

Figure S24: ^{1}H NMR spectra of compound **28** in DMSO-d_6.

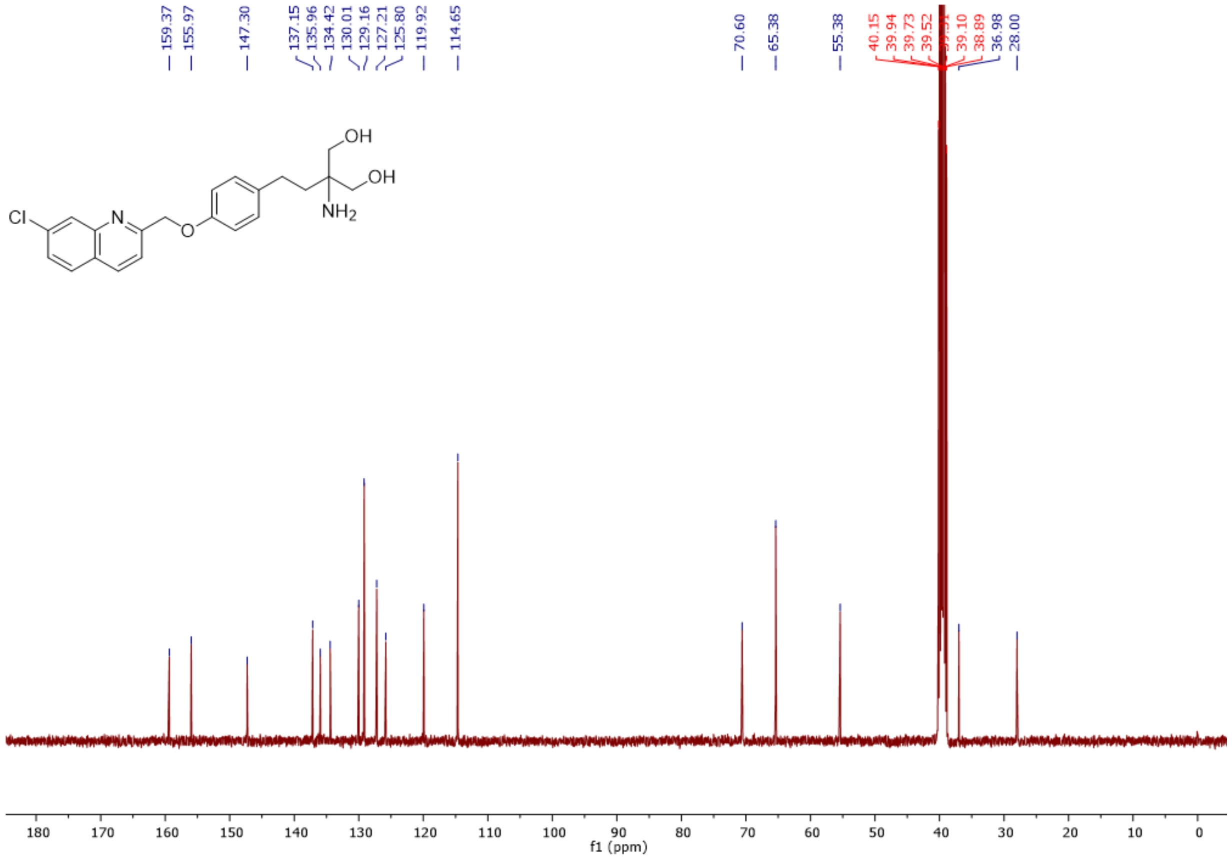

Figure S25: ^{13}C NMR spectra of compound **28** in DMSO-d_6.

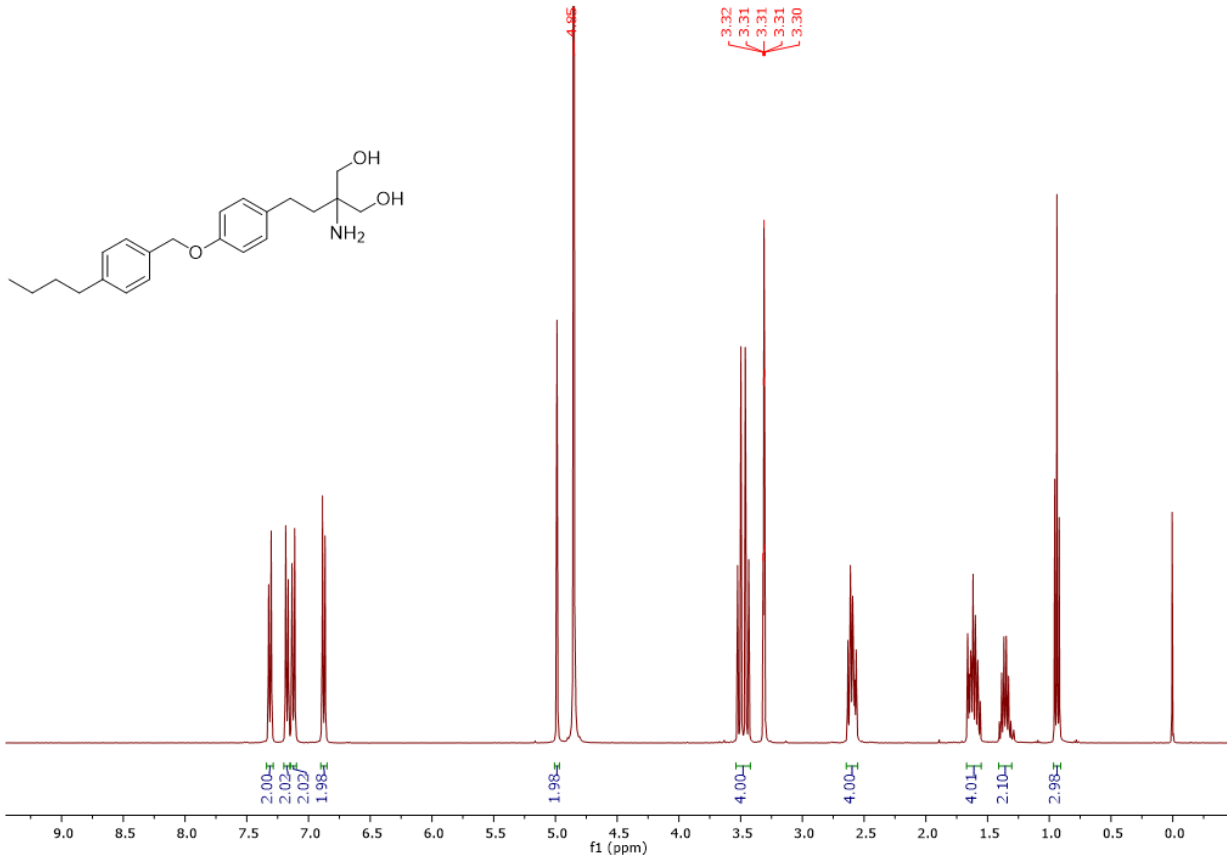

Figure S26: ^{1}H NMR spectra of compound **29** in CD_3OD.

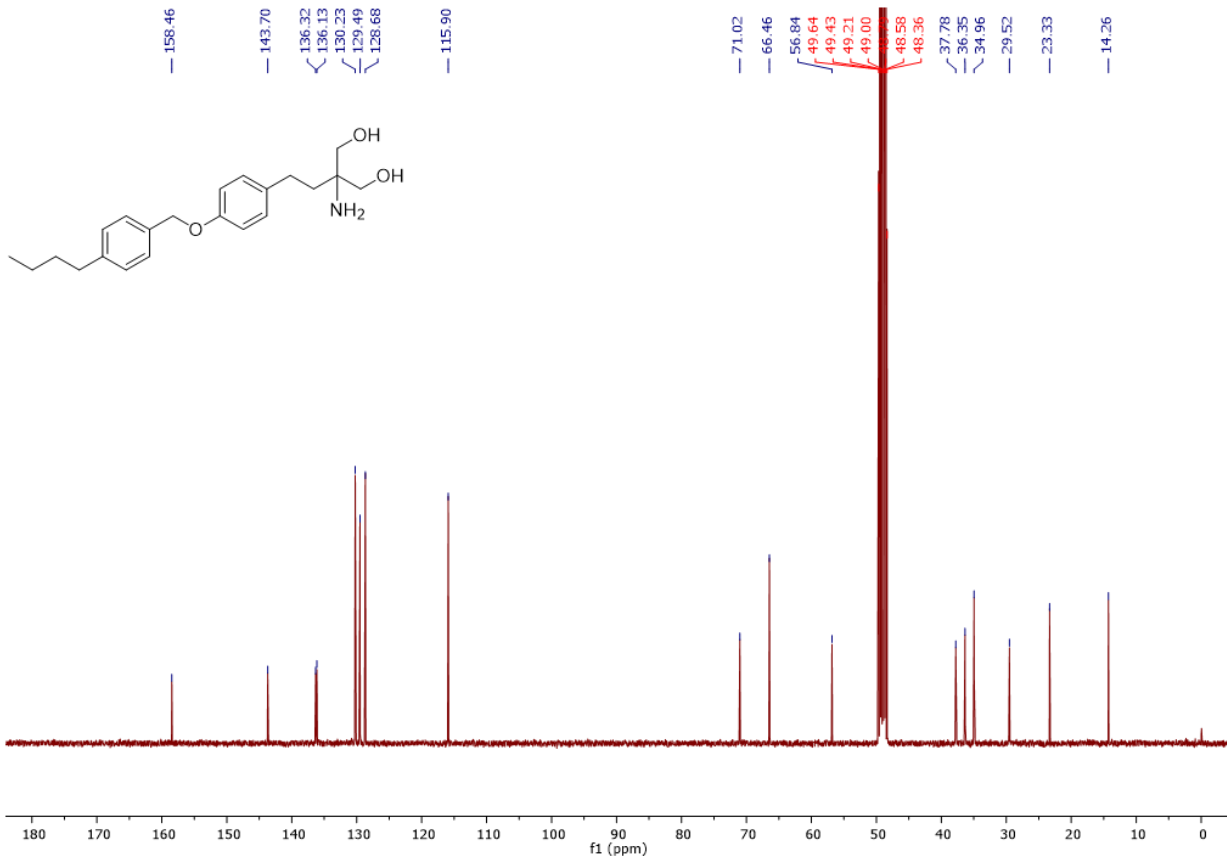

Figure S27: ^{13}C NMR spectra of compound **29** in CD_3OD.

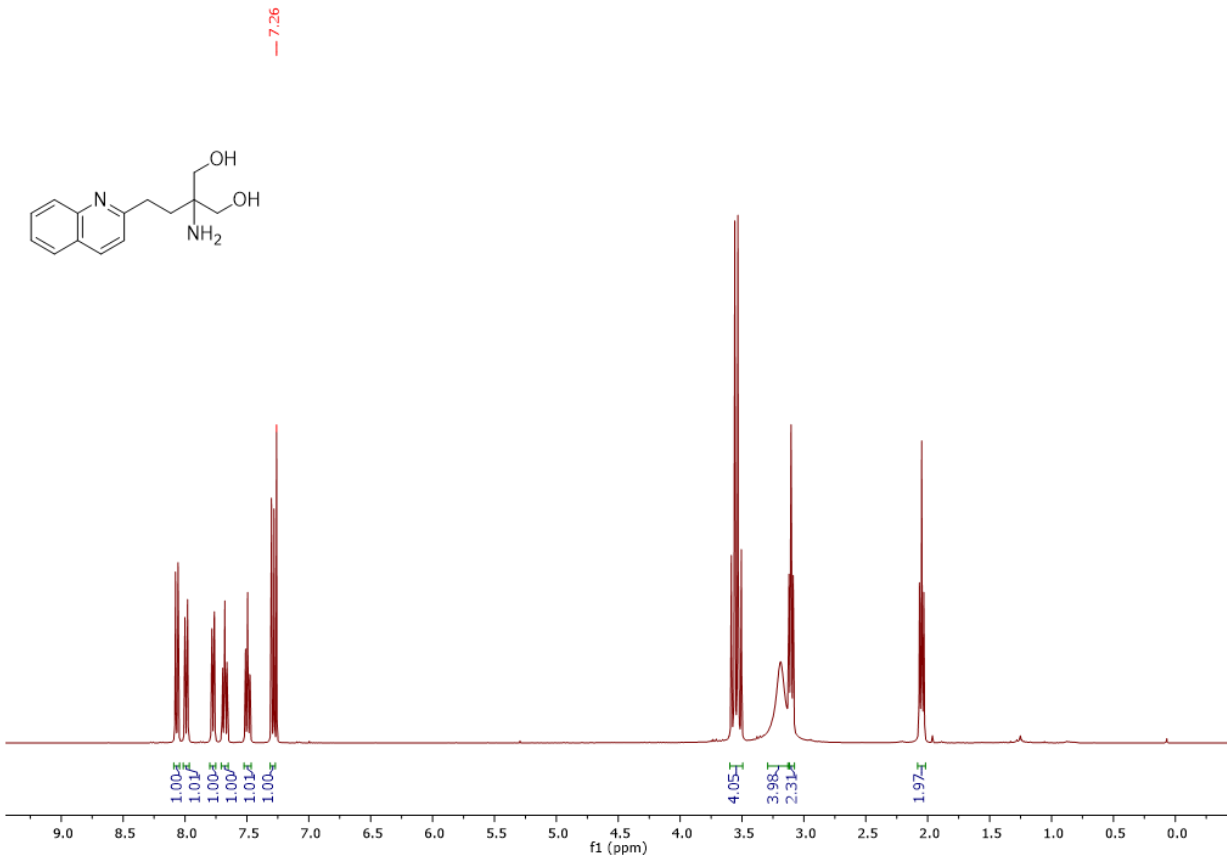

Figure S28: ^{1}H NMR spectra of compound **33** in $CDCl_3$.

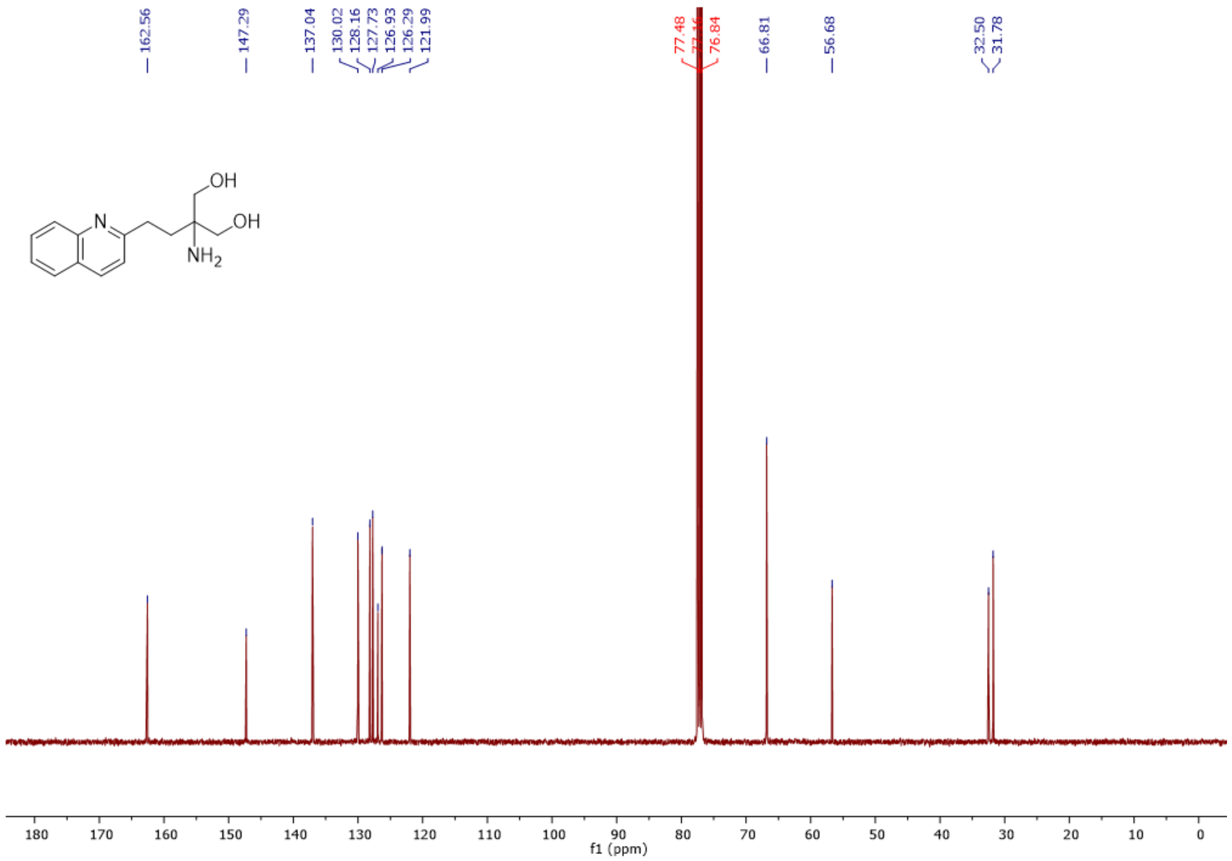

Figure S29: ^{13}C NMR spectra of compound **33** in $CDCl_3$.

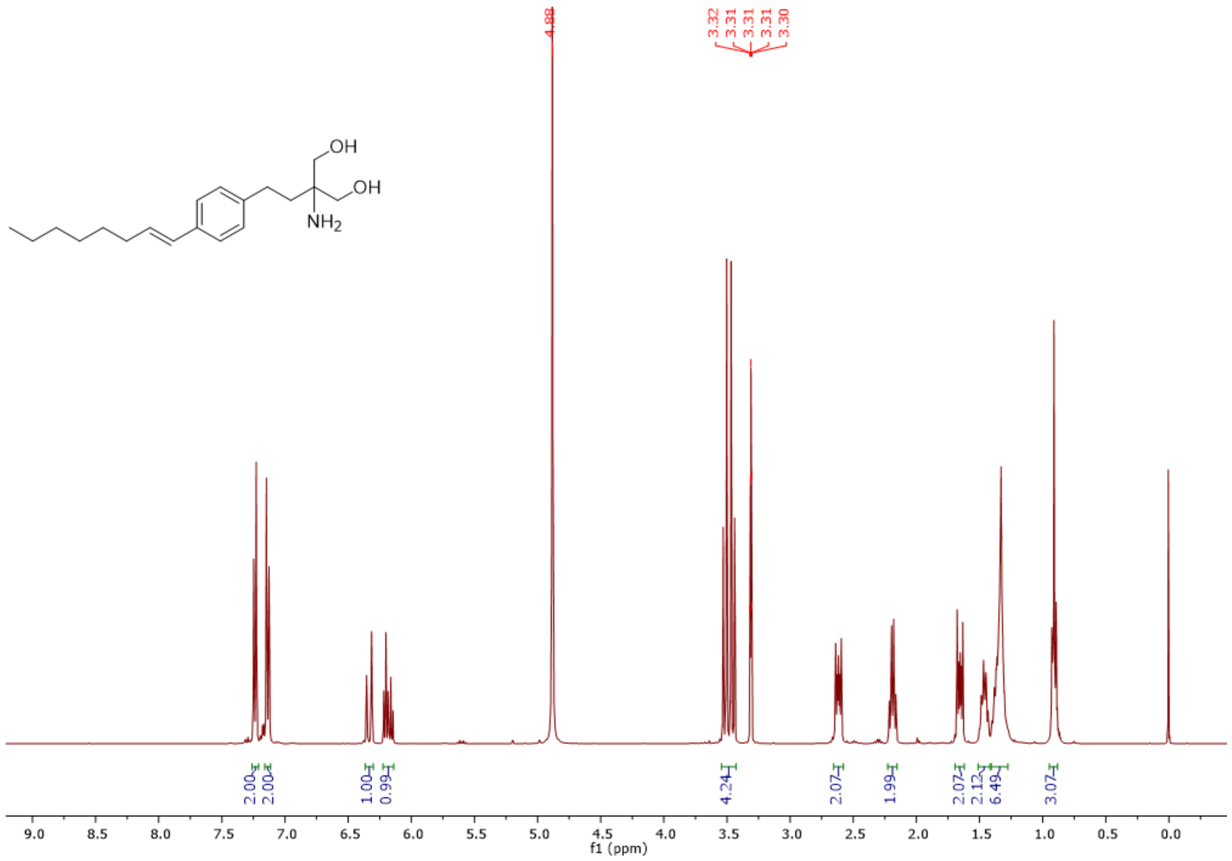

Figure S30: ^{1}H NMR spectra of compound **42** in CD_3OD.

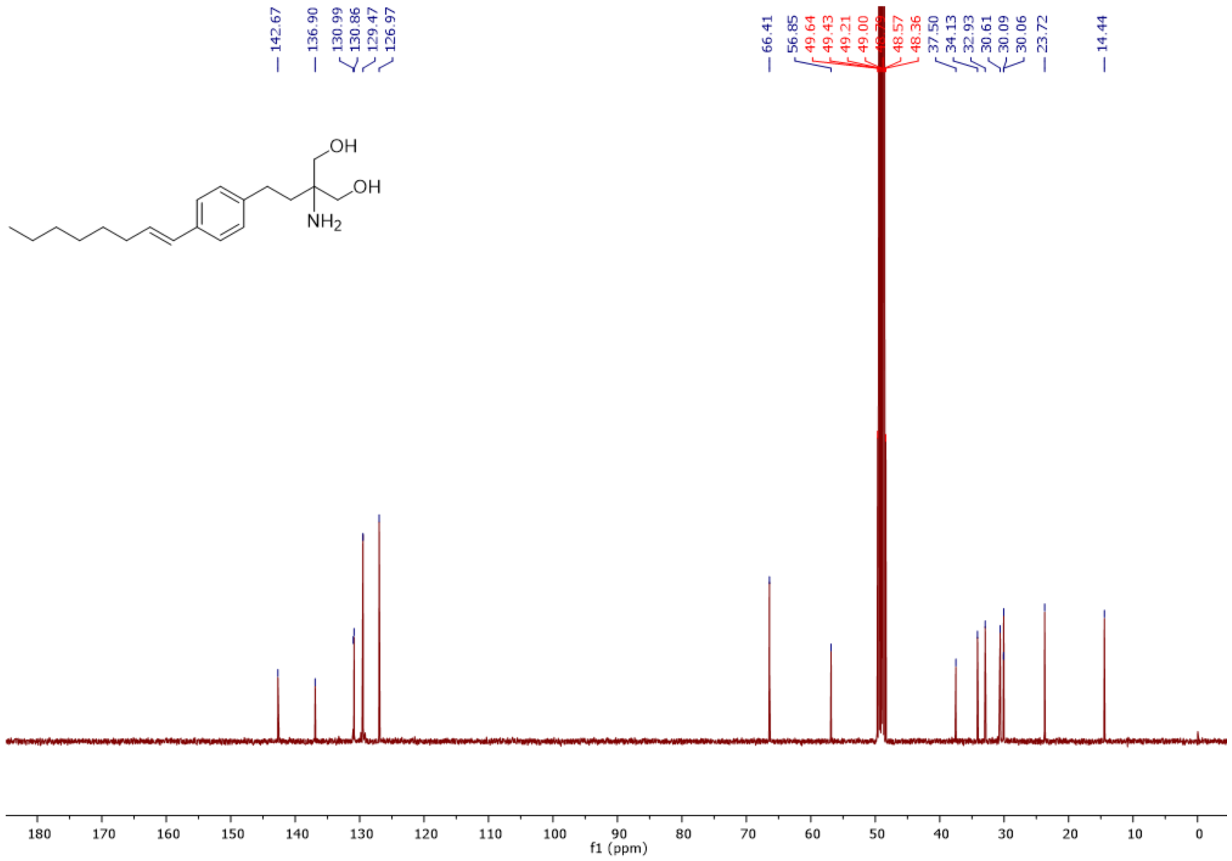

Figure S31: ^{13}C NMR spectra of compound **42** in CD_3OD.

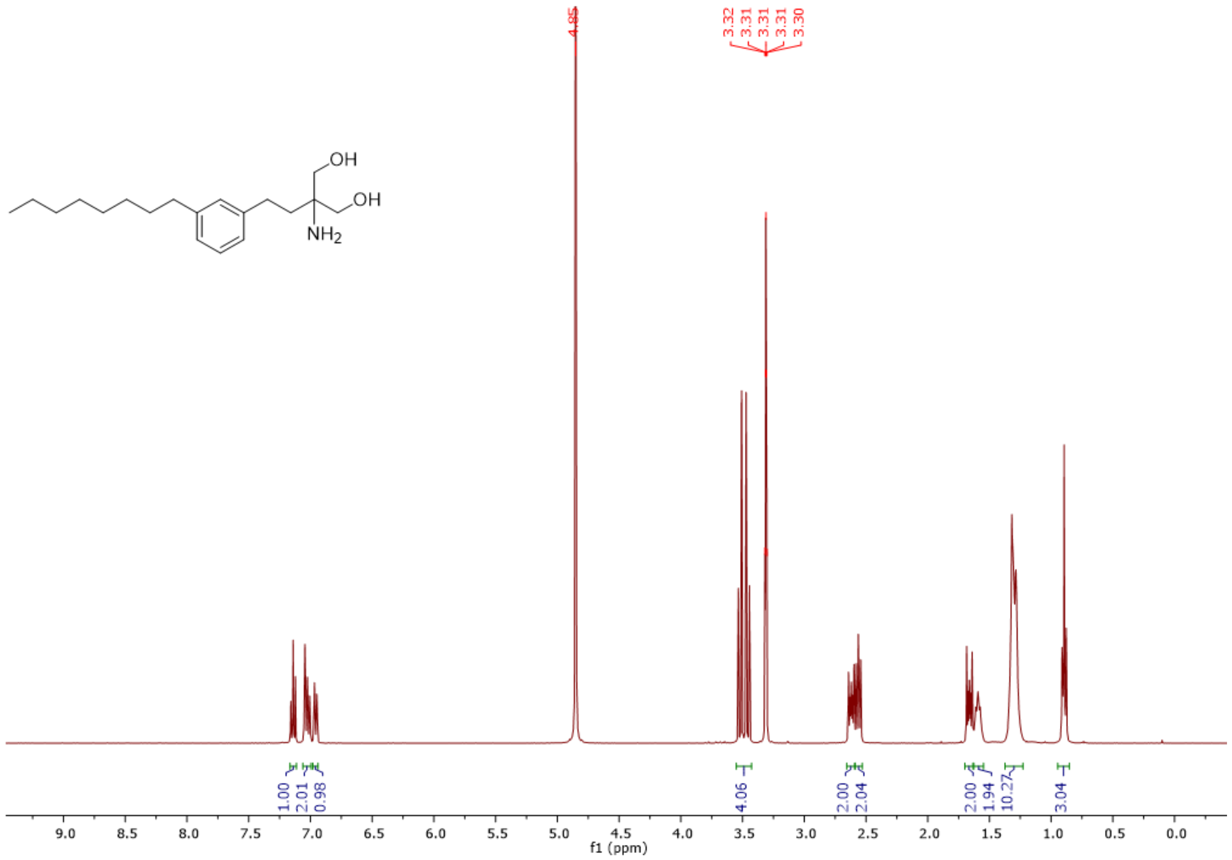

Figure S32: ^{1}H NMR spectra of compound **43** in CD_3OD.

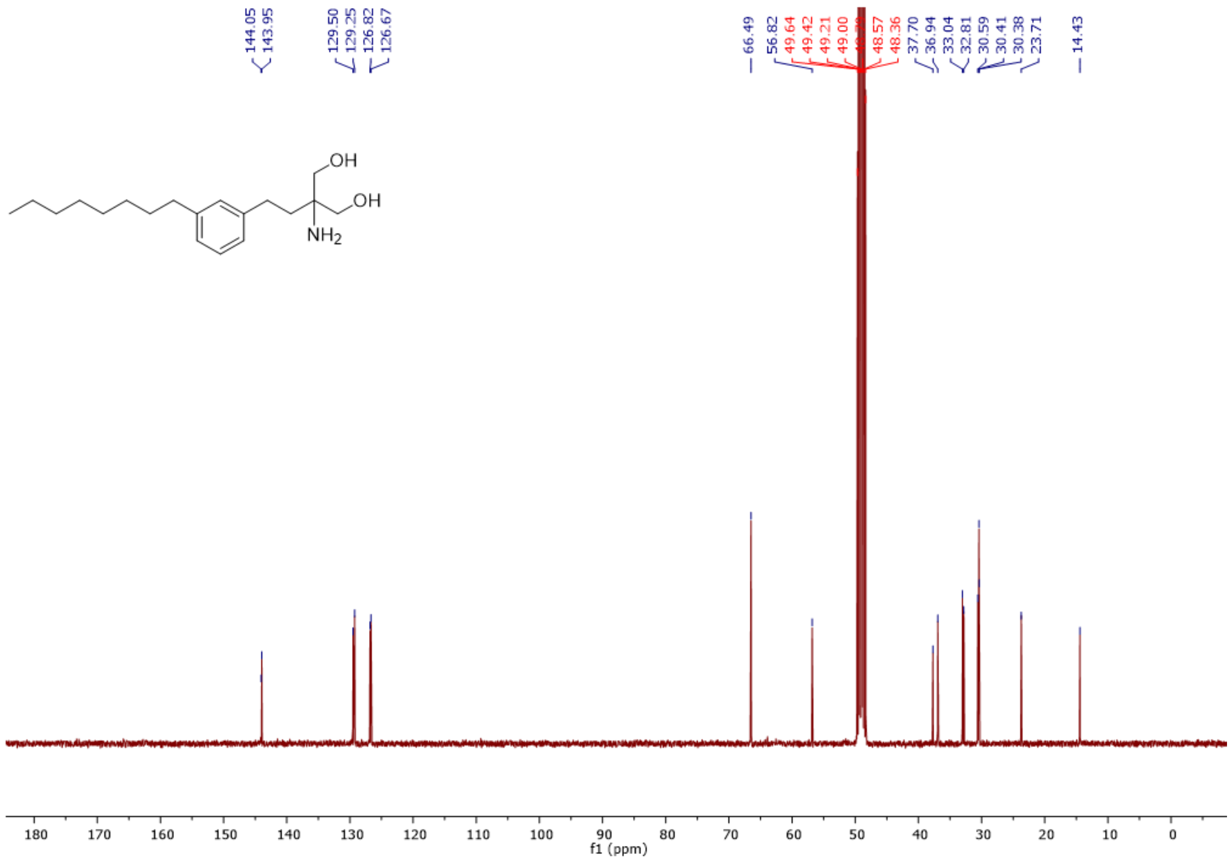

Figure S33: ^{13}C NMR spectra of compound **43** in CD_3OD.

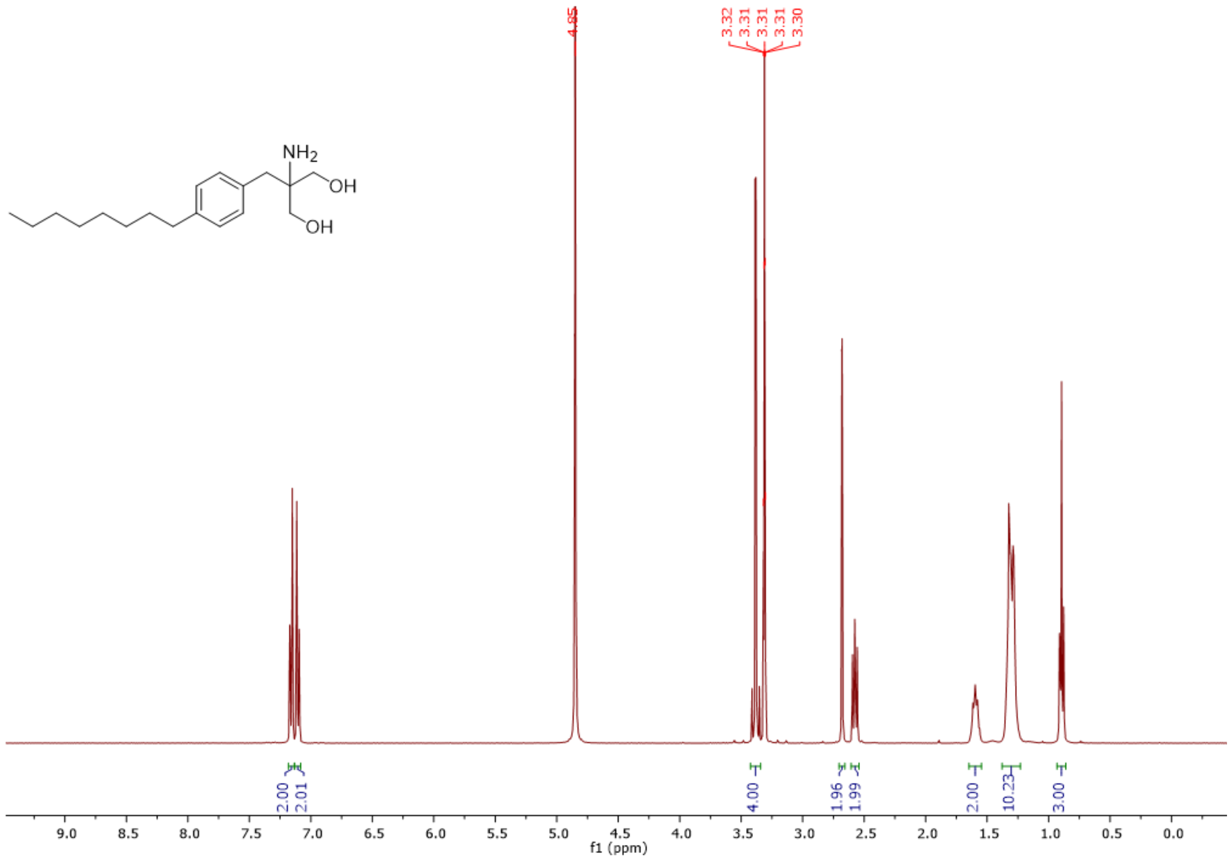

Figure S34: ^{1}H NMR spectra of compound **44** in CD_3OD.

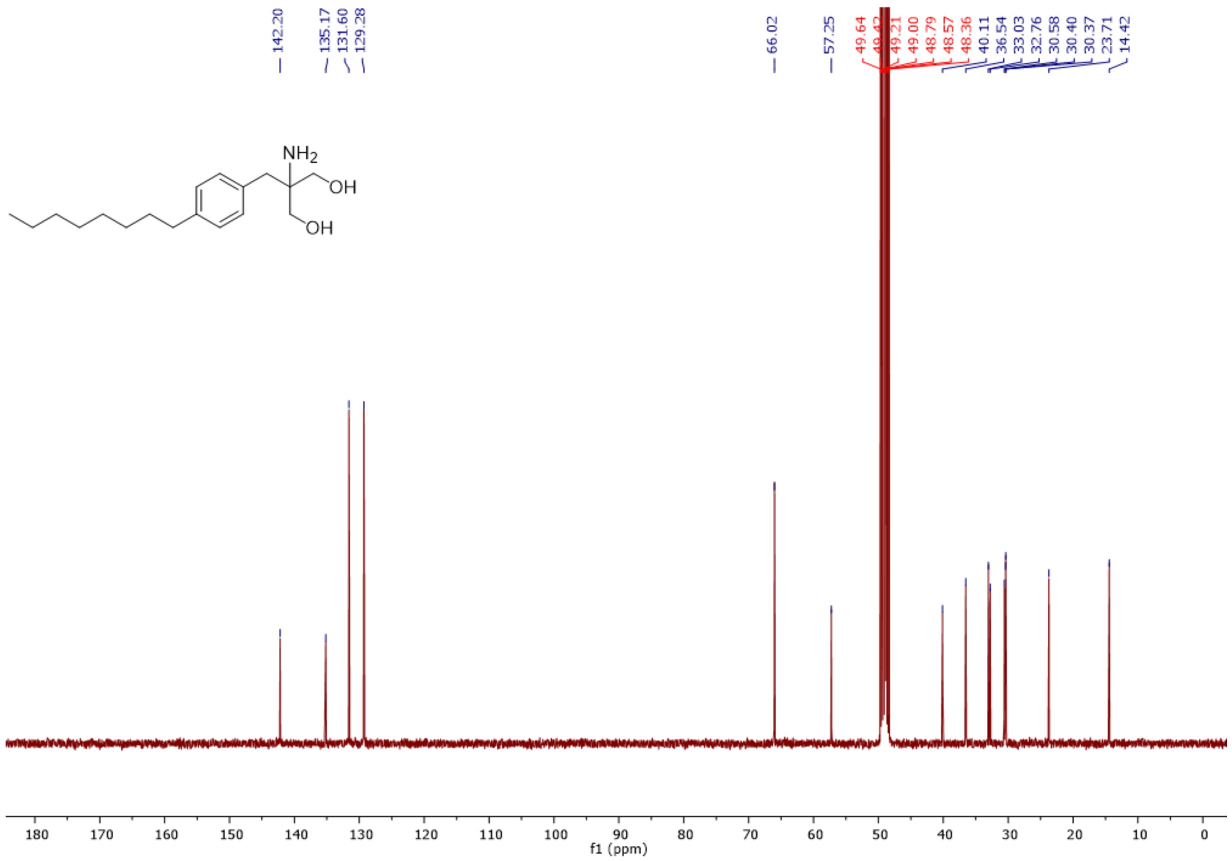

Figure S35: ^{13}C NMR spectra of compound **44** in CD_3OD.

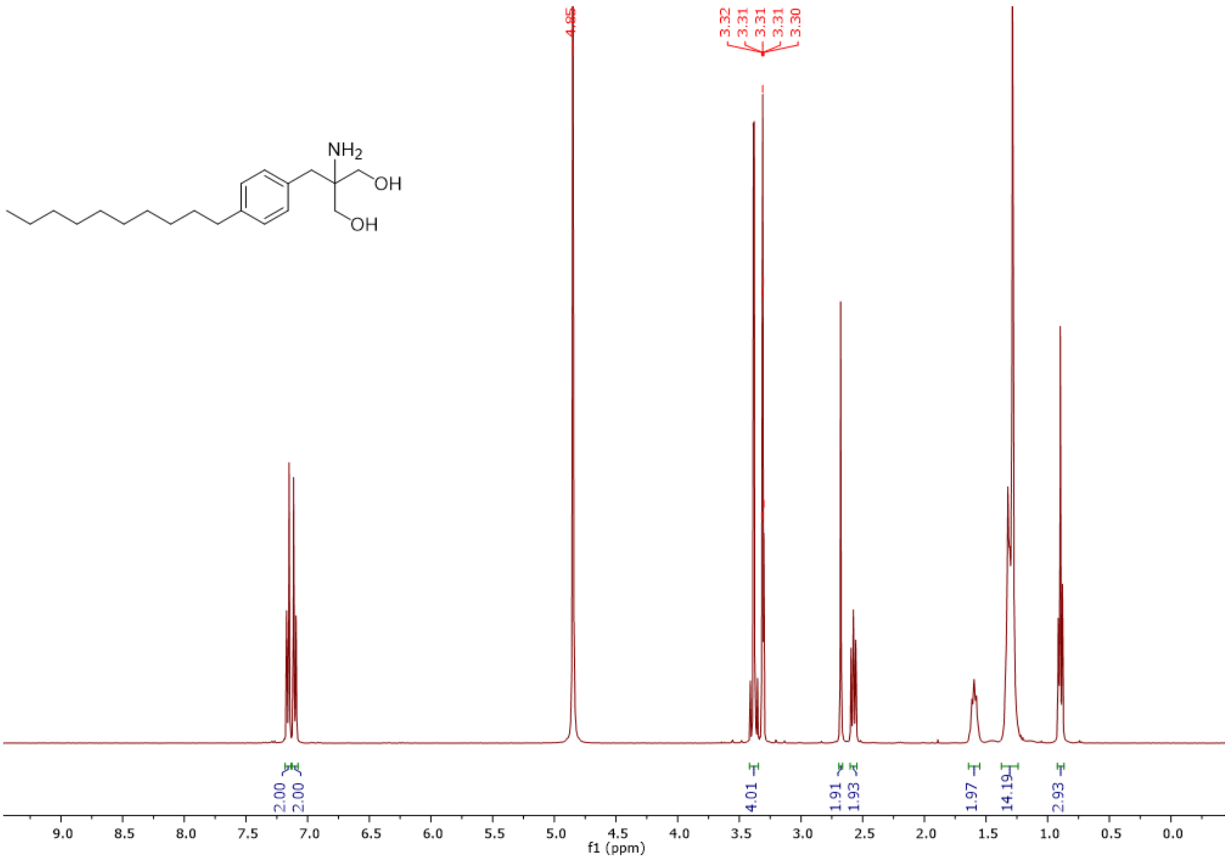

Figure S36: ^{1}H NMR spectra of compound **45** in CD_3OD.

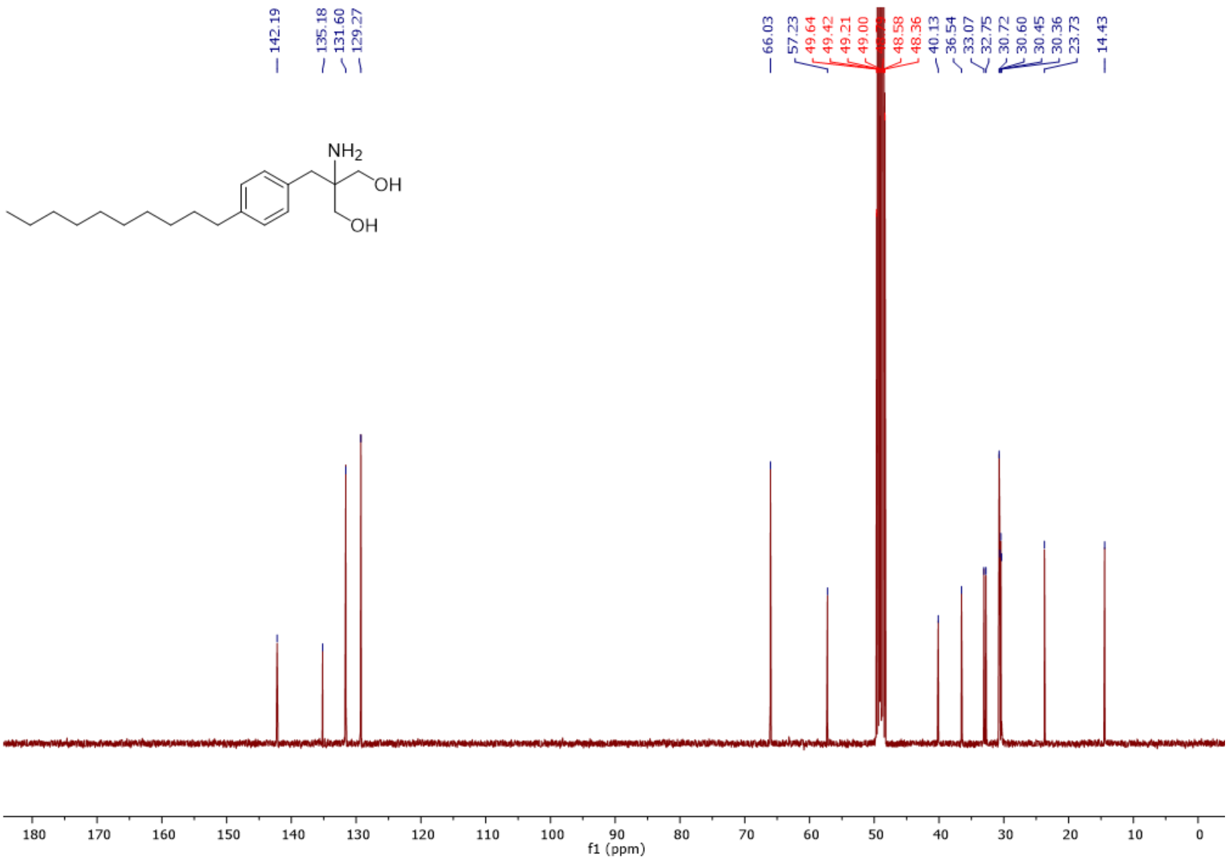

Figure S37: ^{13}C NMR spectra of compound **45** in CD_3OD.

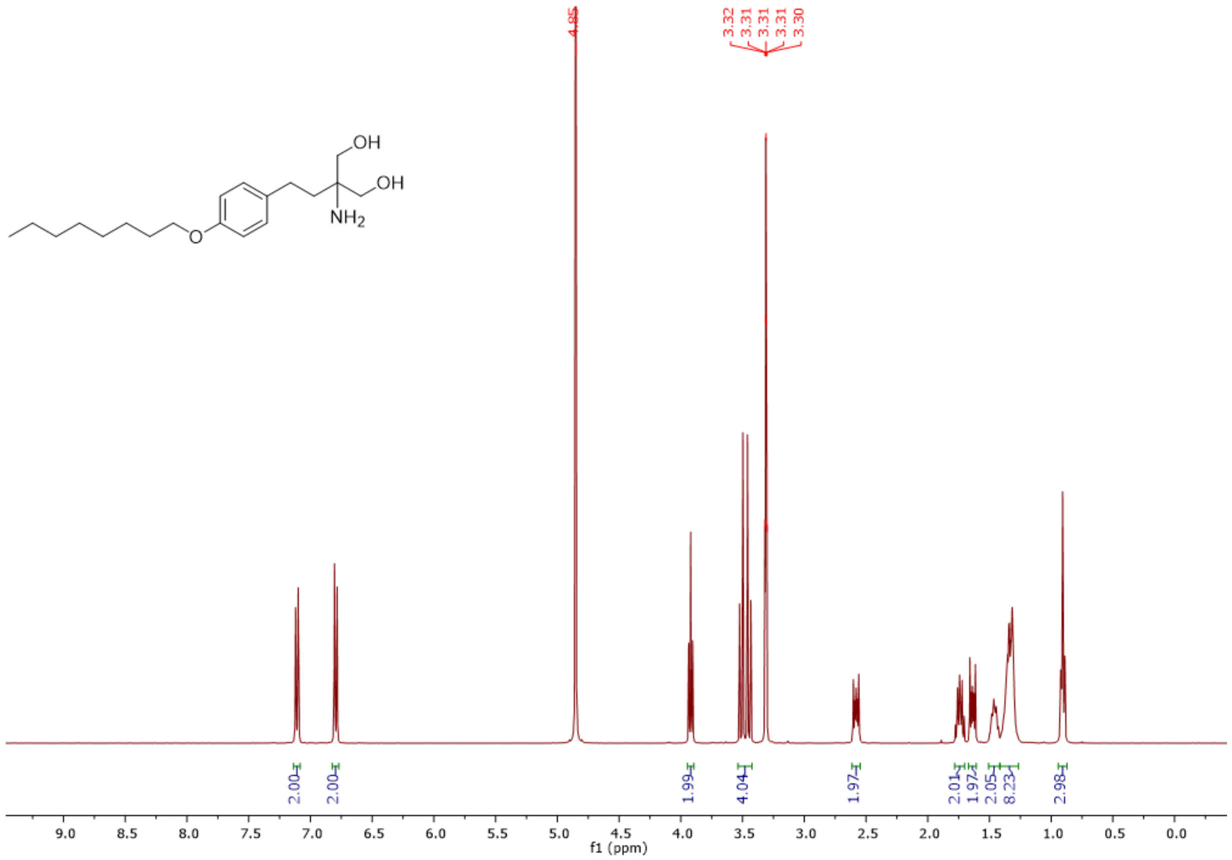

Figure S38: ^{1}H NMR spectra of compound **48** in CD_3OD.

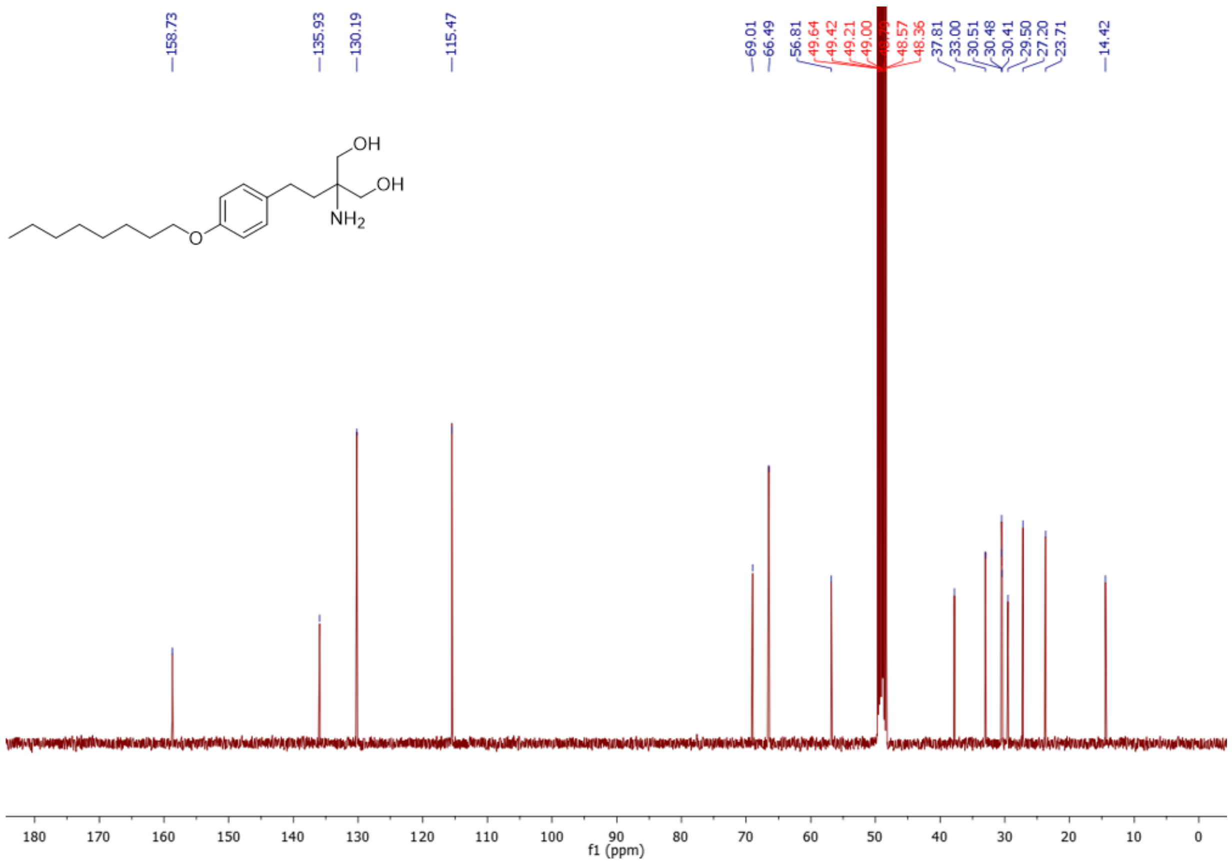

Figure S39: ^{13}C NMR spectra of compound **48** in CD_3OD.

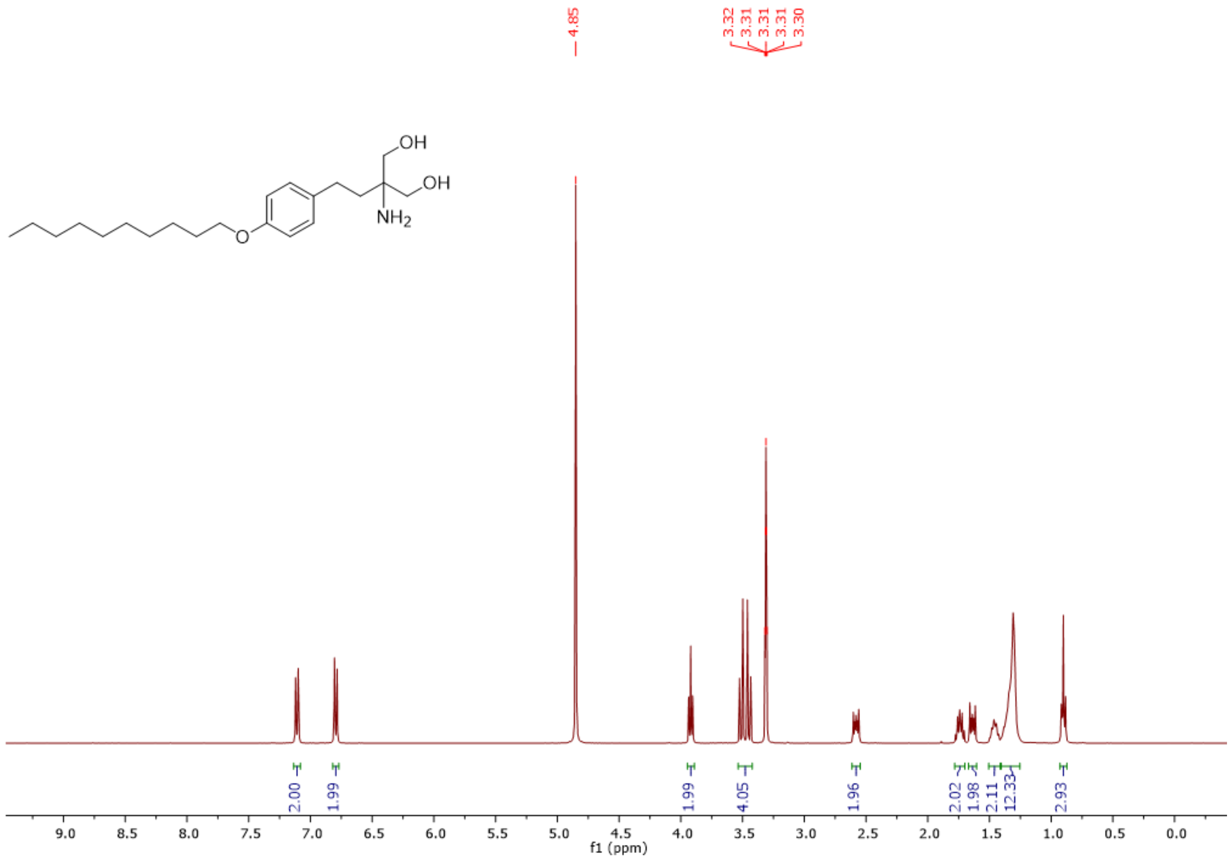

Figure S40: ^{1}H NMR spectra of compound **49** in CD_3OD.

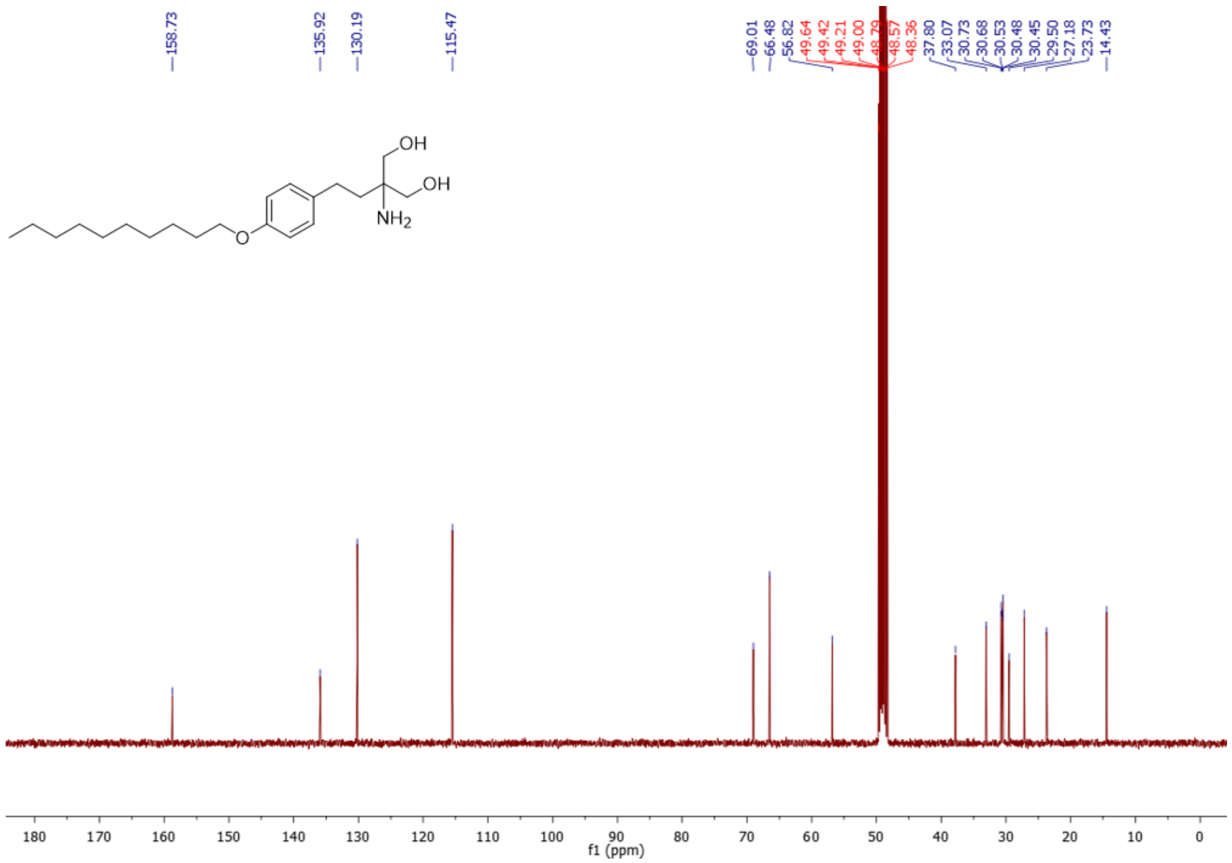

Figure S41: ^{13}C NMR spectra of compound **49** in CD_3OD.

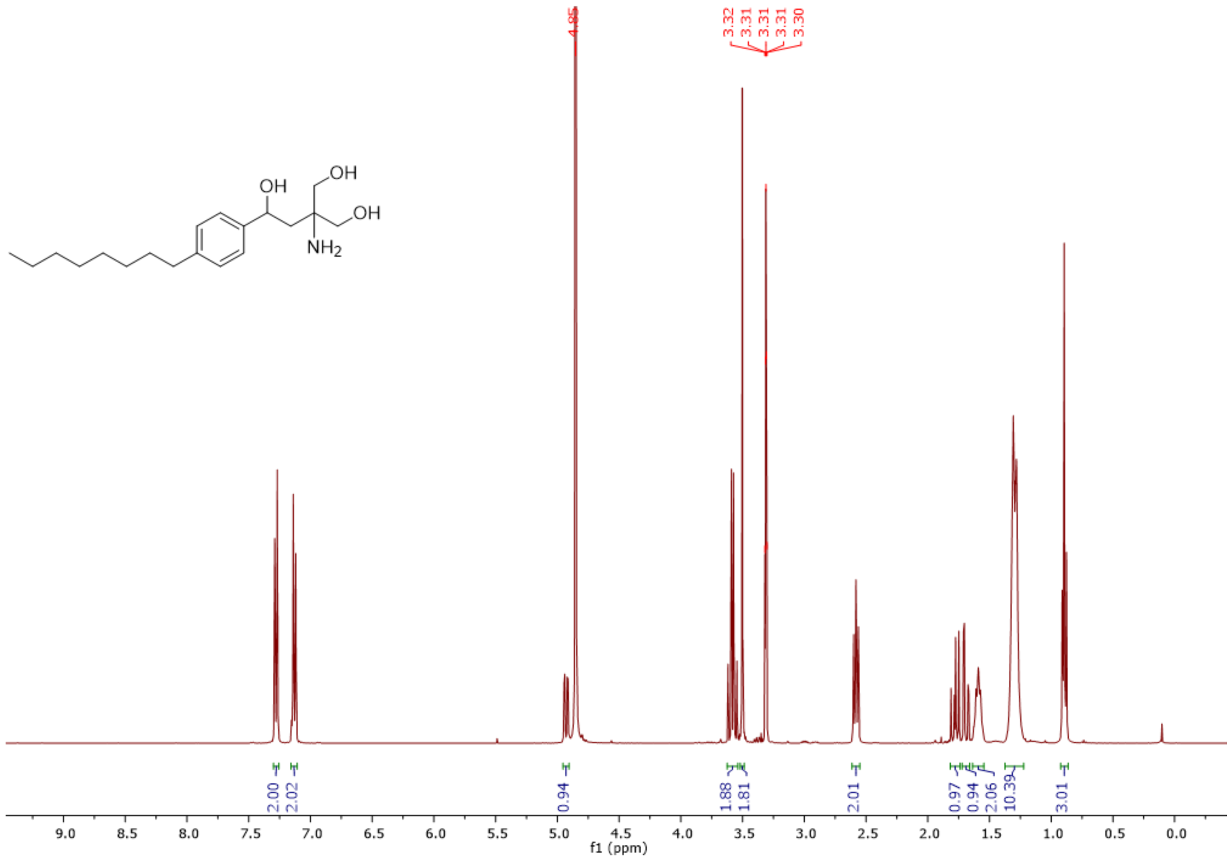

Figure S42: ^{1}H NMR spectra of compound **54** in CD_3OD.

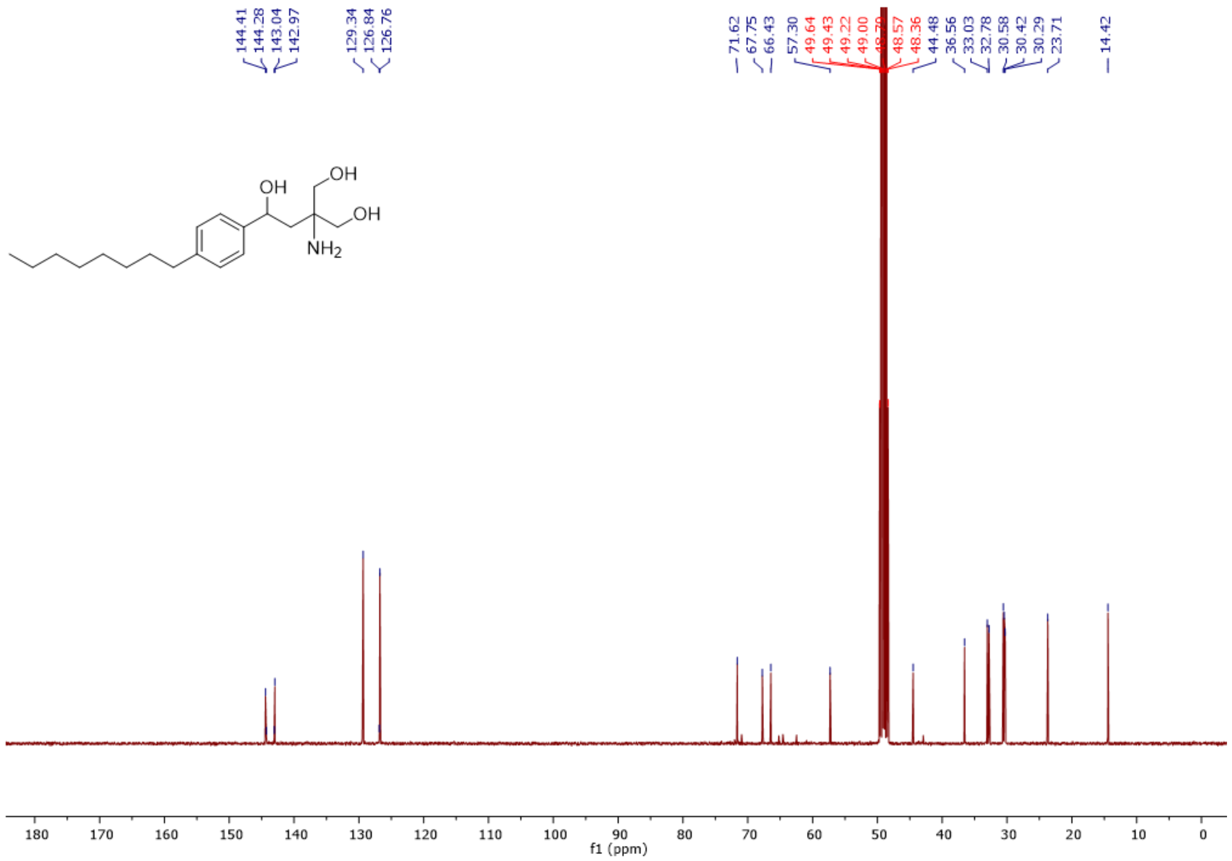

Figure S43: ^{13}C NMR spectra of compound **54** in CD_3OD.

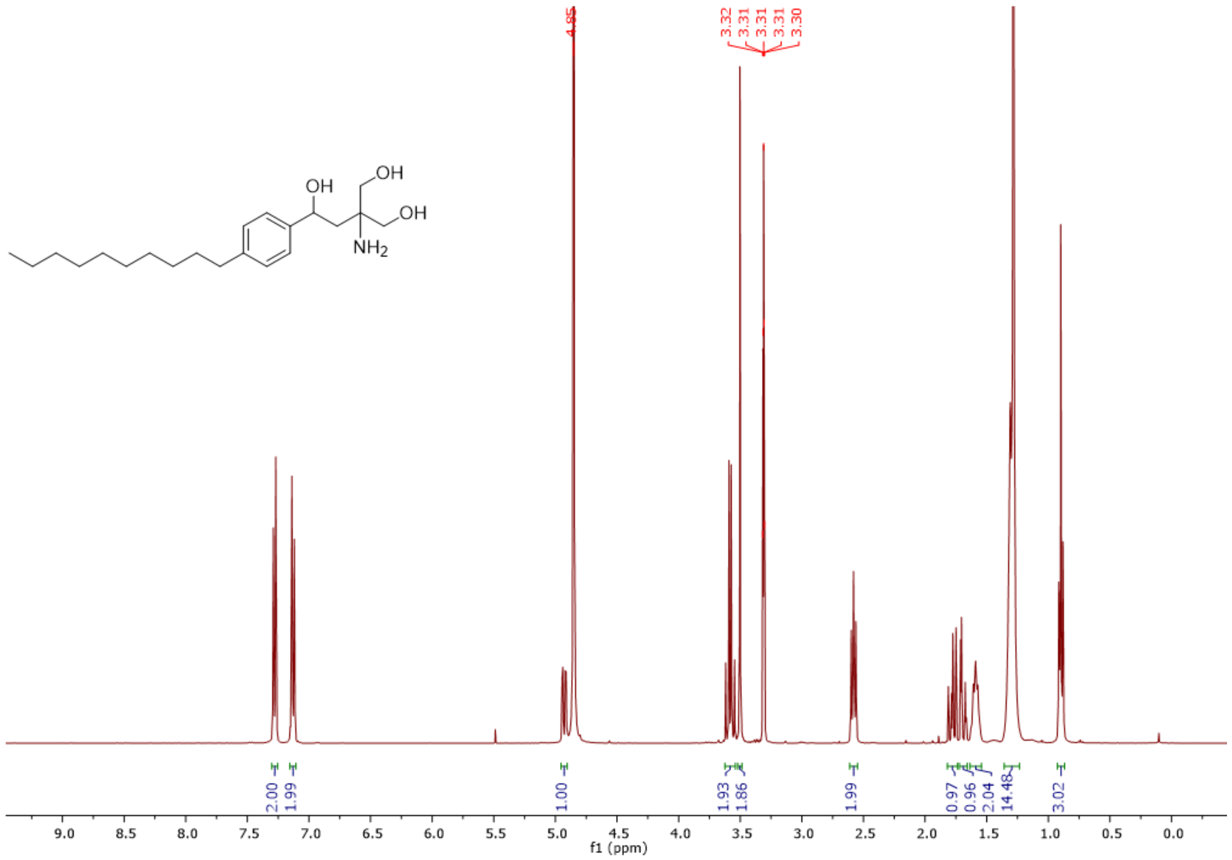

Figure S44: ^{1}H NMR spectra of compound **55** in CD_3OD.

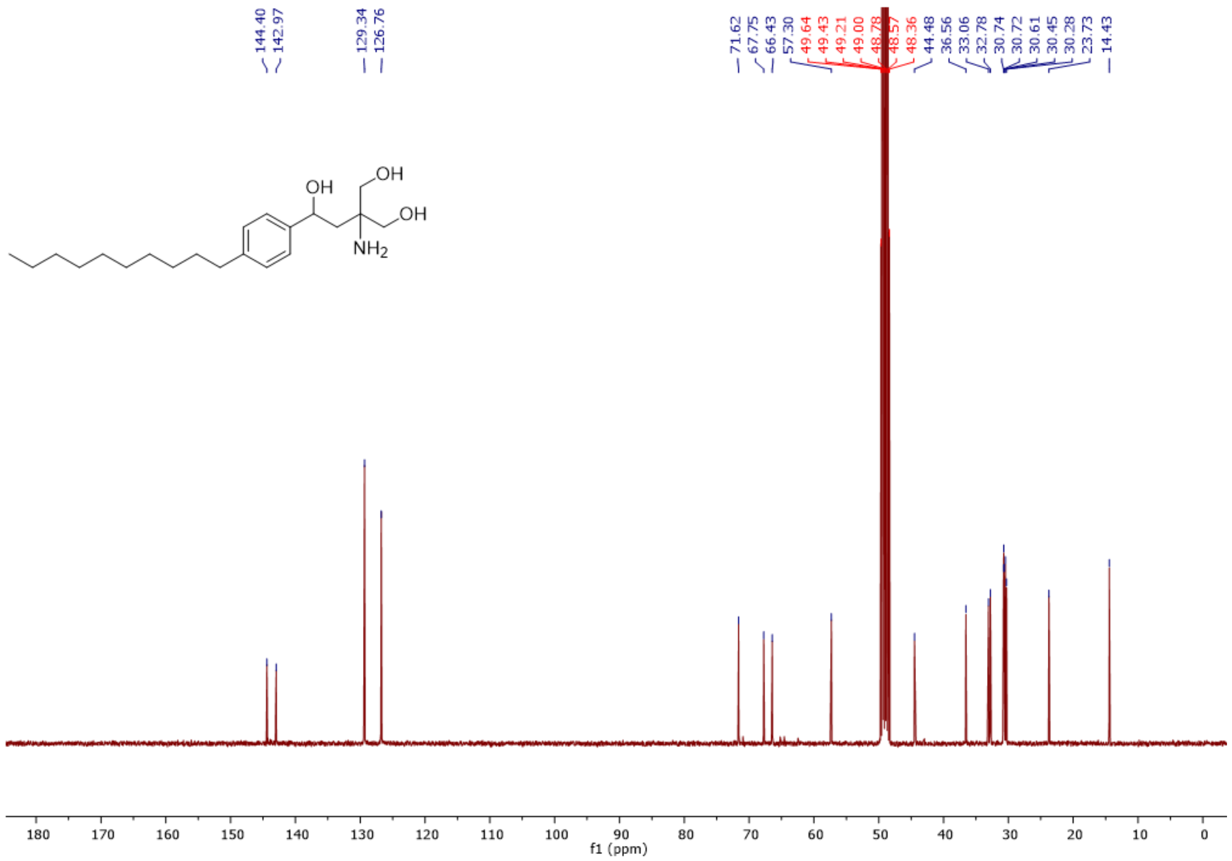

Figure S45: ^{13}C NMR spectra of compound **55** in CD_3OD.

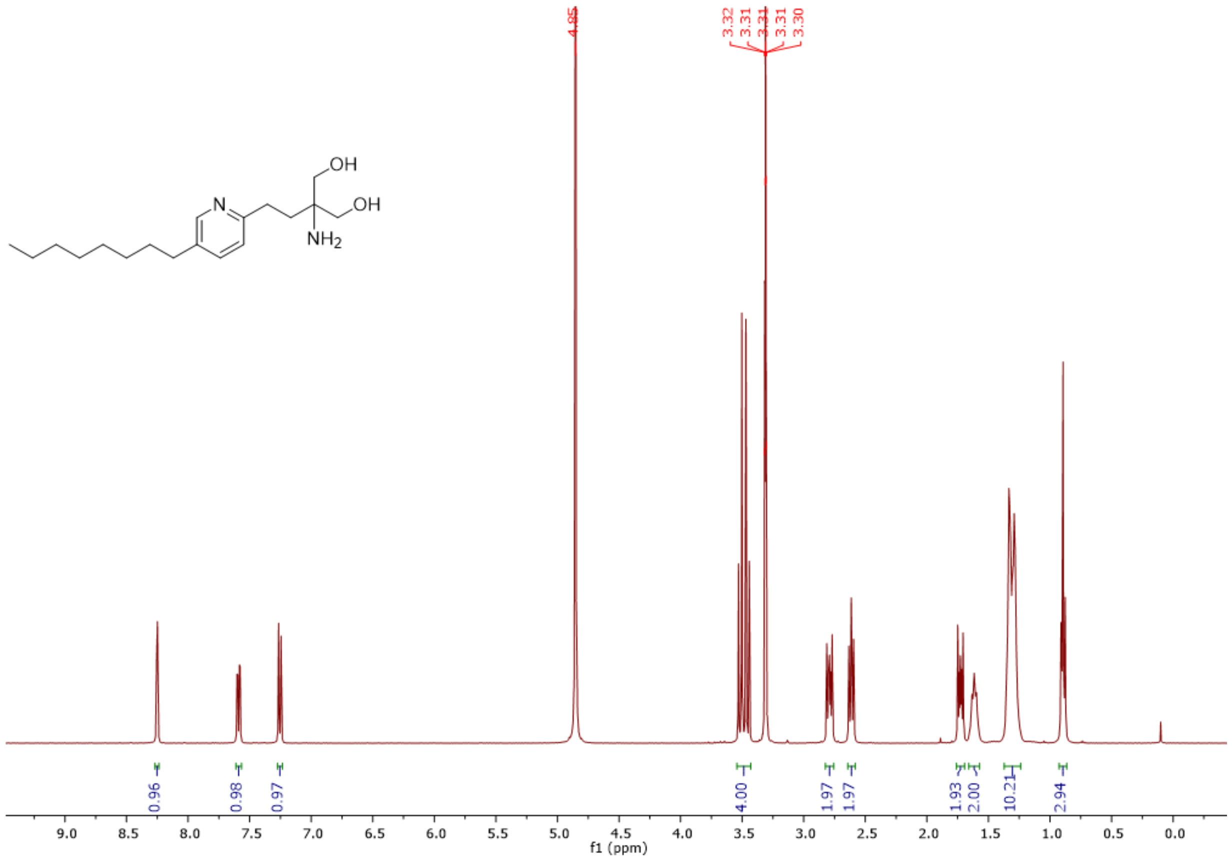

Figure S46: ^{1}H NMR spectra of compound **61** in CD_3OD.

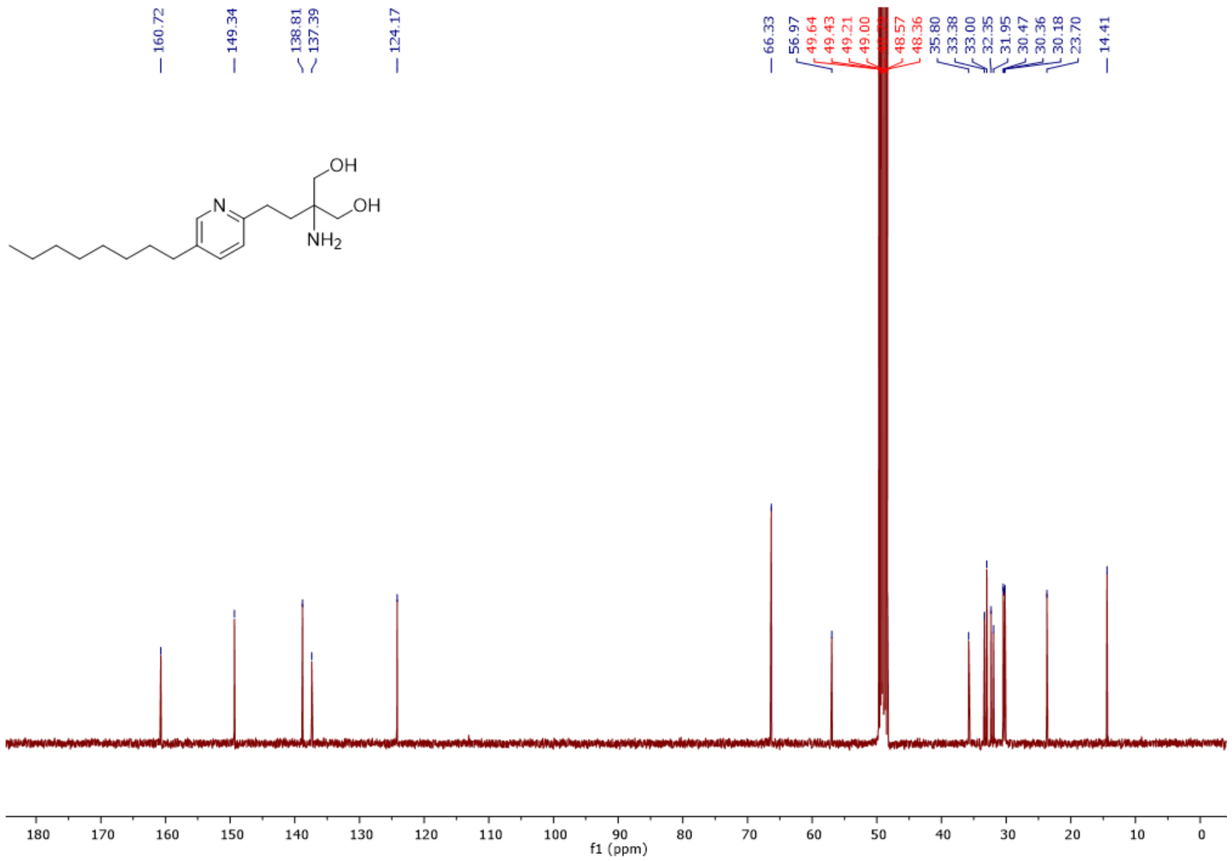

Figure S47: ^{13}C NMR spectra of compound **61** in CD_3OD.

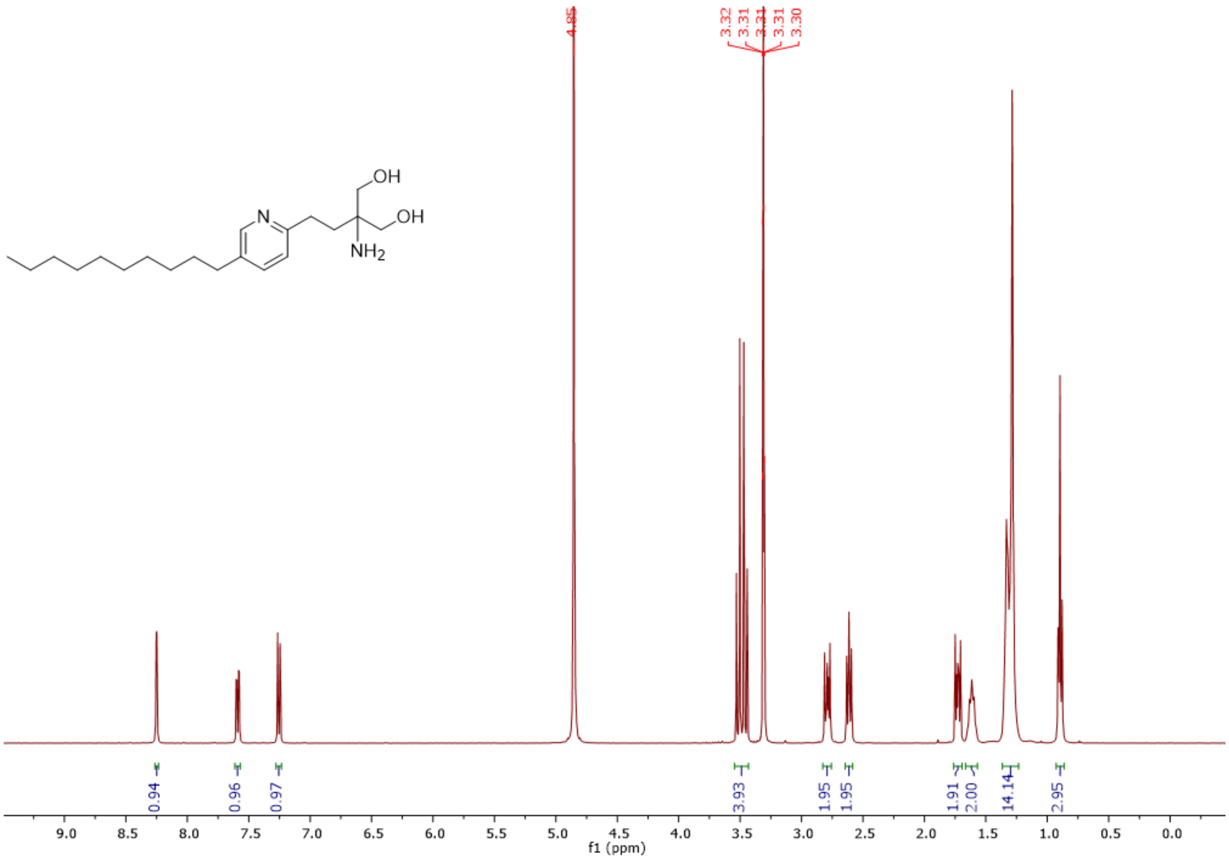

Figure S48: ^{1}H NMR spectra of compound **62** in CD_3OD.

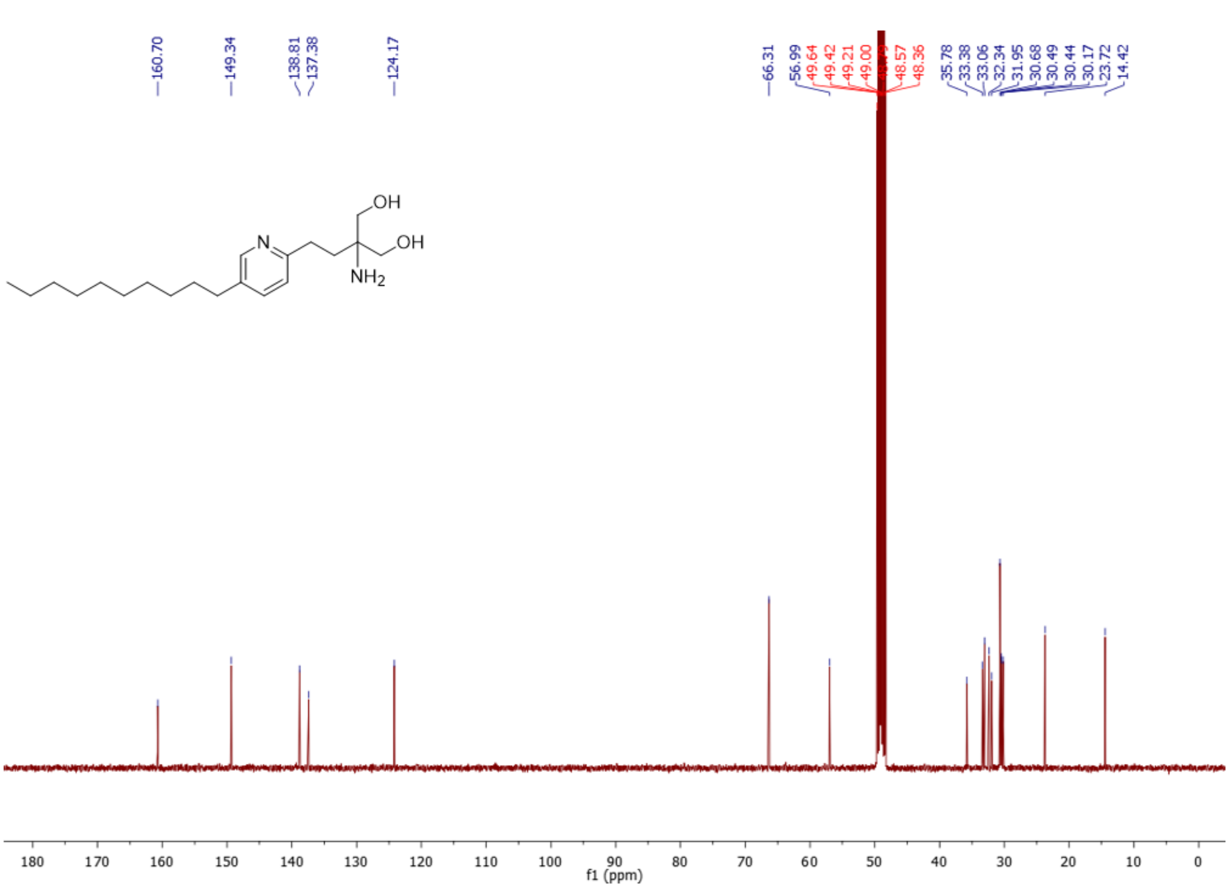

Figure S49: ^{13}C NMR spectra of compound **62** in CD_3OD.

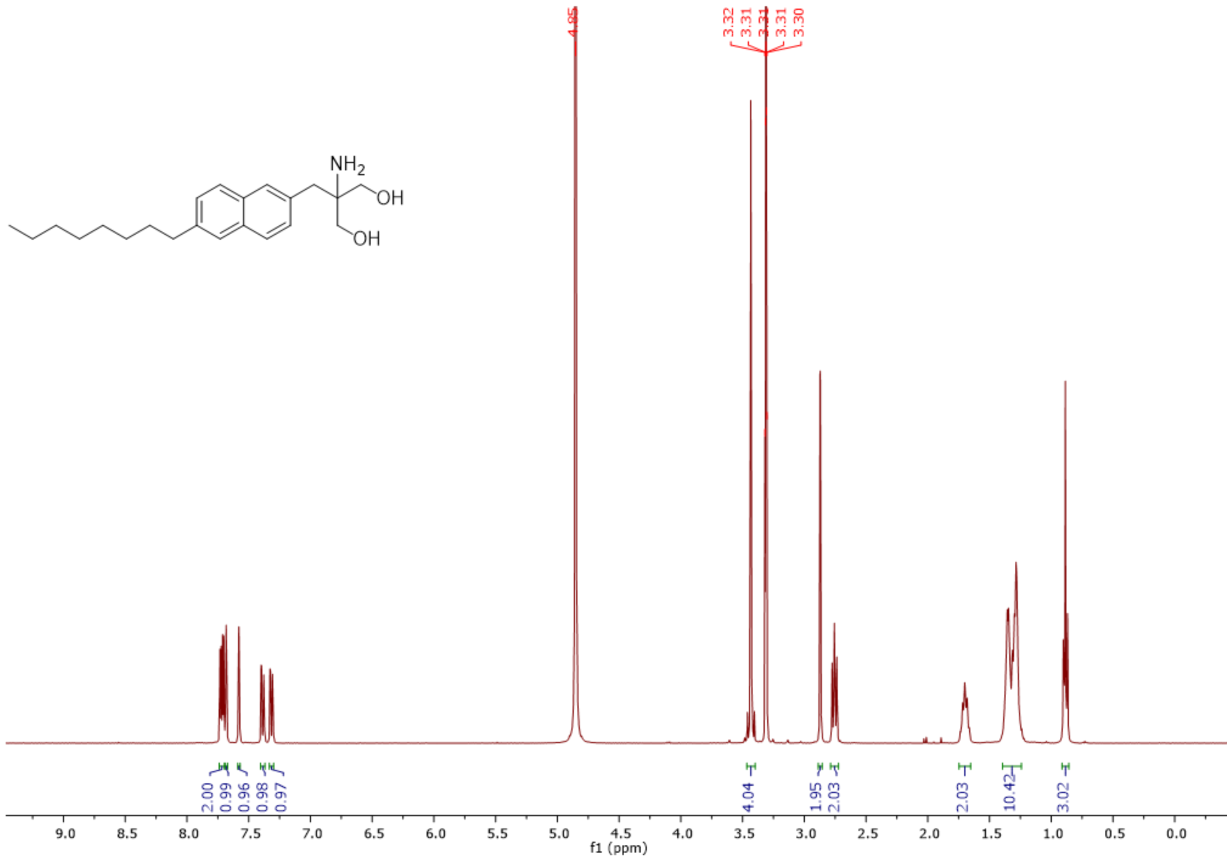

Figure S50: ^{1}H NMR spectra of compound **79** in CD_3OD.

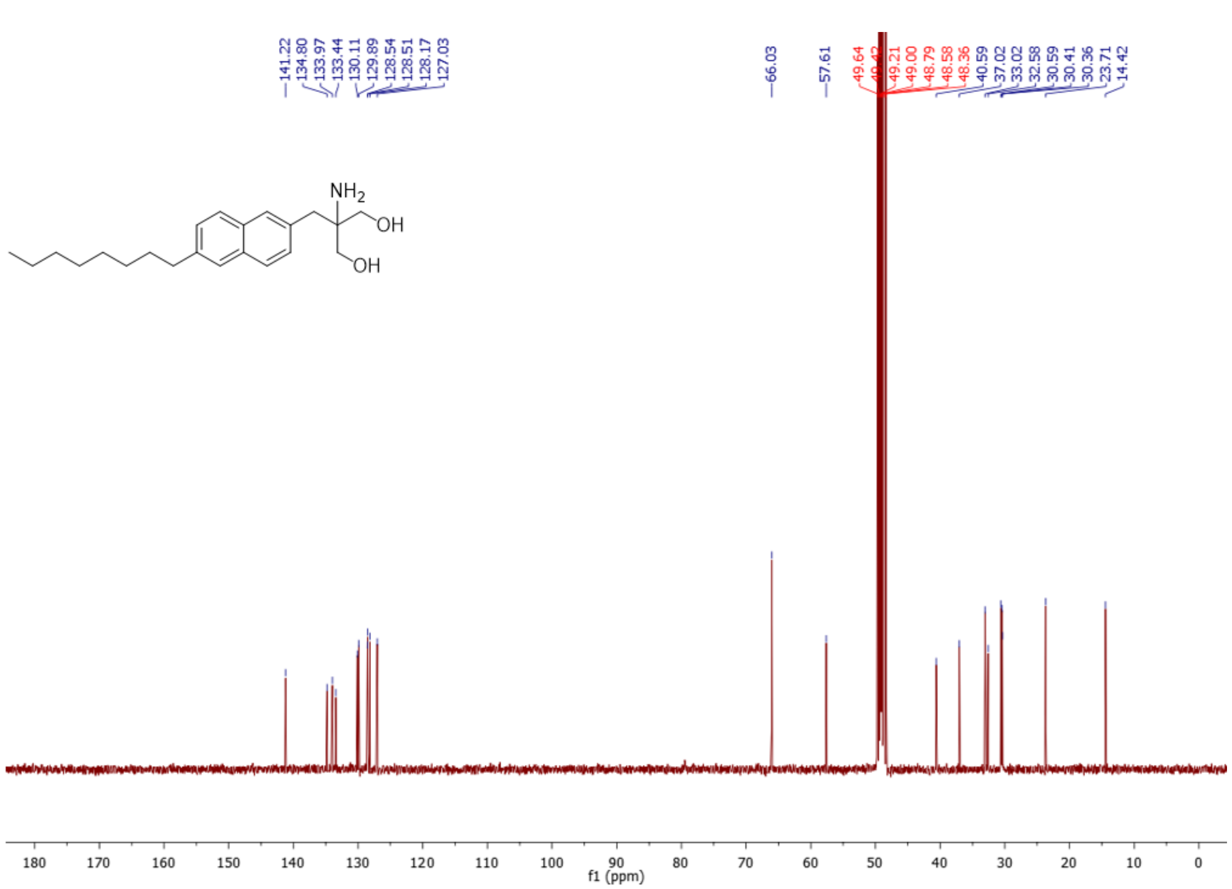

Figure S51: ^{13}C NMR spectra of compound **79** in CD_3OD.

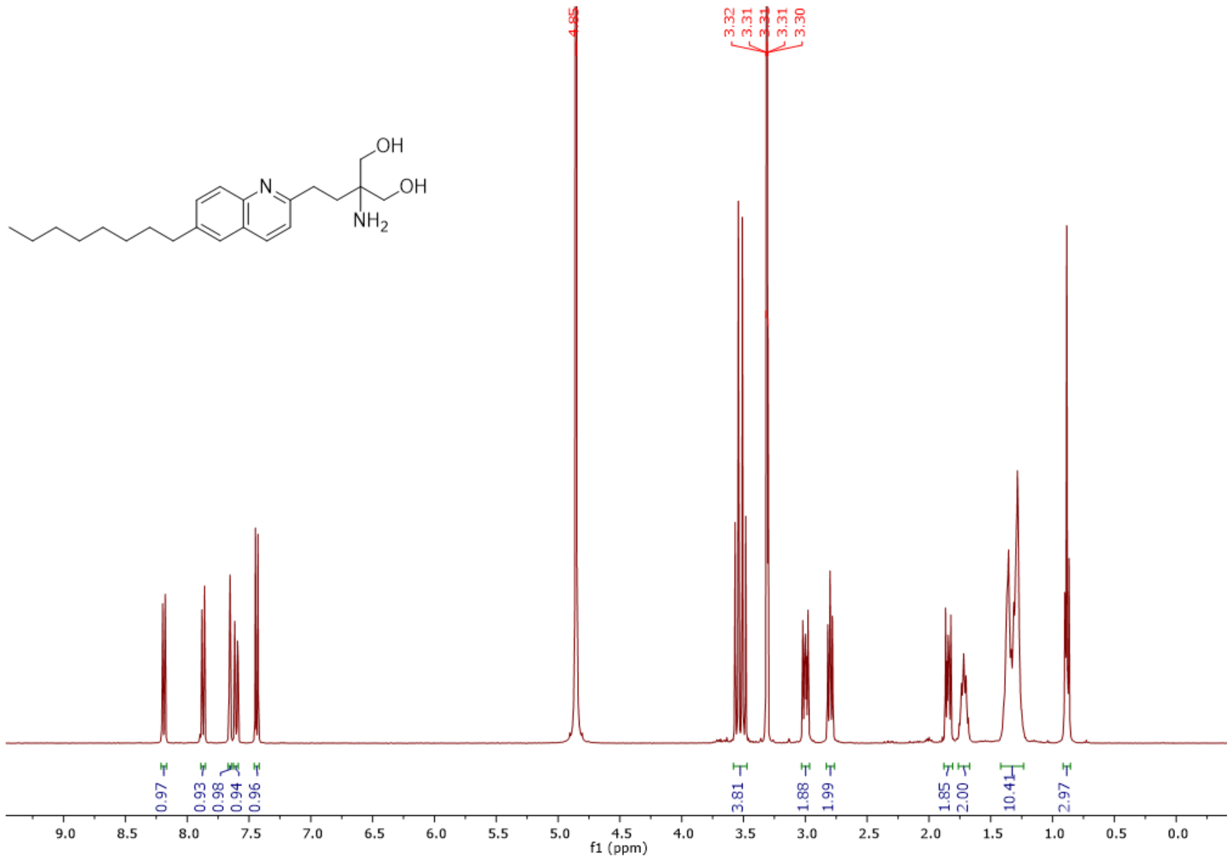

Figure S52: ^{1}H NMR spectra of compound **80** in CD_3OD.

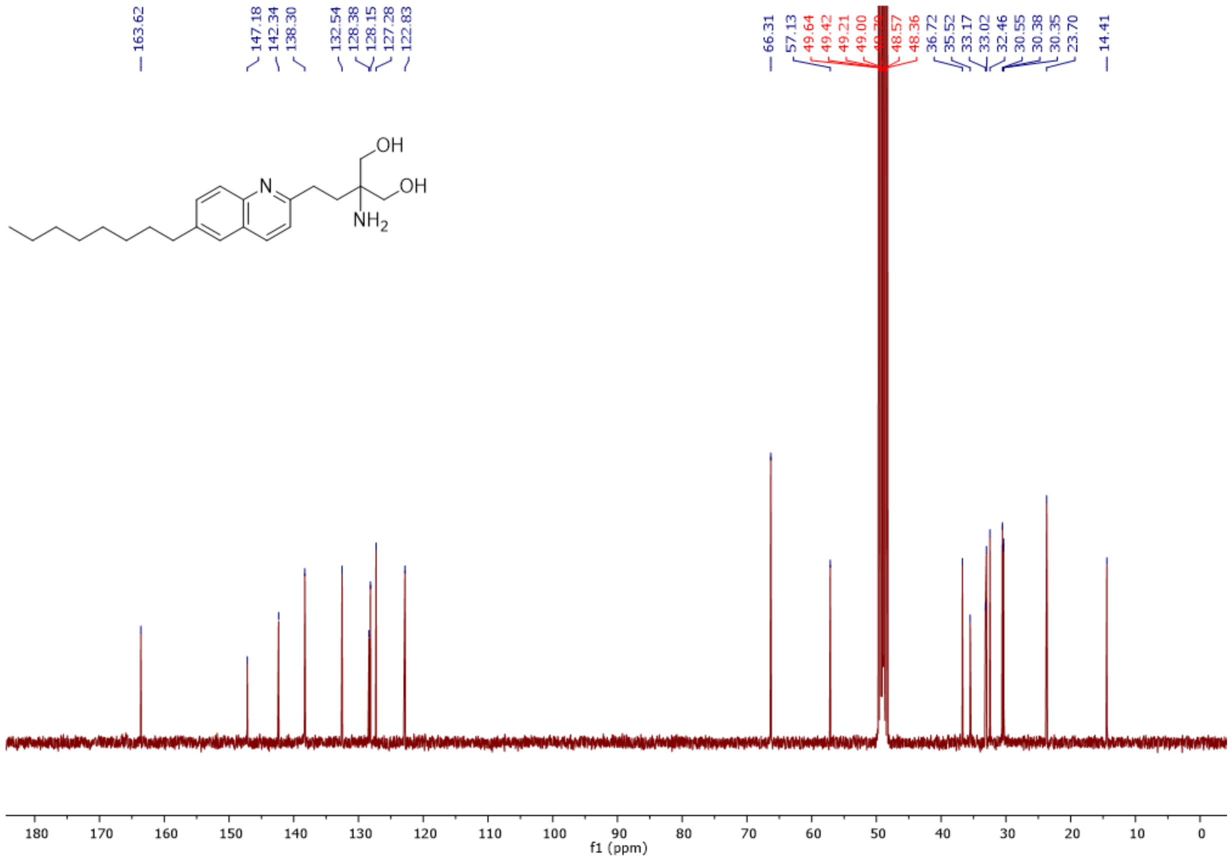

Figure S53: ^{13}C NMR spectra of compound **80** in CD_3OD.

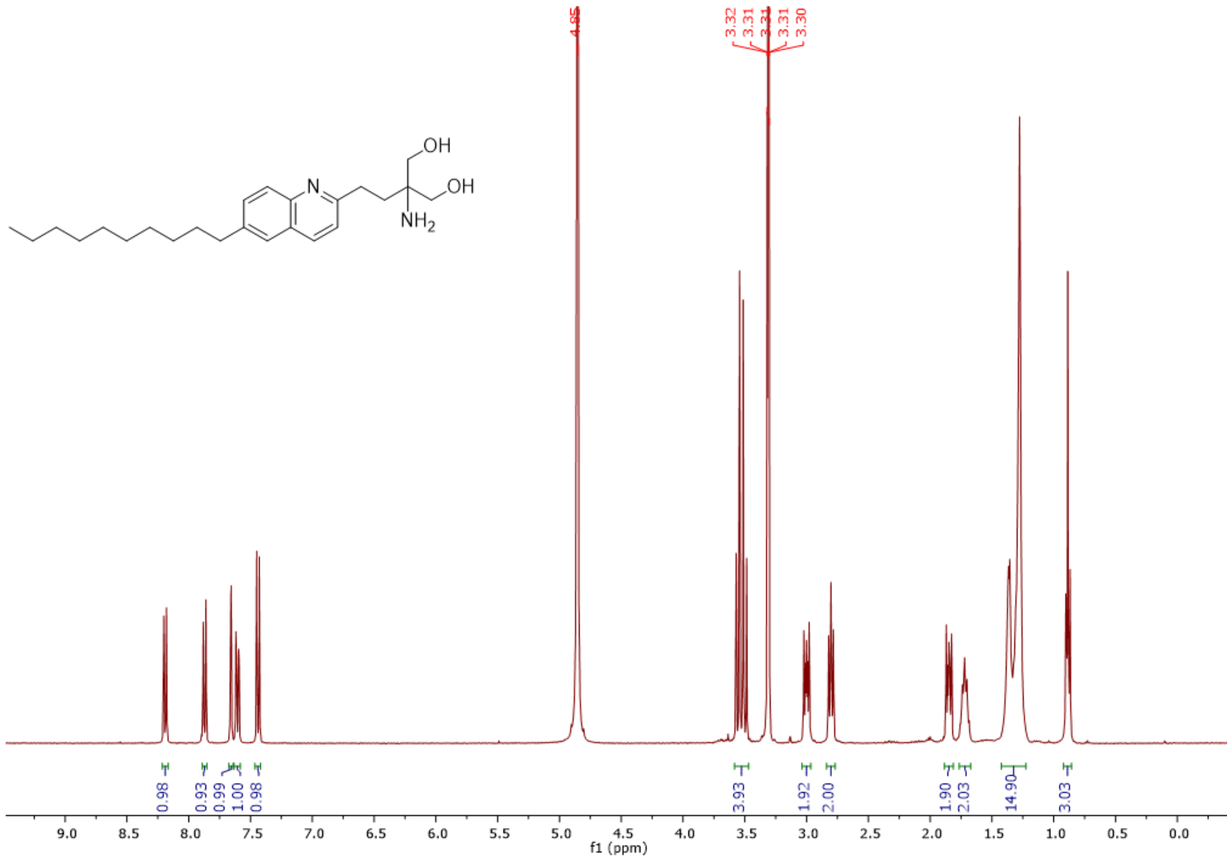

Figure S54: ^{1}H NMR spectra of compound **81** in CD_3OD.

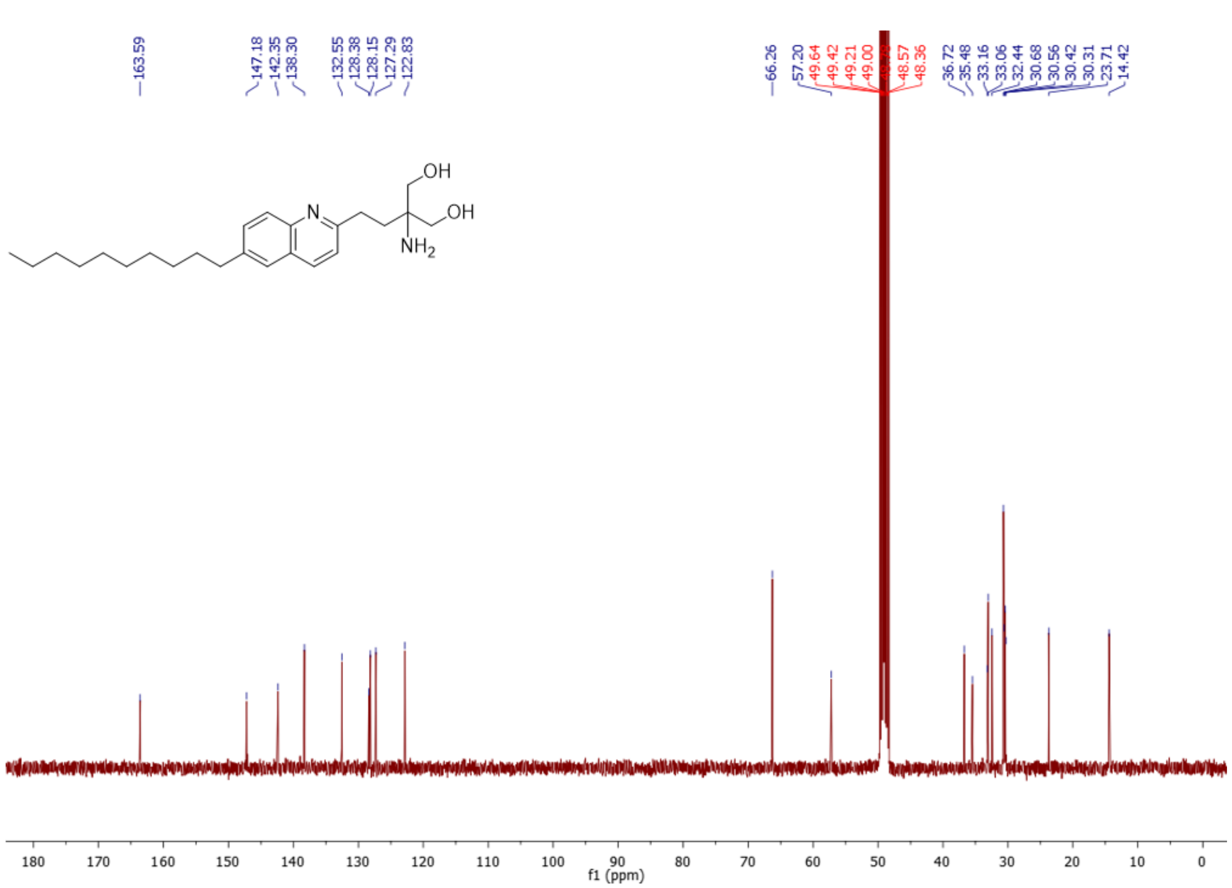

Figure S55: ^{13}C NMR spectra of compound **81** in CD_3OD.

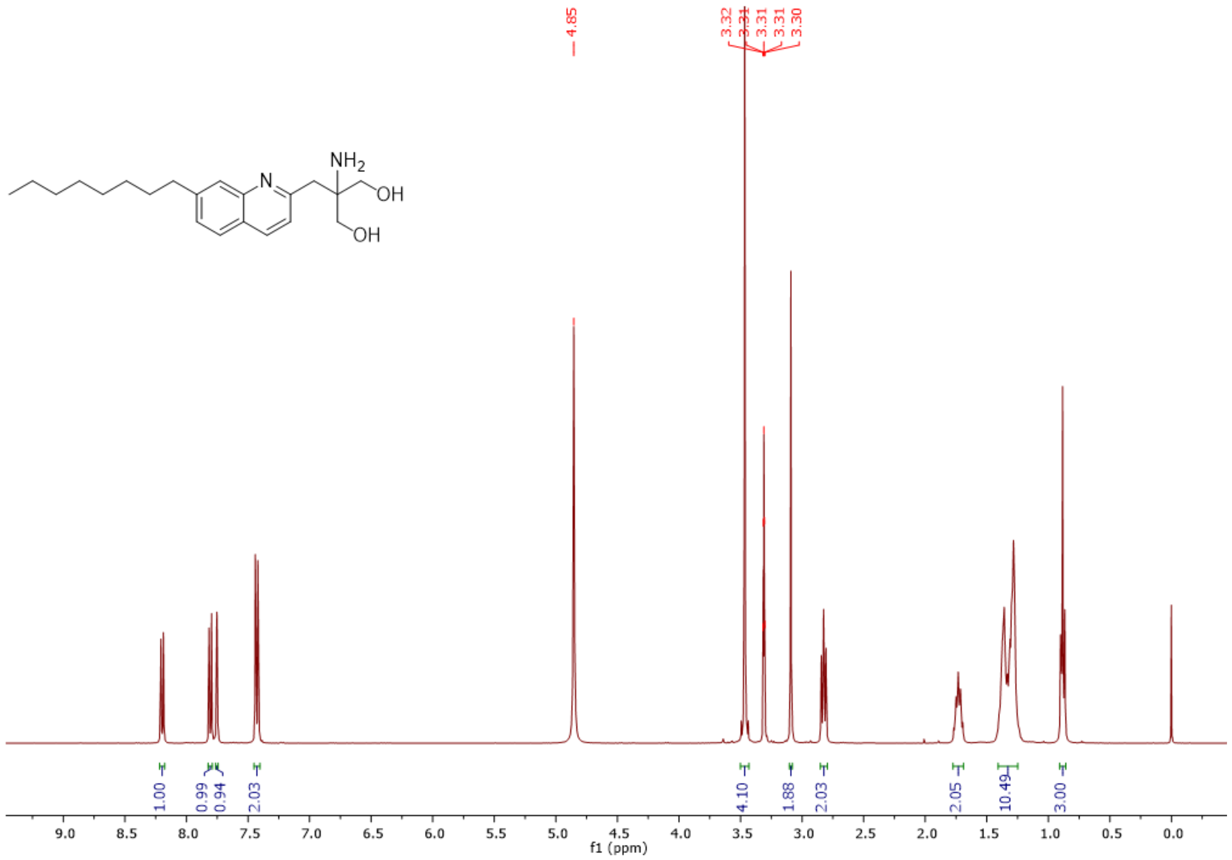

Figure S56: ^{1}H NMR spectra of compound **82** in CD_3OD.

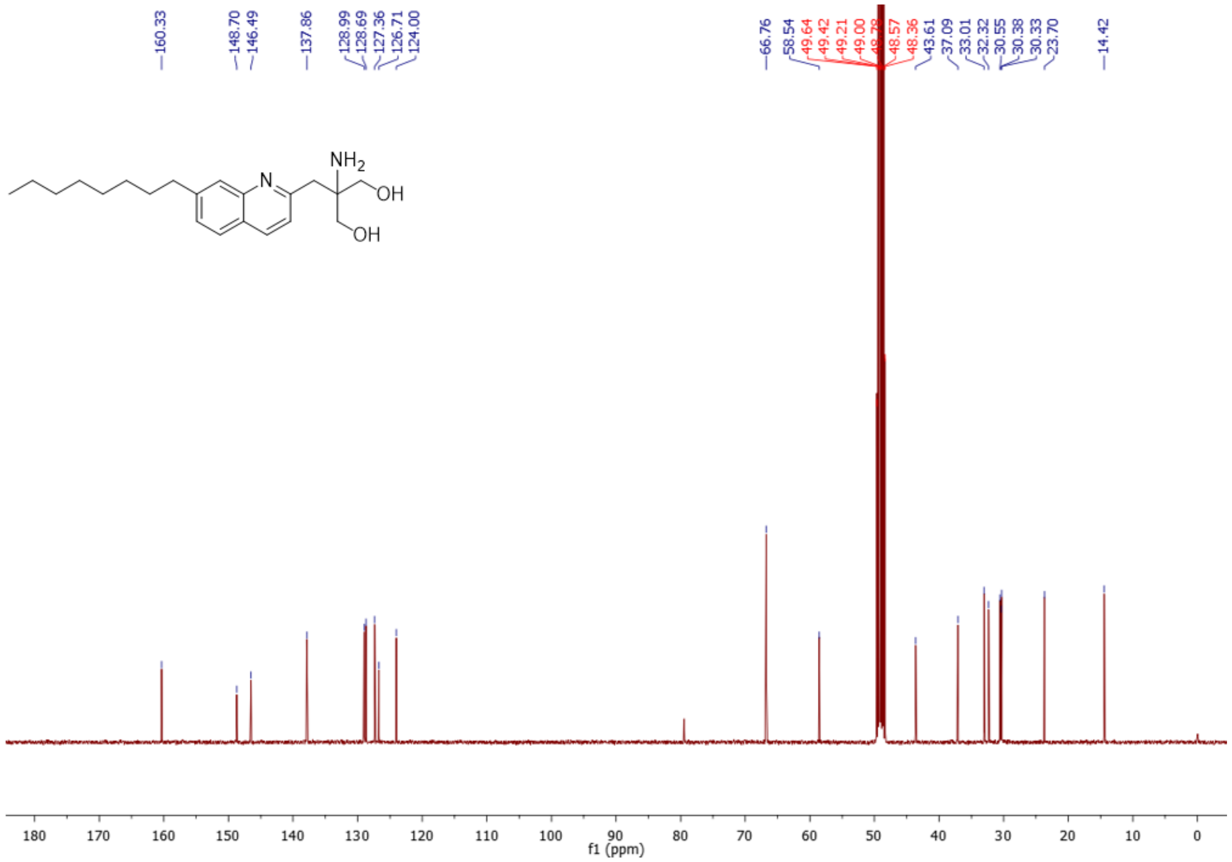

Figure S57: ^{13}C NMR spectra of compound **82** in CD_3OD.

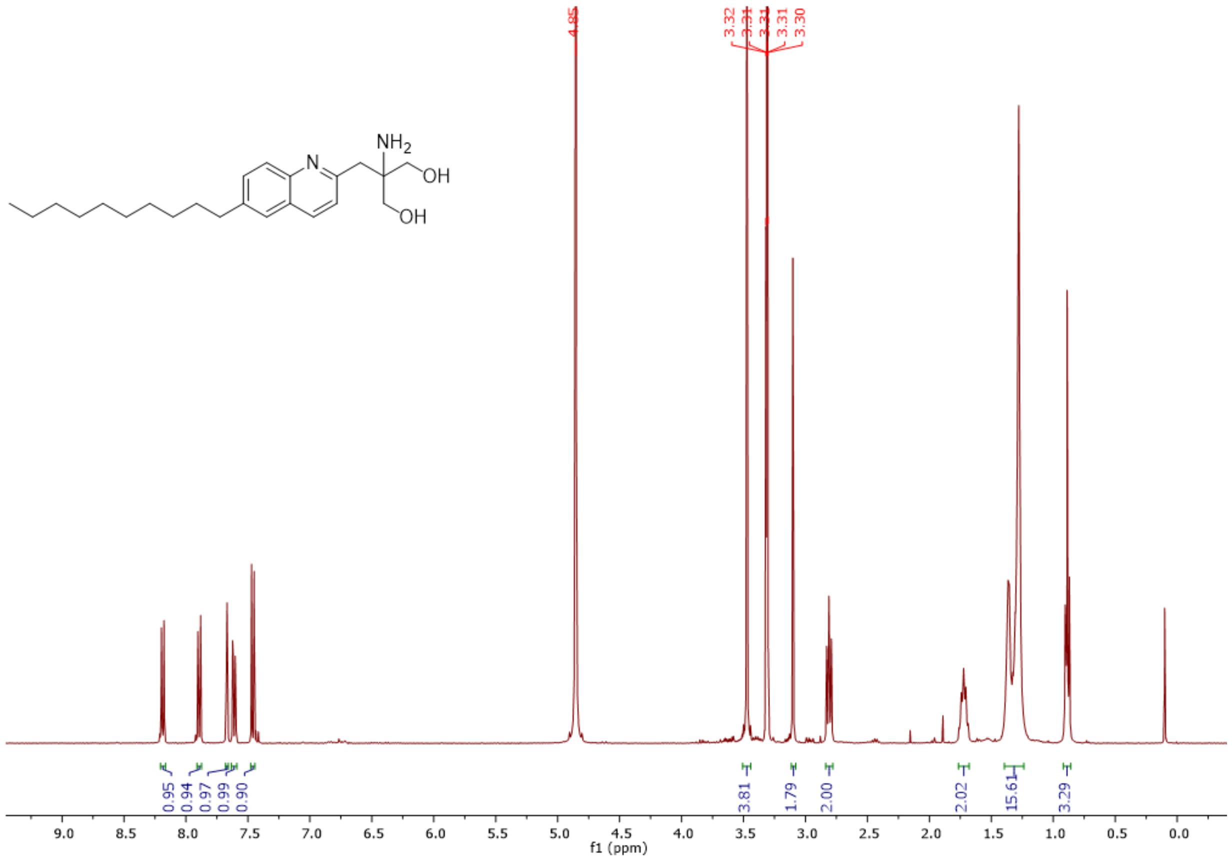

Figure S58: ^{1}H NMR spectra of compound **83** in CD_3OD.

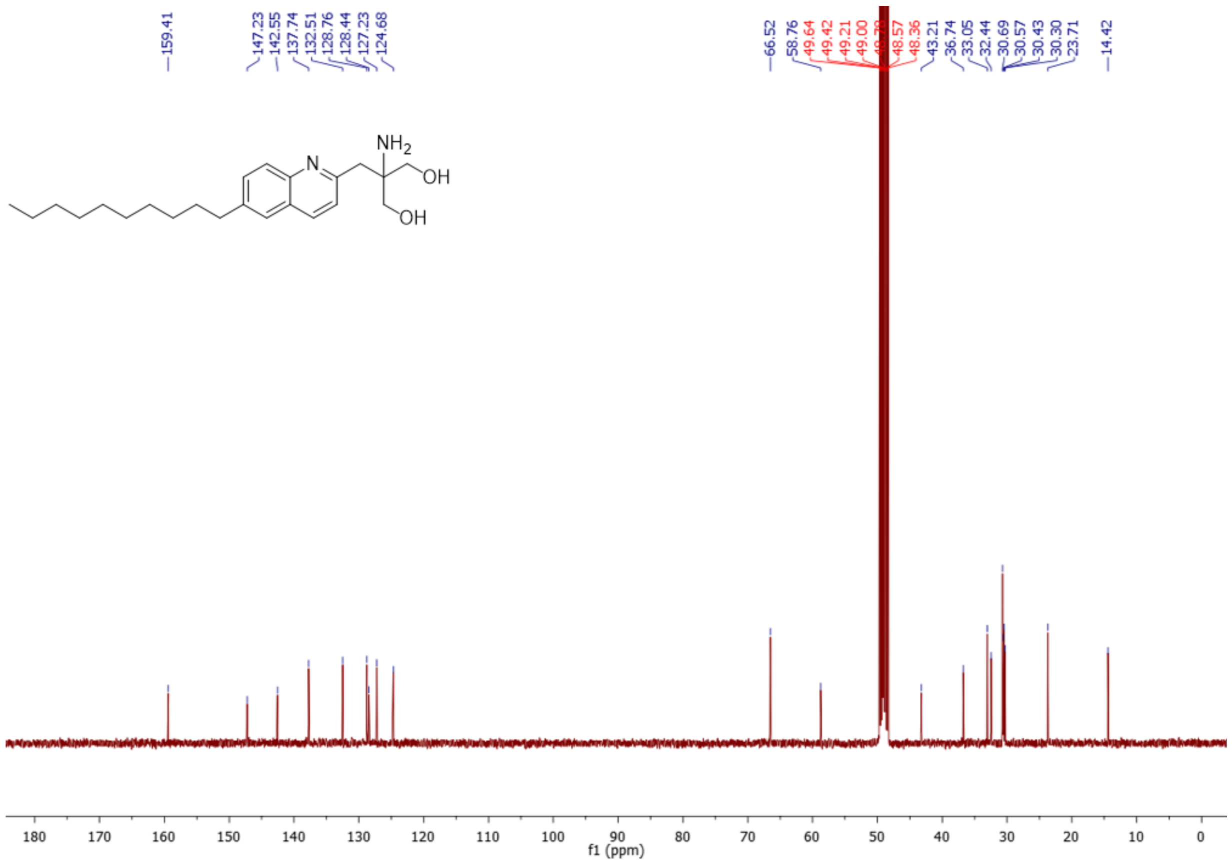

Figure S59: ^{13}C NMR spectra of compound **83** in CD_3OD.

5. Inhibition results against *S. aureus* ATCC 25923 (Table S1)

Table S1. Inhibition of *Staphylococcus aureus* ATCC 25923 in pre- and post-exposure by the active derivatives.

**Compound**		**Average inhibition %** [a]			
		Pre-exposure			
		Planktonic cells		Biofilms	
		Turbidity	*Viability*	*Viability*	*Biomass*
DMSO 1 %		10.8 ± 4.4	0 ± 0	2.8 ± 1.8	14.4 ± 6.4
Fingolimod	5 µM	25 ± 10.6	16.1 ± 26.5	38.7 ± 5.5	37 ± 11.2
	10 µM	36.7 ± 10	17.2 ± 23.4	41.5 ± 7.1	42.2 ± 5
	15 µM	99.8 ± 0.1	99.9 ± 0	99.7 ± 0.2	91.7 ± 6
	25 µM	99.8 ± 0.1	99.9 ± 0.1	99.5 ± 0.1	90.8 ± 6.9
**11**	5 µM	12.5 ± 12.4	17 ± 23.3	41.8 ± 9.7	40.1 ± 36.6
	10 µM	70.4 ± 40	73.3 ± 34.2	72.2 ± 23.8	75.6 ± 20.8
	15 µM	97.5 ± 1.9	98.5 ± 1.7	84.9 ± 24.9	87.1 ± 9.9
	25 µM	97.9 ± 1.2	99.2 ± 1.3	93.8 ± 10.1	89 ± 0.7
**15**	5 µM	44.9 ± 12.3	2.6 ± 4.5	42.5 ± 7.6	44.4 ± 15
	10 µM	70.3 ± 19.8	50.9 ± 46	71.3 ± 19.4	68.5 ± 20.6
	15 µM	72.5 ± 23.2	33.2 ± 57.6	70.4 ± 24.5	75.8 ± 18.7
	25 µM	76.5 ± 19.4	33.1 ± 57.3	79.3 ± 18.7	70.7 ± 26.8
**42**	5 µM	18.8 ± 12.6	1.7 ± 3	16.3 ± 4.8	41 ± 12.1
	10 µM	53.3 ± 32.3	31.6 ± 54.9	55.9 ± 31	62.5 ± 24.1
	15 µM	84.6 ± 26.1	66.4 ± 57.5	81.1 ± 32.3	80.5 ± 26.4
	25 µM	99.4 ± 0.5	99.6 ± 0.2	99.2 ± 0.8	95.1 ± 0.5
**43**	5 µM	25.6 ± 36.8	12.3 ± 21.4	17.4 ± 8.1	33.6 ± 16
	10 µM	36.2 ± 26.1	14.8 ± 15.3	29.1 ± 5.4	29.2 ± 14.5
	15 µM	61.4 ± 37.8	36.1 ± 55.2	52.5 ± 43.2	66.4 ± 27.2
	25 µM	98.9 ± 0.6	99.9 ± 0.2	99.7 ± 0	92.2 ± 6.9
**44**	5 µM	18.4 ± 6.2	0 ± 0	24.9 ± 12.6	23.2 ± 23.4
	10 µM	31.2 ± 8.9	0 ± 0	44.4 ± 4.9	36.6 ± 27.8
	15 µM	79.2 ± 28.2	66.8 ± 54.1	76.2 ± 29.5	76.6 ± 21.5
	25 µM	99.1 ± 0.7	100 ± 0.2	99.7 ± 0	93.1 ± 4.2
**45**	5 µM	45.7 ± 46.9	41.5 ± 50.3	45.4 ± 46.1	45.3 ± 46.5
	10 µM	99 ± 0.7	99.8 ± 0.1	98.9 ± 0.5	92.5 ± 4.8
	15 µM	98.9 ± 0.7	99.7 ± 0.1	99.7 ± 0.1	91.8 ± 4.7
	25 µM	98.6 ± 0.6	99.8 ± 0.2	99.7 ± 0	91.8 ± 5.6
**48**	5 µM	13.6 ± 13.1	0 ± 0	22.4 ± 5.4	28.7 ± 25.2
	10 µM	40.8 ± 18.5	20.3 ± 20.7	16.7 ± 15	31.9 ± 24.6
	15 µM	47.2 ± 50	36.9 ± 54.3	48.8 ± 44.2	53.1 ± 42.8
	25 µM	97.4 ± 2.6	99.7 ± 0.2	99.5 ± 0.8	92 ± 5.9
**49**	5 µM	25 ± 31.1	5.8 ± 6.5	21.8 ± 13.4	21.1 ± 15.7

	10 µM	40.2 ± 21.5	16 ± 20.8	19.2 ± 19.6	24.7 ± 21.7
	15 µM	32.7 ± 33.7	6.5 ± 7.4	34.9 ± 12.5	42.1 ± 13.8
	25 µM	52 ± 35.7	34.4 ± 41.1	46.4 ± 5.4	65 ± 8
**54**	5 µM	34.1 ± 32.1	11.9 ± 20.6	21.2 ± 4.7	21.2 ± 18.4
	10 µM	38.3 ± 27	10.2 ± 17.7	28.3 ± 15.2	23.7 ± 8.7
	15 µM	44.1 ± 23.3	8.5 ± 14.7	28.6 ± 10.1	39.3 ± 23.6
	25 µM	98.8 ± 1	99.8 ± 0.1	99.5 ± 0.6	93.7 ± 4.1
**55**	5 µM	38.2 ± 53.8	33.2 ± 57.6	50.6 ± 42	48.4 ± 41.1
	10 µM	43.5 ± 48.8	43.1 ± 49.4	66.7 ± 28.3	69.6 ± 23.5
	15 µM	99 ± 0.6	99.8 ± 0.1	99.2 ± 0.2	93.4 ± 1.1
	25 µM	98.8 ± 0.6	99.8 ± 0.2	99.6 ± 0.2	94.2 ± 1.1
**62**	5 µM	9.5 ± 10.5	0 ± 0	23.3 ± 7.1	34.5 ± 10.1
	10 µM	15.6 ± 7.5	0 ± 0	35.7 ± 5.8	46 ± 8
	15 µM	85.8 ± 12.4	92.4 ± 6.5	95.2 ± 3.8	91.8 ± 5.5
	25 µM	99.1 ± 0.7	100 ± 0.1	99.6 ± 0.2	94.8 ± 1.6
**79**	5 µM	15.2 ± 17.2	26.3 ± 24.3	28 ± 2.7	37.5 ± 32.5
	10 µM	98.7 ± 0.9	99.8 ± 0	98.1 ± 0.5	91.3 ± 5.8
	15 µM	98.7 ± 1	99.8 ± 0.1	99.3 ± 0.2	92.1 ± 4.9
	25 µM	98.5 ± 1	99.9 ± 0.2	99.2 ± 0.3	92.5 ± 4.2
**80**	5 µM	43 ± 17	13.9 ± 22.4	26.7 ± 15.1	24.2 ± 6.6
	10 µM	99 ± 0.7	99.4 ± 0	99.2 ± 1.4	97.6 ± 1
	15 µM	99.5 ± 0.3	99.5 ± 0	99.5 ± 0.5	97.3 ± 0.6
	25 µM	99.1 ± 0.3	99.7 ± 0	99.4 ± 0.3	92.3 ± 1.5
**81**	5 µM	33.4 ± 30.9	23.5 ± 26.1	28.3 ± 16.5	28.1 ± 33.9
	10 µM	57.3 ± 36.4	33.9 ± 57	64 ± 29.7	66 ± 27.6
	15 µM	99.3 ± 0.2	99.7 ± 0	99.1 ± 1	98.4 ± 0.3
	25 µM	98.6 ± 0.3	99.7 ± 0.1	99.1 ± 1.4	97.3 ± 0.1
**82**	5 µM	32.8 ± 33.4	6.1 ± 10.6	34.4 ± 13.6	41.7 ± 5.1
	10 µM	61.5 ± 33.3	33 ± 57.1	63.5 ± 30.9	67.2 ± 23.4
	15 µM	99.9 ± 0.1	99.6 ± 0	99.7 ± 0.3	94.4 ± 4.6
	25 µM	99.9 ± 0.1	99.7 ± 0	99.7 ± 0.4	93 ± 5.8
**83**	5 µM	37.9 ± 5.1	6.9 ± 10.7	34.6 ± 5.8	37.3 ± 7.9
	10 µM	99.5 ± 0.2	99.6 ± 0	99.6 ± 0.4	97.3 ± 0.8
	15 µM	99.4 ± 0.1	99.6 ± 0	99.8 ± 0.6	93.9 ± 1.4
	25 µM	99 ± 0.2	99.7 ± 0	99.6 ± 0.4	89.4 ± 1.4
**Compounds**		Post-exposure			
		Planktonic cells		Biofilms	
		Turbidity	*Viability*	*Viability*	*Biomass*
DMSO 1 %		10.5 ± 9.9	0.8 ± 1.6	8.6 ± 6	10.4 ± 0.8
Fingolimod	25 µM	1.1 ± 2	7.8 ± 3.6	19.2 ± 14.5	39.7 ± 16.2

	50 µM	13.8 ± 11.2	2.9 ± 2.6	35 ± 7	60.7 ± 12.4
	100 µM	42.4 ± 7	48.9 ± 16.2	52.2 ± 12.4	67.3 ± 9.5
	200 µM	75.5 ± 11	90.5 ± 9.9	77.3 ± 19	71 ± 11
**11**	25 µM	8.9 ± 8.2	0 ± 0	10.2 ± 8.5	32.6 ± 5.1
	50 µM	14 ± 4.2	0 ± 0	17.5 ± 9	35.4 ± 2.7
	100 µM	16.2 ± 8.6	6 ± 10.4	24.8 ± 10.2	48.9 ± 4.7
	200 µM	22.2 ± 12.7	30.5 ± 9.5	25.8 ± 8.3	47.6 ± 7.1
**15**	25 µM	16.8 ± 10	3.6 ± 6.3	19 ± 6.6	34.9 ± 12.7
	50 µM	14.7 ± 13.4	1.2 ± 2.1	30.8 ± 8.5	44.9 ± 6.2
	100 µM	17.9 ± 1.1	3.8 ± 6.6	31.9 ± 6.2	43.6 ± 7.8
	200 µM	47.4 ± 13.9	29.1 ± 25.2	38.7 ± 13.3	45.5 ± 18.5
**42**	25 µM	12 ± 9.6	2.8 ± 5	20 ± 6.1	39 ± 2.5
	50 µM	29.7 ± 16.3	19.1 ± 18.9	42 ± 16	54.1 ± 1.9
	100 µM	56.9 ± 19.5	68.7 ± 18.2	48.3 ± 2.3	54.8 ± 5.9
	200 µM	46.2 ± 27.3	35.1 ± 44.8	44.8 ± 10.4	59.8 ± 21
**43**	25 µM	6.1 ± 8	0 ± 0	14.4 ± 14	38.4 ± 9.2
	50 µM	33.5 ± 18.7	12.8 ± 18.3	23.1 ± 5.1	50.1 ± 4.6
	100 µM	48.9 ± 21.5	61.4 ± 27.6	35.4 ± 15.6	64.7 ± 9.1
	200 µM	79.1 ± 6.3	98 ± 3.3	75.7 ± 18.5	79.9 ± 12
**44**	25 µM	6.1 ± 10.6	3.8 ± 6.6	21 ± 11.5	36 ± 3.3
	50 µM	58.6 ± 10.2	56.1 ± 15.4	38.2 ± 8.2	51.6 ± 10.7
	100 µM	84.7 ± 3.3	99.8 ± 0.6	74 ± 21.5	70.5 ± 19.2
	200 µM	85.9 ± 5.3	100.1 ± 0.1	98.5 ± 0.1	92 ± 1.2
**45**	25 µM	9.2 ± 8.6	1 ± 1.7	15.5 ± 10.9	36.9 ± 3
	50 µM	28.1 ± 16.3	22.5 ± 18.5	25.2 ± 5.5	39.5 ± 9
	100 µM	45.7 ± 12.8	59.7 ± 16.4	23.1 ± 10.2	51.3 ± 5.8
	200 µM	53.9 ± 18.7	70.7 ± 12.5	17.1 ± 16.5	47.7 ± 12.7
**48**	25 µM	9.3 ± 10	0.9 ± 1.6	21 ± 14.6	47.6 ± 0.5
	50 µM	12.6 ± 10.9	0 ± 0	42.3 ± 5.5	57.8 ± 6.5
	100 µM	31.2 ± 10.9	21.3 ± 18.5	52.5 ± 9.2	65.9 ± 12.5
	200 µM	40 ± 13.2	48.3 ± 23.5	52 ± 9.4	72.5 ± 9.8
**49**	25 µM	1 ± 1.8	0 ± 0	23.1 ± 12	46.2 ± 2.6
	50 µM	6.6 ± 6.1	0 ± 0	31.6 ± 11.1	41.6 ± 16.2
	100 µM	18.3 ± 26.2	6 ± 10.4	30.4 ± 13.1	47.3 ± 10.1
	200 µM	45 ± 23.1	24.6 ± 9.6	18.9 ± 12.3	41.4 ± 16.7
**54**	25 µM	5.1 ± 6.4	15 ± 26.1	11.2 ± 0.8	44 ± 14.9
	50 µM	17.4 ± 3.4	0 ± 0	23.6 ± 12.7	55.3 ± 3
	100 µM	82.6 ± 4.7	97.9 ± 3.4	82.1 ± 27.3	83.5 ± 19
	200 µM	83.6 ± 3.7	100.1 ± 0.2	99.8 ± 0.2	86.4 ± 3.9
**55**	25 µM	15.7 ± 13.7	0 ± 0	17.9 ± 13.2	51.6 ± 18.4

	50 µM	9.8 ± 13.9	0 ± 0	36.8 ± 8.3	63.1 ± 9
	100 µM	67.6 ± 10.3	70 ± 12.5	44.4 ± 9.7	61.4 ± 3.4
	200 µM	84.2 ± 3.1	100.4 ± 0	94 ± 8.6	80.8 ± 4.2
**62**	25 µM	15.7 ± 12.5	0 ± 0	12.6 ± 12.7	43.1 ± 11
	50 µM	16.7 ± 24.2	0 ± 0	37.2 ± 4.9	63.9 ± 10.9
	100 µM	57.6 ± 29.3	55.8 ± 42.1	56.4 ± 12	76.2 ± 5.6
	200 µM	89.9 ± 1.7	100.1 ± 0.1	99.3 ± 0.3	80.8 ± 3.8
**79**	25 µM	16.1 ± 25.9	0.3 ± 0.6	15.4 ± 3.3	21.5 ± 4.7
	50 µM	12.4 ± 8.3	0 ± 0	23.3 ± 4.3	29.7 ± 10.3
	100 µM	25.7 ± 16.3	23.6 ± 15.5	28.7 ± 6.3	36.6 ± 6.4
	200 µM	31.5 ± 11.1	57 ± 23.8	28.8 ± 6.5	43.1 ± 20.9
**80**	25 µM	31.7 ± 26.5	17.5 ± 26.7	13.8 ± 11.7	25.1 ± 16.4
	50 µM	26 ± 39.1	21.5 ± 33.1	29.8 ± 10.2	50.2 ± 3.2
	100 µM	64.3 ± 18.5	81 ± 8	48.6 ± 5.2	61.9 ± 7.7
	200 µM	73.4 ± 9.8	93.2 ± 1.3	48.4 ± 12	51.5 ± 9.6
**81**	25 µM	22.7 ± 32.9	8.8 ± 7.7	6.4 ± 8.2	9.7 ± 8.6
	50 µM	10 ± 17.4	6.9 ± 10.4	18.3 ± 16.6	28.5 ± 12.5
	100 µM	19 ± 22.4	28.8 ± 17.2	20.9 ± 18.1	34.6 ± 8.2
	200 µM	11.2 ± 16.9	7.8 ± 11.1	20.1 ± 16	25 ± 11.6
**82**	25 µM	14.1 ± 11.7	0 ± 0	11.8 ± 8.4	45.6 ± 7.5
	50 µM	34.6 ± 28.4	17.9 ± 25.5	27.8 ± 14.6	51.6 ± 10.3
	100 µM	79.1 ± 11.5	94.5 ± 10	81.5 ± 32.8	79.6 ± 17
	200 µM	77.9 ± 10	100.5 ± 0.5	100.2 ± 0.2	79.8 ± 5.7
**83**	25 µM	27.1 ± 19.8	14.1 ± 20.9	16.8 ± 17.2	31.7 ± 20.9
	50 µM	28.3 ± 13.2	22.7 ± 19.5	34.4 ± 15.3	50.3 ± 6.4
	100 µM	64.4 ± 14	80.3 ± 17.9	56.6 ± 18.2	71.3 ± 10.2
	200 µM	65.3 ± 5.8	88.3 ± 12.1	58.6 ± 19.5	71.5 ± 5.8

[a] Results are averages from three separate experiments with two replicates each ± SD.

6. Inhibition results against the clinical strains of *S. aureus* (Table S2)

Table S2. Inhibition of *Staphylococcus aureus* ATCC 12598 and P2 in pre-exposure by selected derivatives.

**Compound**		**Average inhibition %** [a]			
		S. aureus ATCC 12598			
		Planktonic cells		Biofilms	
		Turbidity	*Viability*	*Viability*	*Biomass*
DMSO 1 %		5.5 ± 5.7	5.7 ± 8.1	17.6 ± 4.4	18.8 ± 9
Penicillin-G	50 µM	99.7 ± 0	99.7 ± 0	97.5 ± 1.5	95.8 ± 1
Fingolimod	5 µM	6.3 ± 8.7	2.9 ± 2.9	16.9 ± 2.9	18.5 ± 17.7
	10 µM	21.5 ± 27.1	34 ± 29.5	35.8 ± 17.9	43.2 ± 15.8
	15 µM	99.6 ± 0.1	99.7 ± 0	99.2 ± 0.5	94.5 ± 1.1
	25 µM	99.2 ± 0.1	99.7 ± 0	99.6 ± 0.3	95.2 ± 0.4
**45**	5 µM	89.3 ± 24.1	83.4 ± 39.6	83.6 ± 38.2	75.4 ± 28.1
	10 µM	99.4 ± 0	99.8 ± 0	99.6 ± 0.1	95.3 ± 0.4
	15 µM	99.1 ± 0	99.8 ± 0	99.6 ± 0.1	94.4 ± 0.3
	25 µM	98.3 ± 0.2	99.8 ± 0.1	99.6 ± 0.1	94.9 ± 1
**55**	5 µM	21.2 ± 6.5	7.6 ± 8.8	15.2 ± 6.4	27.6 ± 19.6
	10 µM	99.6 ± 0.1	99.8 ± 0	99.2 ± 0.1	94.3 ± 1.2
	15 µM	99.4 ± 0.2	99.7 ± 0	99.5 ± 0.3	93 ± 1.1
	25 µM	98.9 ± 0.3	99.6 ± 0.1	99.6 ± 0	90.2 ± 2.1
**79**	5 µM	82.3 ± 26.8	69.3 ± 47.1	65.9 ± 50.1	63.5 ± 41.5
	10 µM	99.6 ± 0	99.8 ± 0.1	99.6 ± 0.1	94.7 ± 0.2
	15 µM	99.4 ± 0.1	99.6 ± 0.1	99.5 ± 0	94.6 ± 0.6
	25 µM	99.2 ± 0.3	99.6 ± 0.1	99.5 ± 0.1	95 ± 1
**80**	5 µM	22.7 ± 5.4	7.3 ± 12.6	24.6 ± 6.8	34.3 ± 18.8
	10 µM	99.6 ± 0	99.7 ± 0	99.2 ± 0.3	96.5 ± 1
	15 µM	99.5 ± 0.1	99.6 ± 0	99.5 ± 0.1	93.4 ± 1.2
	25 µM	98.9 ± 0.3	99.6 ± 0	99.5 ± 0.2	89.4 ± 0.6
**81**	5 µM	2.1 ± 3.7	4.2 ± 7.3	15.6 ± 9.7	19.1 ± 26.4
	10 µM	99.8 ± 0.1	99.7 ± 0	99.4 ± 0	96 ± 0.6
	15 µM	99.7 ± 0.1	99.8 ± 0.1	99.7 ± 0.1	93.5 ± 1.8
	25 µM	99.3 ± 0.2	99.6 ± 0.1	99.7 ± 0.1	88.7 ± 2
**82**	5 µM	20.6 ± 8.5	13.3 ± 5	12.2 ± 12.8	26.3 ± 11.6
	10 µM	99.6 ± 0.1	99.8 ± 0	99.1 ± 0.2	94.4 ± 1.7
	15 µM	99.7 ± 0	99.7 ± 0	99.5 ± 0	97.1 ± 1.1
	25 µM	99.4 ± 0.2	99.6 ± 0.1	99.7 ± 0.1	94.6 ± 1.4
**Compounds**		*S. aureus* P2			
		Planktonic cells		Biofilms	
		Turbidity	*Viability*	*Viability*	*Biomass*
DMSO 1 %		24.6 ± 7.2	4.3 ± 6.2	1.9 ± 1.6	0.1 ± 0.2

Penicillin-G	50 µM	12.2 ± 14.5	14.9 ± 10.3	9.5 ± 9.8	17.4 ± 13.2
Fingolimod	5 µM	3.3 ± 5.8	4.3 ± 7.4	18.2 ± 17	34.9 ± 19
	10 µM	39.5 ± 45.1	45 ± 45.6	55.4 ± 38	64.3 ± 29.1
	15 µM	99.6 ± 0	99.8 ± 0	99.8 ± 0	98.3 ± 0.1
	25 µM	99.3 ± 0.1	99.7 ± 0	99.8 ± 0.1	98 ± 0.4
**45**	5 µM	74.4 ± 34.2	70 ± 36.1	69.6 ± 37.8	74.6 ± 24.7
	10 µM	99.4 ± 0.1	99.8 ± 0	99.6 ± 0	97.6 ± 0.3
	15 µM	99.1 ± 0.1	99.7 ± 0.1	99.6 ± 0	96.9 ± 1.4
	25 µM	98.6 ± 0.1	99.6 ± 0.1	99.6 ± 0	97.6 ± 0.3
**55**	5 µM	14 ± 3	3.4 ± 5.8	32.1 ± 8.5	23.6 ± 10.9
	10 µM	99.6 ± 0.1	99.8 ± 0	99.4 ± 0.2	96.5 ± 0.7
	15 µM	99.3 ± 0.1	99.6 ± 0.1	99.6 ± 0	95.1 ± 0.2
	25 µM	98.8 ± 0.2	99.5 ± 0.1	99.6 ± 0	91.6 ± 1.2
**79**	5 µM	45.4 ± 35.4	45.3 ± 32.9	49.4 ± 34.5	63.6 ± 23.6
	10 µM	99.5 ± 0	99.6 ± 0	99.4 ± 0	95.8 ± 2.2
	15 µM	99.4 ± 0.1	99.6 ± 0.1	99.4 ± 0	97.3 ± 0.5
	25 µM	99.1 ± 0.2	99.6 ± 0.1	99.5 ± 0	96.4 ± 1.3
**80**	5 µM	3.3 ± 5.4	20.6 ± 21.6	45.2 ± 2.1	57.3 ± 10.2
	10 µM	99.6 ± 0.1	99.7 ± 0.2	99.5 ± 0.1	95.4 ± 2.9
	15 µM	99.3 ± 0	99.6 ± 0.1	99.5 ± 0	95.3 ± 0.8
	25 µM	98.8 ± 0.2	99.5 ± 0.1	99.5 ± 0	90.6 ± 1
**81**	5 µM	0 ± 0	42.9 ± 19.7	37.3 ± 3.3	48.4 ± 15.7
	10 µM	99.8 ± 0.1	99.6 ± 0.2	99.6 ± 0.1	96.6 ± 0.4
	15 µM	99.7 ± 0.1	99.6 ± 0.1	99.6 ± 0	94.5 ± 0.6
	25 µM	99.2 ± 0.2	99.6 ± 0.2	99.6 ± 0	89.1 ± 0.6
**82**	5 µM	5 ± 2.2	1 ± 1.8	23.7 ± 8.8	39.9 ± 7
	10 µM	99.6 ± 0	99.8 ± 0	99.5 ± 0	97.6 ± 0.4
	15 µM	99.7 ± 0	99.6 ± 0	99.8 ± 0	97.2 ± 0.8
	25 µM	99.4 ± 0.1	99.5 ± 0	99.7 ± 0.1	96.4 ± 0.6

[a] Results are averages from three separate experiments with two replicates each ± SD.

7. Inhibition results against the gram-negative bacteria (Table S3)

Table S3. Inhibition of the gram-negative species *Acinetobacter baumannii* NCTC 13423 and *Pseudomonas aeruginosa* ATCC 9027 in pre-exposure by 16 fingolimod derivatives.

**Compound**		**Average inhibition %** [a]			
		A. baumannii NCTC 13423			
		Planktonic cells		Biofilms	
		Turbidity	*Viability*	*Viability*	*Biomass*
DMSO 1 %		5.5 ± 3.1	14.5 ± 5.1	0.6 ± 0.9	3.5 ± 4
Fingolimod	10 µM	0 ± 0	5.9 ± 4.9	0.2 ± 0.5	1.9 ± 3.9
	15 µM	71.1 ± 37.7	74.1 ± 38.2	63.6 ± 31.5	44.1 ± 26.9
	25 µM	98.8 ± 2	99.5 ± 0.3	97.6 ± 2.3	79.2 ± 13.4
	50 µM	98.5 ± 1.4	99.4 ± 0.4	98.4 ± 1.1	84.8 ± 7.8
**11**	10 µM	0 ± 0	11.8 ± 6	0 ± 0	5.1 ± 8.9
	15 µM	0 ± 0	13.2 ± 6.7	0.8 ± 1.4	0 ± 0
	25 µM	0 ± 0	10.3 ± 9	3.6 ± 5.6	5.1 ± 4.6
	50 µM	0 ± 0	0.1 ± 0.1	21.4 ± 1.1	15.9 ± 13.8
**15**	10 µM	0 ± 0	11 ± 1.5	0 ± 0	0 ± 0
	15 µM	0 ± 0	8.7 ± 2.5	1 ± 1.5	0.8 ± 1.4
	25 µM	0 ± 0	7.8 ± 6.9	2.8 ± 2.6	0 ± 0
	50 µM	0 ± 0	0 ± 0	7.1 ± 0.8	4.8 ± 4.9
**42**	10 µM	0 ± 0	13.5 ± 3.1	0.4 ± 0.3	2.4 ± 4.3
	15 µM	0.6 ± 1	6 ± 6.6	12.8 ± 7.2	6.2 ± 6
	25 µM	40.8 ± 42.7	33.1 ± 52.5	47.6 ± 37.1	18.2 ± 21.6
	50 µM	83.1 ± 17.5	81 ± 27.7	87.7 ± 9.8	67.2 ± 13.9
**43**	10 µM	0 ± 0	5.5 ± 0.6	0.2 ± 0.4	0.6 ± 1
	15 µM	99.1 ± 1.1	99.8 ± 0.1	99.2 ± 1.1	76.9 ± 3.5
	25 µM	99.1 ± 1	99.8 ± 0.2	100 ± 1.1	81.7 ± 1
	50 µM	98.3 ± 0.9	99.8 ± 0.1	98.7 ± 2.5	79 ± 3.8
**44**	10 µM	0 ± 0	12.3 ± 2.4	0.6 ± 1	0 ± 0
	15 µM	2.8 ± 3.7	9.2 ± 5.8	8.6 ± 7.8	5.6 ± 6.5
	25 µM	88.1 ± 19	83.5 ± 27.6	88.7 ± 15.2	69.4 ± 13.7
	50 µM	98.3 ± 1.2	99.8 ± 0.5	99.9 ± 1	86.2 ± 2.3
**45**	10 µM	0 ± 0	4.8 ± 2.9	2.1 ± 3.6	0 ± 0
	15 µM	0 ± 0	2.8 ± 3.8	0.5 ± 0.8	1 ± 1.8
	25 µM	0 ± 0	0 ± 0	2.7 ± 4	0 ± 0
	50 µM	0 ± 0	0 ± 0	6.5 ± 7.4	0.7 ± 1.3
**48**	10 µM	0 ± 0	5.1 ± 0.7	0 ± 0	0 ± 0
	15 µM	0 ± 0	0 ± 0	1.4 ± 1.2	0 ± 0
	25 µM	63.2 ± 30.2	48.6 ± 48.9	68.3 ± 27.4	38.9 ± 34.1
	50 µM	94.4 ± 2.3	97.8 ± 0.3	93.8 ± 1.3	56.9 ± 19.5

**49**	10 µM	0 ± 0	12 ± 4.3	0 ± 0	0 ± 0
	15 µM	0 ± 0	8.5 ± 10.6	0 ± 0	1.5 ± 2.4
	25 µM	0 ± 0	2.6 ± 4.5	0 ± 0	1.1 ± 1.9
	50 µM	1.2 ± 2.1	0 ± 0	9 ± 8.3	4.1 ± 7.2
**54**	10 µM	0 ± 0	4.7 ± 2.4	0 ± 0	2.3 ± 4.1
	15 µM	0 ± 0	6.4 ± 4.2	1.2 ± 2.1	1.5 ± 1.3
	25 µM	0 ± 0.1	3.3 ± 4.4	6.1 ± 6.8	6.1 ± 8.1
	50 µM	99 ± 1	99.4 ± 0.2	100.2 ± 0.8	81.2 ± 8.8
**55**	10 µM	0.8 ± 1.4	3.8 ± 3.3	1.8 ± 3.2	0.2 ± 0.4
	15 µM	97.2 ± 2.5	98.1 ± 2.3	92.7 ± 9.4	74.2 ± 18.9
	25 µM	99.5 ± 0	99.7 ± 0	100.6 ± 0.9	88.5 ± 6.6
	50 µM	99.1 ± 0	99.5 ± 0	101 ± 1	87.4 ± 4.1
**62**	10 µM	46 ± 39.6	37.7 ± 25.3	40 ± 33.9	21.3 ± 18.9
	15 µM	99.3 ± 0.9	99.8 ± 0.4	100.5 ± 1	85.4 ± 2
	25 µM	99.2 ± 0.9	99.7 ± 0.3	101.2 ± 1.3	83.1 ± 3
	50 µM	98.8 ± 0.9	99.7 ± 0.2	101.3 ± 1.2	79 ± 9.4
**79**	10 µM	0 ± 0	4.7 ± 4.7	0.3 ± 0.6	1.7 ± 3
	15 µM	0 ± 0	4.2 ± 4.8	0.7 ± 0.6	3.3 ± 5.7
	25 µM	0 ± 0	0 ± 0	1.2 ± 1.5	0.2 ± 0.4
	50 µM	0.3 ± 0.6	0 ± 0	6.5 ± 6.4	3.3 ± 5.1
**80**	10 µM	0.8 ± 1.5	7.2 ± 5.2	0 ± 0	1.6 ± 2.8
	15 µM	96.4 ± 3	98.9 ± 0.3	71.9 ± 26.2	53 ± 33.4
	25 µM	99 ± 0.9	99.6 ± 0.1	101.6 ± 1.6	84.3 ± 5.9
	50 µM	98.8 ± 0.9	99.5 ± 0.2	101.8 ± 1.3	74.7 ± 11.4
**81**	10 µM	0.2 ± 0.5	7.5 ± 7.6	0 ± 0	0 ± 0
	15 µM	0 ± 0	5.8 ± 6.2	0 ± 0	2.1 ± 3.6
	25 µM	0 ± 0	2.6 ± 2.1	0 ± 0	3.6 ± 6.2
	50 µM	0.5 ± 0.9	2.6 ± 3.8	0.8 ± 1.4	5.6 ± 5.9
**82**	10 µM	1 ± 1.4	10.4 ± 9	3.9 ± 5.3	13.3 ± 12.9
	15 µM	98.3 ± 2.5	99.5 ± 0.5	96.5 ± 6	74.7 ± 11.1
	25 µM	99.8 ± 0	99.7 ± 0	100.7 ± 1.1	88.6 ± 7.8
	50 µM	99.4 ± 0.1	99.6 ± 0	99 ± 1.3	82.6 ± 12.7
**83**	10 µM	0 ± 0	8 ± 5.8	0.7 ± 1.2	6.4 ± 9.4
	15 µM	0 ± 0	7.2 ± 6.6	1.2 ± 2.2	0 ± 0
	25 µM	11.8 ± 12.4	3.1 ± 3.1	5.6 ± 6.6	0 ± 0
	50 µM	88.3 ± 1.5	95.2 ± 1.7	68.9 ± 2.6	10.4 ± 10.2
**Compounds**		*P. aeruginosa* ATCC 9027			
		Planktonic cells		Biofilms	
		Turbidity	*Viability*	*Viability*	*Biomass*
DMSO 1 %		11.9 ± 7.4	13 ± 6.1	32.6 ± 13.2	10.3 ± 6.9

Fingolimod	50 µM	51.5 ± 24	43.9 ± 35.5	12.4 ± 13.4	30.1 ± 16.2
	100 µM	85.9 ± 3.6	92.5 ± 4.8	36.5 ± 23.1	36.9 ± 13.7
	150 µM	86.8 ± 3.4	94.2 ± 3.6	42.4 ± 25.3	40.7 ± 17.8
	200 µM	85.1 ± 3.8	95 ± 1.2	47.5 ± 22.6	45.4 ± 18.5
**11**	50 µM	22.2 ± 20.1	26.2 ± 4.1	0 ± 0	8.3 ± 5.9
	100 µM	20.4 ± 17.1	6.4 ± 11.1	0 ± 0	13 ± 13.3
	150 µM	7.6 ± 5.1	10.3 ± 9.3	0 ± 0	12.1 ± 11.1
	200 µM	13.4 ± 11.7	1.2 ± 2	0 ± 0	3.1 ± 4.2
**15**	50 µM	12.6 ± 9.1	22.3 ± 2	0 ± 0	2.2 ± 2.2
	100 µM	11.3 ± 12.7	12 ± 14	0 ± 0	3.7 ± 3
	150 µM	15.7 ± 13.7	5.5 ± 6.3	0.5 ± 0.8	4.4 ± 1.9
	200 µM	13.7 ± 14.3	7.2 ± 6.3	0 ± 0	12 ± 10.7
**42**	50 µM	16.3 ± 5.3	24.8 ± 10.1	3.9 ± 6.8	30.1 ± 8.8
	100 µM	37.1 ± 13.5	15.1 ± 18	1.5 ± 2.6	30.3 ± 0.8
	150 µM	45.1 ± 17.4	18.9 ± 31	0.9 ± 1.7	27.7 ± 2.1
	200 µM	53.5 ± 14.1	27 ± 45	0 ± 0	36.2 ± 10.3
**43**	50 µM	94.5 ± 0.8	98.1 ± 0.5	53.1 ± 41.1	52.1 ± 19.8
	100 µM	97.2 ± 2.5	99 ± 1.3	99.2 ± 2	82.4 ± 25.2
	150 µM	96.9 ± 1.2	99.4 ± 0.7	99.9 ± 0.9	89.4 ± 13.3
	200 µM	97.5 ± 0.3	99.9 ± 0.1	100.3 ± 0.5	94.3 ± 1.4
**44**	50 µM	28.4 ± 10.9	31.4 ± 12.8	9 ± 8	35.2 ± 19.9
	100 µM	93.7 ± 2.6	95.8 ± 3.5	43 ± 44.4	57.7 ± 30.4
	150 µM	92.2 ± 4.8	92.1 ± 9.3	42.2 ± 50.3	54.7 ± 35.2
	200 µM	88.5 ± 4.4	88.7 ± 10.2	34.5 ± 36.6	48.7 ± 28.5
**45**	50 µM	31.3 ± 5.3	24.4 ± 2.7	8.1 ± 10.9	5 ± 3.4
	100 µM	41.8 ± 6	30 ± 7.6	4 ± 6.6	2.5 ± 4.4
	150 µM	39.3 ± 3.3	36.5 ± 11.5	1.9 ± 2.9	1.6 ± 2.8
	200 µM	40.7 ± 6.2	22.9 ± 11.2	0 ± 0	0 ± 0
**48**	50 µM	18.6 ± 7.4	28 ± 9	27.9 ± 25.6	14.7 ± 10.8
	100 µM	83.9 ± 2.6	76.1 ± 19.4	20.3 ± 9.9	29.1 ± 19.9
	150 µM	85.6 ± 3.3	88 ± 8.4	17 ± 12.1	37.8 ± 2.9
	200 µM	87.8 ± 4.2	93.5 ± 0.6	12.2 ± 5.8	36.9 ± 6.7
**49**	50 µM	11.2 ± 9.7	11.2 ± 5	1.1 ± 1	5.1 ± 7.7
	100 µM	19.8 ± 7.4	11 ± 9.5	1.4 ± 1.7	2.5 ± 1.1
	150 µM	11.4 ± 10.1	10.3 ± 10.8	1.8 ± 2.2	2.8 ± 4.2
	200 µM	16.7 ± 3	8.8 ± 10.7	4.2 ± 7.4	2.2 ± 2.8
**54**	50 µM	76.9 ± 11.6	74 ± 11.2	22.2 ± 26.9	46.4 ± 22
	100 µM	98.2 ± 0.5	99.6 ± 0	98.9 ± 1.5	96.7 ± 1.1
	150 µM	94.4 ± 1.4	99.2 ± 0.8	97.5 ± 3.9	90.9 ± 7.2
	200 µM	94.1 ± 1.3	99.7 ± 0.1	99.7 ± 0.1	94.1 ± 1.7

**55**	50 µM	45.1 ± 5.7	31.2 ± 8.9	13.6 ± 11.8	6.4 ± 8.2
	100 µM	49.4 ± 8.3	30.3 ± 25.5	9.4 ± 9.2	10.8 ± 8.8
	150 µM	54.3 ± 13.8	40.3 ± 27.1	3.3 ± 4.6	12 ± 10.4
	200 µM	59.4 ± 7.3	27.1 ± 15.6	5 ± 5.6	3.1 ± 2.7
**62**	50 µM	35.6 ± 9.3	18.7 ± 18.1	13.5 ± 11.7	19.7 ± 9.7
	100 µM	58 ± 23	39.6 ± 45.8	13.6 ± 3.2	18.6 ± 19.2
	150 µM	84.5 ± 4.1	76.3 ± 19.2	27.7 ± 14.8	21 ± 3.9
	200 µM	92.5 ± 5	94.7 ± 4.6	61.8 ± 34.8	38.5 ± 47.1
**79**	50 µM	23.6 ± 9	3.9 ± 3.6	0 ± 0	1.2 ± 1.5
	100 µM	23 ± 3.8	1.9 ± 2.3	0 ± 0	3.6 ± 4.7
	150 µM	28 ± 2.4	4.2 ± 7.4	0.5 ± 0.9	6.1 ± 5.3
	200 µM	25 ± 2.8	7.6 ± 13.1	2.1 ± 3.6	0 ± 0
**80**	50 µM	48 ± 11.1	39.9 ± 11	9.8 ± 12.2	5.2 ± 5.2
	100 µM	55.5 ± 5.2	46.1 ± 11.6	10.5 ± 10	0 ± 0
	150 µM	42.1 ± 13.6	22.9 ± 1.7	14.3 ± 12.2	12.1 ± 10.7
	200 µM	55.3 ± 0.9	33.1 ± 16.1	15.3 ± 15.9	12.3 ± 19.1
**81**	50 µM	29.2 ± 5.8	10.9 ± 9.5	4.9 ± 5.2	3.6 ± 4.6
	100 µM	41.2 ± 11.2	20.3 ± 12.9	3.8 ± 4.9	0 ± 0
	150 µM	38.2 ± 12.2	16.3 ± 5.7	7.9 ± 11.7	4 ± 7
	200 µM	30.5 ± 8.3	17 ± 16.2	0.3 ± 0.5	0.7 ± 0.6
**82**	50 µM	46.8 ± 2.3	53.2 ± 3.8	6.4 ± 5.7	7.6 ± 7
	100 µM	51.6 ± 8.5	48.1 ± 18.4	8.3 ± 8.6	1.8 ± 2.7
	150 µM	61.3 ± 19.9	57.4 ± 19	3.5 ± 6	2.7 ± 4.8
	200 µM	75.7 ± 14.1	51.6 ± 41.5	31.5 ± 49.1	27.5 ± 24.4
**83**	50 µM	35.2 ± 3	10.5 ± 6.5	12.2 ± 21.1	6.4 ± 11.1
	100 µM	38.6 ± 10.1	14.8 ± 0.4	0.5 ± 0.9	2.1 ± 2.3
	150 µM	34.8 ± 16.4	20.2 ± 15	2.6 ± 3.8	1.3 ± 2.3
	200 µM	33.2 ± 7.3	16.3 ± 15.7	1.1 ± 1	3.7 ± 4.7

[a] Results are averages from three separate experiments with two replicates each ± SD.
